# CNSistent integration and feature extraction from somatic copy number profiles

**DOI:** 10.1093/gigascience/giaf104

**Published:** 2025-09-13

**Authors:** Adam Streck, Roland F Schwarz

**Affiliations:** Institute for Computational Cancer Biology (ICCB), Center for Integrated Oncology (CIO), Cancer Research Center Cologne Essen (CCCE), Faculty of Medicine and University Hospital Cologne, University of Cologne, 50931, Cologne, Germany; Berlin Institute for Medical Systems Biology (MDC-BIMSB), Max Delbrück Center for Molecular Medicine in the Helmholtz Association, 10115,Berlin, Germany; Institute for Computational Cancer Biology (ICCB), Center for Integrated Oncology (CIO), Cancer Research Center Cologne Essen (CCCE), Faculty of Medicine and University Hospital Cologne, University of Cologne, 50931, Cologne, Germany; Berlin Institute for Medical Systems Biology (MDC-BIMSB), Max Delbrück Center for Molecular Medicine in the Helmholtz Association, 10115,Berlin, Germany; BIFOLD - Berlin Institute for the Foundations of Learning and Data, 10587, Berlin, Germany

**Keywords:** cancer, data processing, SCNA, deep learning, cancer classification

## Abstract

**Background:**

Most cancers exhibit somatic copy number alterations (SCNAs)—gains and losses of variable regions of DNA. SCNAs play a key role in cancer adaptation through modulation of gene expression, deletion of tumor suppressor genes, or amplification of oncogenes. Systematic analysis of SCNAs is now a routine task in both the clinic and research and can help identify novel cancer genes, improve our understanding of cancer gene regulation, and enable us to accurately reconstruct cancer phylogenies. However, to conduct such analyses, SCNA profiles have to be integrated between samples, patients, and cohorts—often a nontrivial task, for which dedicated toolkits are lacking.

**Results:**

To fill this gap, we developed CNSistent, a Python package for imputation, filtering, consistent segmentation, feature extraction, and visualization of cancer copy number profiles from heterogeneous datasets. We demonstrate the utility of CNSistent by applying it to the following publicly available cohorts: The Cancer Genome Atlas, Pan-Cancer Analysis of Whole Genomes, and TRAcking Cancer Evolution through therapy (Rx). We compare the effect of sample preprocessing and different segmentation and aggregation strategies on cancer type and subtype classification tasks using various classification models. We also evaluate how well a classifier trained on one cohort generalizes to another. Lastly, we introduce 2 segment-based peak and outlier scores to investigate relationships between segments, between samples, and between cancer types. Using these scores, we investigate non–small cell lung cancer samples, highlighting that SOX2 amplification is the dominant copy number alteration in lung squamous cell carcinoma and the main distinction to lung adenocarcinoma.

**Conclusions:**

CNSistent is a general-purpose toolkit for integrated processing of SCNA profiles across many patients and cohorts. It is available at https://bitbucket.org/schwarzlab/cnsistent. The Research Resource Identifier for CNSistent is SCR_027025.

## Introduction

Somatic copy number alterations (SCNAs)—gains and losses of long regions of DNA—are found across almost all cancer types and are one of the key defining features separating cancer cells from normal cells [1]. It has been demonstrated that quantifying SCNAs has predictive value in the clinic for both progression-free and overall survival [2, 3] and that they can serve as sensitive biomarkers for cancer classification and subtyping [4]. We and others have shown that many cancers demonstrate ongoing chromosomal instability and continuously accumulate SCNAs throughout their evolution [5], and SCNAs are excellent markers for inferring cancer evolution [6, 7]. Recently, copy number signatures have linked SCNAs to their underlying molecular mechanisms, further strengthening their prognostic value [8, 9].

SCNA profiles are commonly derived from a variety of experimental techniques, including single-nucleotide polymorphism (SNP) arrays, whole-exome and whole-genome sequencing [10], and recently also increasingly from single-cell sequencing [11]. One major advantage of SCNAs over other genomic data types, including somatic single-nucleotide variants, is ease of handling. Due to their aggregate nature, SCNA profiles of individual patients can be published without concern for privacy and the resulting access restrictions, leading to a growing set of publicly available and easily accessible samples from large cohorts such as The Cancer Genome Atlas (TCGA), the International Cancer Genome Consortium (ICGC) [12], and the TRAcking Cancer Evolution through therapy (Rx) (TRACERx) [13] lung and renal cancer cohorts.

Unfortunately, copy number profiles, typically defined as lists of segments with given start and end positions and copy number states, are not directly comparable across samples, patients, or cohorts. For example, for phylogenetic reconstructions within a patient, profiles have to undergo minimum consistent segmentation where breakpoints are shared between samples to enable evolutionary comparisons [6, 7]. For machine learning classifiers, profiles are often aggregated in fixed-width bins or on the gene level. Additionally, different experimental techniques and different copy number calling algorithms can lead to specific biases, missing data, and varying resolutions, further complicating the matter.

To foster reproducible research and avoid reimplementation of common tasks, a tool that enables integration and joint segmentation and thereby caters to the specific demands of copy number profiles would be desirable. To our knowledge, the only available tool that does not require access to the raw sequencing data is the web-based application CNApp [14], which, due to its web-based nature, is not easily integratable into data science workflows and was not available at its hosted site at the time of this writing.

To fill this gap, we here present CNSistent, a Python package for preprocessing, consistent segmentation, integration, statistical analysis, and visualization of SCNA profiles coming from heterogeneous data sources. We demonstrate the utility of CNSistent by integrating available copy number profiles from the TCGA, Pan-Cancer Analysis of Whole Genomes (PCAWG), and TRACERx cohorts. We evaluate various segmentation strategies, comparing the performance of deep learning–based multiclass cancer classification tasks and the classification of non–small cell lung carcinomas (NSCLC), and demonstrate the use of CNSistent for enabling phylogenetic inference from copy number profiles using the minimum consistent segmentation algorithm.

## Methods

**Figure 1:**
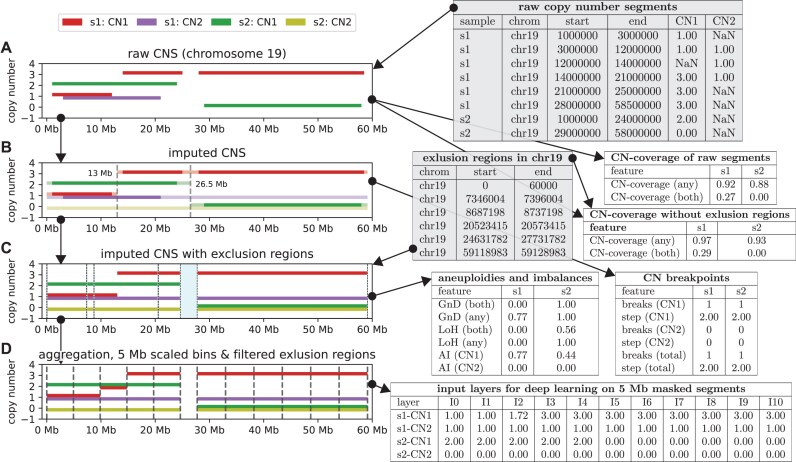
Illustrative example of processing of 2 SCNA profiles (s1, s2) for 2 alleles (CN1, CN2) on human chromosome 19. (A) The input data (gray tables) consist of noncontiguous major and minor copy number segments for each sample. From this, the proportion of the genome that is missing is calculated for each sample. For comparison, the CN-coverage is calculated both with and without considering the gap regions. Note that as there are no minor CNs for s2, the homozygous CN-coverage is 0. (B) During imputation, 2 new breakpoints are introduced at 13 Mb and 26.5 Mb, while the breakpoints on the boundaries of missing segments are no longer present. From the imputed data, CNSistent calculates the CN breakpoint-related statistical features. (C) Ploidy and allelic imbalance–related statistical features that are derived from the imputed data and removal of the gap regions. (D) Small regions are not used in region exclusion, retaining only the gap between 20 and 30 Mb, which splits the chromosome into 2 arms, which are then further split into ∼5-Mb bins. The same-size strategy is used, meaning that the bins in the left segment are slightly smaller (4.9 Mb), while the ones on the right are slightly bigger (5.27 Mb). Each profile is then converted into a vector of CN values for downstream analysis. Note that as there was a breakpoint at 13 Mb, the resulting value is a weighted mean of the previous values (i.e., 1.72).

CNSistent (RRID:SCR_027025) processes SCNA profiles using a multistep approach. Input data take the form of copy number segment tables with either allele-specific or total copy numbers (Fig. 1A). The processing is identical for both allele-specific and total copy numbers, but some of the statistics are limited in the case of copy numbers, as detailed below. Optionally, *exclusion regions* can be provided to the pipeline to remove locations in the genome where we expect lower quality of information. CNSistent first calculates the proportions of the missing genome, which we here refer to as *CN-coverage*, and then utilizes imputation strategies to fill in missing data (Fig. 1B). CNSistent then calculates information about breakpoints in each sample. Using the imputed data here has the advantage that spurious breaks are not created between nonconsecutive regions purely by missing data. Once the data are imputed, we remove the exclusion regions and calculate statistics relating to aberrant copy number values. In the final step, CNSistent offers various strategies for creating a consistent segmentation across samples (Fig. 1D), which are subsequently aggregated to create a final set of complete SCNA profiles with shared segment boundaries for all samples. The pipeline is fully modular, and the steps can be skipped or executed in a different order. Note that we use the term *segmentation* to refer to a consistent segmentation between samples (i.e., a set of positions inside each chromosome that split the chromosome into segments).

For its calculations, CNSistent can work with any reference genome; hg19 and hg38 reference assemblies are provided as a default. If the sex of the donors is not provided, CNSistent will determine the sex for each sample based on the presence of the Y chromosome.

### Imputation of missing values

SCNA profiles from different cohorts often vary in the extent to which they span the genome. This can be due to a variety of reasons, including different underlying technologies (whole-exome sequencing [WES] vs. whole-genome sequencing [WGS]), different segmentation strategies, or different exclusion of regions surrounding the centromeres and telomeres. To retain as much information as possible, CNSistent offers an imputation step capable of filling the gaps in SCNA profiles using an *extension* method (Fig. 1C).

The *extension* imputation method executes the following 5 steps: (i) Segments are pruned such that they are fully contained within the coordinates and named chromosomes of the reference genome. (ii) CNSistent extends the first and last segment of each chromosome to the chromosome boundaries. (iii) Each gap between 2 segments is split into 2 halves (rounded down), and each half is then assigned the copy number (CN) of its neighboring segment. (iv) If any chromosomes are fully missing from the sample, they are set to 0. (v) The neighboring segments that have the same CN are merged.

Alternatively, 2 additional imputation options are available: *diploid* and *null*. The diploid method changes the steps (ii–iv) in such a way that all newly created segments are set to diploid; for example, if a sample is male and major/minor CN columns are used, CNSistent will create a segment on the whole chromosome Y with major and minor CN of 1 and 0, respectively. The null option will analogously fill all the newly created segments with 0.

### Feature extraction

CNSistent can calculate a set of statistical features. As CNSistent is sex chromosome aware, the length of the linear genome depends on the sex of the sample. Each feature is therefore calculated 3 times: for autosomes, for sex chromosomes, and for the whole genome:

**CN-coverage**: Calculates the proportion of the whole genome where any CN value is assigned (as opposed to missing values). In case of allele-specific CNs, both monoallelic CN-coverage (either allele has a CN value assigned) and biallelic CN-coverage (both alleles must have a CN value assigned) are calculated.
**Genome not diploid (GnD):** Defines the proportion of the genome where an allele does not have the CN that a diploid cell of the same sex would have. In case of having only total CN, this is a lower-bound approximation.
**Loss of heterozygosity (LoH):** Calculates the proportion of segments with CN = 0 on either allele (hemizygous) or on both alleles (nullizygous). The segment is only considered LoH if and only if its CN value is 0 and its normal value is not zero (e.g., chromosome Y for female samples).
**Allelic imbalance (AI):** The proportion where 1 allele has a strictly higher CN than the other.
**Breakpoints:** The number of breakpoints per chromosome for each allele. If a 2-column format is used, the total number of breakpoints is also calculated to account for cases where both alleles have a breakpoint in the same location (meaning that the total number of breakpoints is less than the sum of the alleles).
**Breakpoint step:** The mean difference between the CNs of consecutive segments. Note that it is preferable to impute the segments first to avoid inducing spurious gaps.

### Consistent segmentation

One major goal of CNSistent is to obtain segments that are consistent between sample sets and from which features can then be derived in a unified manner. This requires the same set of breakpoints to be present in every sample. Segmentation consists of the following 4 steps: (i) define regions of interest (e.g., whole chromosomes, coding genes, etc.), (ii) remove exclusion regions (e.g., telomeric or centromeric regions), (iii) share existing breakpoints between samples and merge them based on a distance threshold, and/or (iv) subdivide the segments into fixed-width bins. Each of the 4 steps is optional.

The segments for step (i) can be provided as a BED file, or 1 of 5 predefined options can be used: whole chromosomes (default option), chromosome arms, cytobands, COSMIC consensus cancer gene set [15], or the Ensembl coding genes set [16]. From these segments, exclusion regions can be optionally removed (Fig. 1D). As a default option, the regions of low mappability, as defined by the UCSC [17] genome browser, are provided. During the exclusion process, if the regions are small or close to each other, fragmentation can occur. This can be avoided by segment filtering—the user specifies a filter of size f, where any exclusion region smaller than f is removed; likewise, if after the exclusion regions are removed from segmentation, any newly created segments smaller than f are also removed.

The breakpoints are then merged using a greedy algorithm on a predefined region (usually a whole chromosome). Starting from the leftmost breakpoint, all breakpoints within the merge distance $m$ are accumulated, and a new breakpoint is created at their average. This is then repeated from the leftmost not yet merged breakpoint, until the end of the region is reached. A detailed example is shown in Supplementary Fig. S1.

Lastly, the resulting segments can be subdivided into smaller bins based on user-defined split size $s$ (Fig. 1E). Three subdivision strategies are provided: (a) From the start of the segment, breakpoints are inserted every $s$ bases. Here, the last bin is likely to be of a different size. If it is smaller than $s/2$, it is merged with the previous segment. (b) This is similar to (a), where instead of creating the padding only at the end, the padding is split in half and added to both ends. Likewise, if the first and last bins are smaller than $s/2$, they are merged with their neighboring segments. (c) The bins are scaled so that they are all the same length, slightly different from $s$. Consider a segment that has $c$ bins, including the padding. If the padding is smaller or equal to $s/2$, split the segment into $c - 1$ equally sized bins, otherwise into $c$ bins.

### Aggregation of copy numbers

After joint segmentation, the copy numbers from the original segments are aggregated to create CNs for the new segments. First, the old segments are split at the breakpoints given by the new segmentation. Second, the resulting refined segments are aggregated between the breakpoints given by the segmentation, using 1 of 4 possible aggregation strategies: the *Min* and *Max* strategies will assign the minimum or maximum CN to the whole segment—the *Min* strategy is particularly relevant when considering genes, since incomplete segments are unlikely to yield functional gene copies. The *Mean* strategy will take a mean of CNs across bins weighted by their lengths, preserving the overall CN per sample. Lastly, merging can be skipped altogether, which can be used if we want to select only a subsection of each profile (e.g., only q-arms).

### Sample filtering

The features obtained in the feature extraction step can be used to filter undesirable samples. For base quality metrics, like CN-coverage, a simple *z*-score outlier detection method is provided, meaning that for a feature $f$ over a set of samples $S$, $z\; = \;\frac{{f( S )\; - \;\mu ( {f( S )} )}}{{\sigma ( {f( S )} )}}$ is calculated, and samples greater than 3 standard deviations from the mean ($| z |\; \ge 3$) are removed. The value 3 is a typical threshold for the method, but it can be adjusted by the user.

In certain cases, a qualitative separation of data is preferable (e.g., to remove samples with negligible SCNA activity). CNSistent offers an automated solution for finding such thresholds using a knee-detection algorithm. A knee point is where the maximum angle occurs between the line connecting the first point and the last point of the plot. To find the knee-points for a feature $f$ in a set of samples $S$, a tuple of monotonically increasing feature values $T = (min(f( S ),\cdots,\; max( {f( S )} ),\;\forall i\; \epsilon 1,\;| T |\; - \;1:\;{t_i}\; \le \;{t_{i + 1}}$ and a cumulative distribution of values smaller than each threshold $Y\; = \;{(| {f( S )\; \le \;t} |)_{\{ {t\;\epsilon T} \}}}$ is created. Second, $\;T$ is normalized such that $\forall t\epsilon T:\;t^{\prime} = \frac{{t - {t_1}}}{{{t_n} - {t_1}}},$ and analogously for $Y^{\prime}$. The knee-point is then the ${t_i},1\; \le i\; \le n\;$ with the maximum angle between the vector from origin to the normalized threshold, $({t_i}^{\prime},\;{y_i}^{\prime})$, and the vector from the threshold to the endpoint, $(1 - {t_i}^{\prime},1 - {y_i}^{\prime})$. If the angle is negative (clockwise rotation), we call it a *knee*; otherwise (counterclockwise rotation), we call it an *elbow*. A visualization of the method is provided in Supplementary Fig. S2.

### Outlier detection

CNSistent can detect outlier samples based on the normalized Manhattan distance (NMD) between pairs of samples. To calculate NMD, we normalize each sample by dividing the value of each bin by its sum. This normalization allows us to ignore the effects of whole-genome doubling (WGD), since the normalized values are the same before and after WGD. Formally, having 2 aggregated samples $S\; = \;( {{s_1}, \cdots ,{s_n}} )$ and $R\; = ( {{r_1}, \cdots ,{r_n}} )$, the $NMD( {S,\;R} )\; = \;\mathop \sum \nolimits_{j = 1}^n | {\frac{{{s_i}}}{{\Sigma ( S )}} - \frac{{{r_i}}}{{\Sigma ( R )}}} |.$ To compare a sample ${S_{}}$ to a cluster of samples $C\; = ( {{S_1}, \cdots ,{S_m}} )$, we calculate the outlier score $OS( {S,C} )\; = \;\frac{{\mathop \sum \nolimits_{j\epsilon 1}^m NMD( {{S_i},{S_j}} )}}{{| C |}}$. To compare between 2 cancer types $C1,\;C2$ and a sample $S\epsilon \;C1$, we extend the outlier score as $OS( {S,\;C1,\;C2} )\; = \;OS( {S,\;C2} )\; - \;OS( {S,\;C1} )$.

### Peak detection

To find regions of interest in the samples, CNSistent provides the peak score (PS), which shows how much each bin differs from its neighbors. With an aggregated sample $S\; = \;( {{s_1}, \cdots ,{s_n}} )$, we set the boundary values ${s_0}\; = \;{s_1},\;{s_{n + 1}}\; = \;{s_n}$ and calculate $\forall i\;\epsilon ( {1, \cdots ,\;n} ):\;PS( {S,\;i} )\; = ( {{s_i}\; - \;{s_{i - 1}}} )\; - ({s_{i + 1}} - {s_i})$. This score will be positive for segments higher than their neighbors and negative for those lower and close to zero for segments with monotonous behavior. We therefore use the PS to detect the highest and lowest values, which show the locations of most abrupt change in CN accumulation. Note that for meaningful calculation, this requires that the segments are connected to each other and about the same size.

### Identifying discriminatory features

To identify features that most differ between groups of samples, we use the Mann–Whitney *U* test using the mannwhitneyu function in SciPy v1.15.0. All *P* values are corrected for multiple tests using the multipletests function with Benjamin–Hochberg correction in statsmodels v0.14.0.

### Machine learning

To evaluate how different filtering and segmentation strategies affect the downstream analysis, we used 2 cancer type classification tasks: classifying between 6 types with the most samples, as introduced in Attique et al. [18], and the NSCLC classification, as introduced in Qiu et al. [19]. In this task, each binned sample, as illustrated in Fig. 1, represents 1 feature vector. The output probability is that a sample belongs to each cancer class under consideration. We then compare 4 different classification methods: random forest (RF), elastic net (ENet), deep neural network (DNN), and convolutional neural network (CNN).

For each of these models, we apply 5-fold cross-validation, that is, we split each dataset into 5 groups and always withhold one while training on the other 4. The validation accuracy for each model is then the mean of the test scores of the 5 different splits [20].

As the number of patients per cancer type varies, the classes are imbalanced. To avoid a possible bias due to an overrepresentation of 1 class, a stratified split is used, meaning that the ratio of the individual cancer classes is preserved across the 5 subsets. Additionally, some samples are obtained through multiregion sampling. While the samples from different regions show different profiles, there is a risk of being able to guess the class based on the similarity to the original profile. This is prevented by sample grouping, where each group (in this case, patient) can only be part of 1 subset. The splitting is done using the StratifiedGroupKFold object from scikit-learn v1.4.1, which was also used for ENet and RF classifiers.

For ENet, we used the SGDClassifier with log loss and the elasticnet penalty. For RF, we used RandomForestClassifier with default parameters. For deep learning, we used CNN and DNN3 neural network architectures, as described in Attique et al. [18], as well as our own extended CNN, which we called CNN+. In summary, the CNN uses 2 convolutional layers (kernel = 5) and RelU activation, followed by maxpool, batch normalization, and dropout after each, followed by a flattening and the output layer with softmax. The DNN3 uses 3 hidden layers with sizes 600, 300, and 150, with batch normalization, dropout, and RelU, except for the output layer, which uses softmax. The following was not declared in Attique et al. [18] and therefore has been set to default PyTorch values: maxpool kernel size of 2 and dropout probability of 0.5. Our CNN+ model builds on the CNN, but an additional fully connected layer is added after the flattening layer, with a size half in between the flattening and output layer. The CNN+ also uses 2 separate input channels, 1 for each allele. The full architecture of CNN+ is given in Supplementary Fig. S3.

Optimization was done using the PyTorch library v2.2.1 [21], accelerated using CUDA v12.1. Optimization was conducted using the Adam optimizer with a learning rate of 0.001, weight decay of 0.01, and batch size of 64. The error is evaluated using cross-entropy loss. The training was limited to 1,000 epochs. The training process was accelerated by an early stopping strategy, where the minimum loss is recorded. If, after 10 epochs, the training loss exceeds the existing global minimum, the training stops. To accommodate for the 2 alleles, we concatenated the major and the minor CNs into a single vector.

## Results

We illustrate the use of CNSistent on a cancer type classification task using 15,072 publicly available SCNA profiles from TCGA [9] (*n* = 10,674), PCAWG [12] (*n* = 2,778), and the TRACERx cohort of non–small cell lung cancer [13] (*n* = 1,620).

Where the TCGA and PCAWG datasets overlap (829 samples), we gave preference to the PCAWG callset. The PCAWG dataset blacklists 195 low-quality samples, which were removed before further processing. The TRACERx dataset consists of 2 parts, primary tumor samples (*n* = 1,428) and primary with metastatic samples (*n* = 694). We used the primary sample set in the 502 samples on which they overlap. This yielded a total set of 14,174 SCNA profiles that were subjected to CNSistent for preprocessing and integration. A summary of sample counts is provided in Supplementary Table S1.

### CNSistent segmentation of 14,174 copy number profiles

**Figure 2:**
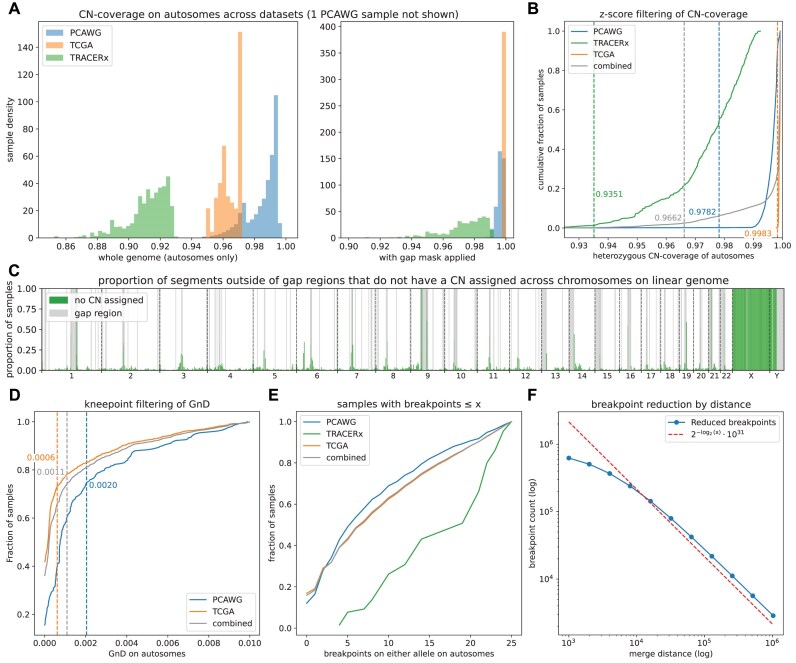
Processing of the PCAWG, TRACERx, and TCGA datasets. (A) Histograms of heterozygous CN-coverage before and after gap region filtering. Note that the PCAWG and TCGA datasets have almost full CN-coverage after filtering. In contrast, while TRACERx shows a major shift, there are still substantial portions missing. (B) Cumulative distribution of samples by heterozygous CN-coverage, with the threshold for filtering given by the *z*-score. The position of the threshold is much higher for the combined dataset compared to the individual ones. (C) Distribution of the missing values in the TRACERx dataset along the linear genome (X and Y are not present in the data). Data are mostly missing in regions close to the centromeres and telomeres, particularly for chromosomes 1 and 9. (D) Cumulative distribution of GnD for a subset of samples below 1%. TRACERx is not shown as none of the samples has hemizygosity below this value. Note the clear slope change around 0.1%, also detected by our knee-point algorithm. (E) Cumulative distribution of breakpoint counts for a subset of samples with less than or equal to 25 breakpoints. The curve is almost linear for all datasets, demonstrating that there is no clear cutoff value in this region. (F) The result of breakpoint reduction using 11 log-distributed merge distances between 1 Kb and 1 Mb. Note that the relationship is proportional—doubling the distance leads to halving the number of resulting segments, as shown by the hyperbolic curve.

We started by imputing any missing data and calculated the sample features (see Supplementary Fig. S4 for complete results). Since SCNA profiles for sex chromosomes were not available in the TRACERx cohort, all sex chromosomes were removed from further analysis. Before region exclusion, the SCNA profiles covered on average 98.47%, 96.39%, and 91.18% for PCAWG, TCGA, and TRACERx, respectively (Fig. 2A). When using the UCSC gap regions for exclusion, the CN-coverage rose to 99.62%, 99.89%, and 97.38%. The gap regions of hg19 on autosomes sum to 19.65 Mb, which is 6.82% of the total genome. For TCGA and PCAWG, virtually all the missing segments fell into these gap regions. In TRACERx, there are regions missing also outside these gap regions, but mostly on their boundaries (Fig. 2C). This was likely due to the sequencing method: PCAWG data have been sourced using WGS, whereas TCGA combines multiple data sources.

Next, samples with low CN-coverage were removed using the *z*-score–based outlier detection (Methods). Thresholds were calculated for each of the datasets separately as well as using the combined dataset of all samples (Fig. 2B). For the individual samples, there was only a small set of outliers: 3, 16, and 19 for the thresholds of 97.82% for PCAWG, 99.83% for TCGA, and 93.51% for TRACERx, respectively. However, when the combined dataset was used, 352 samples were below the detected threshold of 96.62%, stemming from the fact that the CN-coverage distribution of TRACERx significantly differs from the other two. In this case, filtering each set separately leads to significantly lower removal rate. Additionally, 1 sample in the PCAWG dataset, SP107557, had CN-coverage of only 57.67% and presumably should have been blacklisted in the original dataset.

We also removed samples with few or no copy number alterations. Other authors have used the number of breakpoints [9] as evidence for SCNAs, but we did not observe a clear knee-point in the data (Fig. 2E), and any threshold would therefore be arbitrary. Instead, we used the knee-point detection algorithm on the GnD statistic for samples below 1% GnD to determine the following cutoffs (Fig. 2D): 0.06% for TCGA (745 samples removed) and 0.2% for PCAWG (211 samples removed). For TRACERx, all samples were retained. The filtering process then leads to the final filtered sample set of 12,901 samples (see Supplementary Fig. S5 for full sample distribution).

We next evaluated the effects of breakpoint merging. Without any merging, the whole filtered dataset has 826,910 unique breakpoints (i.e., 1 breakpoint per 3.7 Kb on average). We explored different merge distances from 1 Kb to 1 Mb, leading to reductions between 24.39% and 99.65% (Fig. 2F), and selected 1-Mb, 500-Kb, and 250-Kb distances, leading to 2,797, 5,569, and 10,797 autosomal segments, respectively. To compute all combinations of segmentation strategy and datasets efficiently, we made use of CNSistent’s internal parallelization strategy. Runtime decreased in a near-linear fashion with the number of compute cores available (Supplementary Fig. S6). All segmentation configurations are listed in Supplementary Table S2.

### Evaluating segmentation strategies on a cancer classification task

We next set out to explore the effects of different segmentation strategies on the cancer classification task (see Methods). We processed the data using the following segmentation strategies: (i) fixed-size segments of 20 Mb, 10 Mb, 5 Mb, 3 Mb, 2 Mb, 1 Mb, 500 Kb, 250 Kb, and 100 Kb; (ii) whole chromosomes and chromosome arms; (iii) gene-level CN values based on the ENSEMBL and COSMIC gene sets; and (iv) breakpoint merging using distance thresholds of 1 Mb, 500 Kb, and 250 Kb. The segment sizes roughly cover the ranges used by other authors for feature discovery [9].

**Figure 3:**
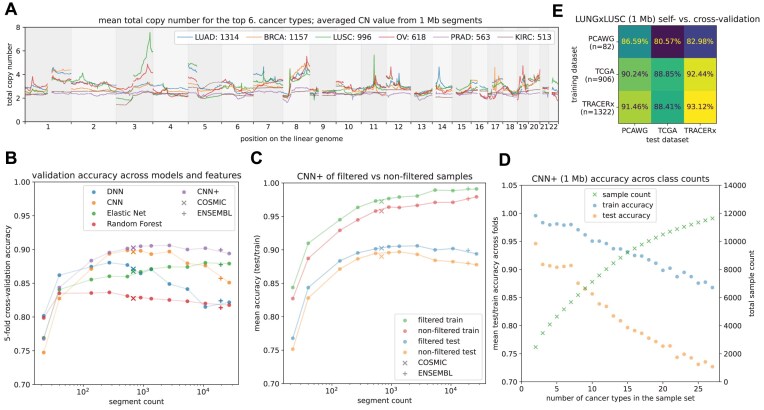
Evaluation of multiclass prediction task. (A) Averaged SCNA profiles of the 6 cancer classes considered for classification. (B) Validation accuracy of the different models tested across increasing granularity of segmentation. DNN and RF quickly reach maximum accuracy and degrade with an increasing number of segments, while the CNN and CNN+ architectures increase until ∼1,000 segments, and ENet even increases monotonously. Results of training on gene-based CNs for each model are displayed using crosses (the number of genes then gives the number of segments). (C) Comparison of training on filtered and unfiltered data. We see that both the train and test accuracy improve after filtering. Additionally, we see that the training accuracy increases almost monotonously, while the validation degrades after ∼1,000 segments, likely pointing to overfitting on smaller segments. (D) Results of classification across 2 to 27 classes on 2-Mb segments. We see nearly linear degradation of both training and testing accuracy, but even for 27 classes, the accuracy is still over 70%. (E) The NSCLC classification task using 2-Mb segments. On the diagonal, the models are scored using 5-fold cross-validation on each individual dataset. The remaining values show results of training on 1 full dataset (row) and validating on another full dataset (column). We can see that in particular, training on bigger sets of TCGA and TRACERx yields better results on PCAWG than training on self.

To our knowledge, the best result to date on the cancer classification task has been reported on the classification of the top 6 cancer types in the dataset in Attique et al. [18], with up to 92% test accuracy on the best model. Using our combined dataset, the selection of the top 6 classes resulted in a set of 5,172 samples with the following class labels: lung adenocarcinoma (LUAD, *n* = 1,314), breast invasive carcinoma (BRCA, *n* = 1,157), lung squamous cell carcinoma (LUSC, *n* = 996), ovarian cancer (OV, *n* = 618), prostate adenocarcinoma (PRAD, *n* = 563), and kidney renal cell carcinoma (KIRC, *n* = 513), with their mean profiles displayed in Fig. 3A.

We only considered segments on autosomes as the sex of the patient acts as a confounder, particularly for BRCA, OV, and PRAD. To evaluate any possible confounding effect of age, we compared the number of breakpoints to the age of the patients across the cohort (*n* = 12,009) and found it to have only a minor effect (*r* = 0.11, Supplementary Fig. S7). Similarly, age alone was a poor predictor of cancer type, achieving a mean test accuracy on a class-balanced one-versus-all linear classifier of 59.03% and a validation accuracy for the multiclass linear classifier of 31.78%.

We compared the DNN3 and CNN architectures of Attique et al. [18], RF, ENet, and our extension of the CNN architecture—CNN+ (Fig. 3B)*—*across decreasing segment sizes. On whole chromosomes, the highest performing models reached a validation accuracy of ∼80%, and considering the arms separately, validation accuracy reached almost ∼86% on the DNN architecture. Increasing the resolution improved accuracy of the 2 convolutional models, which peaked in the region of around 1,000 segments. The best validation (mean 5-fold) accuracy of 90.60% was achieved with the CNN+ at 1-Mb segments, with full the confusion matrix given in Supplementary Fig. S8. We used the 1-Mb segments for the subsequent tasks, but sizes from 5 Mb to 250 Kb all had validation accuracy above 90%, and we would therefore consider all of them to be suitable for any further analyses.

The RF and DNN models, however, peaked around 200 segments, and increasing the resolution further decreased the validation performance, likely due to overfitting. The only architecture that improved monotonously was ENet, where the penalty regularization seemed to prevent overfitting, but from 20 Mb onward, it always underperformed compared to the CNN+. Comparing the full segmentation with the COSMIC and ENSEMBL gene sets, we saw that taking only the CNs for genes performs equivalently to creating a segmentation with a similar number of features. Breakpoint merging performed comparably to bins of the same size; for example, a 500-Kb merge window showed an accuracy of 90.09%, while 500-Kb segments showed an accuracy of 90.0%. Similarly, considering different aggregation strategies for COSMIC and ENSEMBL has not affected the results significantly. For COSMIC, the results were as follows: Min: 90.94% Mean: 90.25%, Max: 90.05%. For ENSEMBL: Min: 87.44%, Mean: 89.92%, Max: 89.54%. Validation on a hold-out set or cross-validation has not been conducted by the authors, so we only performed our comparisons on the test accuracies. The best test accuracy (maximum 5-fold) was 92.42% on 1-Mb segments with the CNN+ mode, slightly above the best test accuracy of Attique et al. [18] (92%). All the deep learning models trained within 100 seconds on a desktop GPU. Full training times are shown in Supplementary Fig. S9. Full training and test results are given in the Supplementary Table S3.

To evaluate the results of filtering, we compared the results on filtered (5,161 samples) and unfiltered (5,257 samples) on the CNN+ model (Fig. 3C). We saw that both training and testing accuracy was consistently better in the filtered dataset. The average test score improvement was 1.28%. Additionally, we were interested in how the CNN+ performs for different numbers of classes. We limited ourselves to classes with at least 100 samples; this yielded 27 classes (Supplementary Fig. S5). In Fig. 3D, it can be seen that the accuracy is quite high for all the cases and decreases in almost a linear fashion. In the easiest binary classification task, we saw 94.6% validation accuracy, while the 27-class task reached 72.69%.

To demonstrate the potential of integration using CNSistent across different datasets, we used the NSCLC classification task, training the models on one dataset and validating on another (Fig. 3E). We see that the accuracies of models trained on a different dataset match or sometimes even outperform models trained and validated on the same dataset, with up to 91.46% accuracy for the TRACERx model applied to PCAWG. We also see that compared to self-training, the models trained on bigger sets (TRACERx, TCGA) outperform self-training on the small PCAWG model. Likewise, training on TRACERx slightly outperforms self-training on TCGA. The 5-fold cross-validation accuracy on the combined dataset was 92.73%, considerably improving on the previous result of 84% in Qui et al. [19]. When training the models individually, we obtain only 91.21% mean validation accuracy, showing that combining the datasets leads to a 1.52% improvement.

### Identifying commonly altered regions and outliers

**Figure 4:**
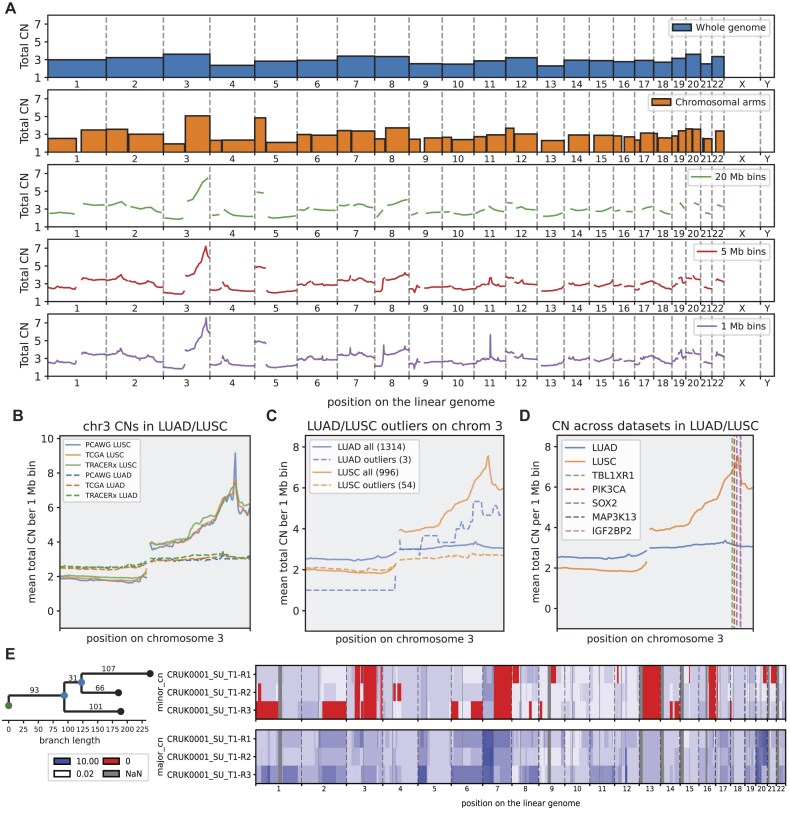
Analyses of LUAD/LUSC CN profiles. Except for the phylogenetic tree, all plots are produced using CNSistent segment plotting functions. (A) The mean CN profiles of the LUSC samples across different segmentations. For each segmentation, the segments with the 3 highest positive (peaks) and highest negative (valleys) peak scores are shown. (B) The 2-Mb CN profiles between different cohorts for both LUSC and LUAD, showing strongly correlated patterns of selection between the cohorts of the same cancer type. (C) Mean CN profile for LUAD and LUSC with the positions of the top 5 genes with the most significant changes in copy number. (D) Mean CN profiles of both LUAD and LUSC compared to the outlier samples (samples with high NMD in their own type and low NMD in the other type). (E) Example of CN heatmap for major and minor CN values in 3 regions of a single tumor together with inferred phylogeny.

Lastly, we demonstrate our segment-derived metrics on the LUAD-LUSC sample set. First, we investigated how the identification of recurrently altered genomic regions is influenced by the segmentation strategies using our simple peak detection algorithm (Methods). We found that the regions with the highest and lowest peak scores differ between segmentation sizes (Fig. 4A), with chr3 being detected mostly in big segments due to a wide slope on the q-arm, while chr8 is mostly detected in middle sizes due to focal amplification at the end of the p-arm, and on chr11, there is very narrow peak that becomes most prominent in smaller segmentations.

Next, we investigated all CN profiles for outliers (Methods). The highest NMD between LUAD and LUSC samples is on chromosome 3, where we can see that LUSC has a distinctive, wide peak on chr 3q (Fig. 4B), while LUAD is mostly neutral. This pattern is extremely well correlated across cohorts. We then calculated the outlier score between LUAD and LUSC and used the knee-point detection to find an outlier threshold (Supplementary Fig. S10), finding 3 LUAD samples with a LUSC-like pattern (amplification of SOX2) and 54 LUSC samples with a neutral LUAD-like pattern (Fig. 4C). We also observed that most of these samples (58.75%) came from the TCGA dataset, while the TRACERx dataset had the least outliers (15.79%).

To systematically determine which genes differ significantly in their CNs between LUAD and LUSC, we conducted a Mann–Whitney *U* test with Benjamini–Hochberg correction on the mean CNs of the COSMIC genes. Out of 722 genes, 599 had an adjusted *P* value below 0.05. The top 5 genes with the most significant changes in copy number were all on the q-arm of chr3 (Fig. 4D). All these had an adjusted *P* value below ${10^{ - 169}}$, with SOX2 [22] being the most significant at $p \approx {10^{ - 187}}$. The SOX2 gene also had the highest mean CN of 7.56.

Lastly, to validate CNSistent segments outside of the tool, we used the 1-Mb segments for phylogeny reconstruction. For this, we used MEDICC2 [6] and applied it to the first patient in the TRACERx dataset with 3 regions. A CNSistent-produced bar plot of the major and minor CNs and a MEDICC phylogenetic tree are shown in Fig. 4E.

## Discussion

We have introduced CNSistent, a new Python-based library for processing and exploratory data analysis of SCNA profiles and applied it to the PCAWG, TCGA, and TRACERx datasets. The main goal of CNSistent is to provide the user with tools for easy data processing, so that SCNA profiles can be jointly used for downstream analysis. There are tools available that call CNs from various sequencing data and provide related visualizations in Python—for example, CNVKit [23] or Segmentum [24], with many more outside Python, with comparison studies done (e.g., by Masood et al. [25]). On the analysis side, there are many well-known tools for detecting regions of interest, particularly GISTIC [26] and BISCUT [27], which take SCNA profiles and combine them, but this is done internally by the tool and not accessible or controllable by the user. To the best of our knowledge, the only tool for integrative analysis of SCNA profiles is the web-based CNApp [14], which shares some of the functionality with CNSistent, particularly resegmentation and calculation of profile statistics. However, CNApp is designed for analysis within a web dashboard, while CNSistent serves as a tool for the integration of data before application of downstream tools. We did not fully compare the tooling to CNApp as the hosting was not available at the time of writing.

Using the filtered and combined datasets, we compared several segmentation methods in providing features for a cancer-type classification task. We observed that the relationship between segmentation size and model accuracy is highly model dependent: the RF model quickly started to overfit, while the ENet model improved near-linearly with the number of segments. In our best-performing model, segmentations within the region of 5 Mb to 500 Kb performed quite equivalently and also matched the results obtained when classifying based on the hand-picked list of COSMIC [15] cancer genes. We adapted the CNN and DNN3 models originally introduced in Attique et al. [18] and showed superior performance. The comparison is, however, limited, since the original model parameters, source code, and source dataset were not available at the time of writing this article. All models and the input dataset used in this study are available online (see Data Availability section). The main purpose of the classification task in this work was to evaluate different segmentation sizes. We therefore assume that better-performing models could still be developed by fine-tuning for a particular segmentation or cancer type.

To investigate whether the results are consistent between cohorts, we compared classification between NSCLC cancers in all 3 datasets and saw that the per-sample accuracy improved by combining these 3 studies when compared to classification of each of them separately. We saw that models trained on one dataset can be successfully applied to classify another, sometimes even outperforming the source dataset, demonstrating that the classifier generalizes well. We also saw that our model trained on the joint dataset had a better accuracy than the average of the individual models trained on the individual dataset. On the joint dataset, our model also considerably outperformed the previous result of Qiu et al. [19]. We therefore conclude that it is worthwhile to aim to integrate datasets from heterogeneous sources.

To show the utility of sample integration in analysis, we investigated the NSCLC samples using statistical methods. We identified chromosome 3 as the region of interest, particularly in the context of LUSC, with a wide peak in the location of the SOX2 gene, a well-known actor in LUSC [28]. Using gene-based segments, we conducted a statistical test to find the most differently altered genes between LUSC and LUAD, which are likewise all located on chromosome 3. Additionally, we used the NMD score to detect outlier samples, with our detected outliers showing the selection pattern of the other cancer, possibly hinting at either mislabeling or co-occurrence of both cancers in the outlier samples [29]. Arguably, these results primarily demonstrate the applicability of our method and warrant further detailed investigations. Future work might also focus on developing methods for within-sample comparison of segments, as well as between-sample and between-type distance calculations.

## Availability of Source Code and Requirements

- Project name: CNSistent
- Project homepage: https://bitbucket.org/schwarzlab/cnsistent
- Operating system(s): Platform independent
- Programming language: Python
- Other requirements: Python 3.8 or higher
- License: MIT
- RRID: SCR_027025
- Bio.tools ID: cnsistent

DOME Annotations have been deposited in the DOME-ML registry [30]. A Snapshot of the CNSistent Bitbucket is available in Software Heritage [31].

## Additional Files

**Supplementary Fig. S1**. An example of the breakpoint clustering method.

**Supplementary Fig. S2**. An example of the knee-point detection method.

**Supplementary Fig. S3**. A schema of the CNN+ architecture.

**Supplementary Fig. S4**. Cumulative plots of calculated features per dataset.

**Supplementary Fig. S5**. Bar plot of the number of samples per cancer type.

**Supplementary Fig. S6**. Runtime of the individual CNSistent commands across various numbers of cores.

**Supplementary Fig. S7**. Relationship between the number of breakpoints and the age of patients.

**Supplementary Fig. S8**. A confusion matrix in the classification of 6 cancer types.

**Supplementary Fig. S9**. Training time across model types and segment counts.

**Supplementary Fig. S10**. Cumulative plot of samples based on their outlier score.

**Supplementary Table S1**. Sample count per source.

**Supplementary Table S2**. Segmentation configurations used.

**Supplementary Table S3**. Results of training of the individual models across segmentation types.

giaf104_Supplemental_Files

giaf104_Authors_Response_To_Reviewer_Comments_Original_Submission

giaf104_Authors_Response_To_Reviewer_Comments_Revision_1

giaf104_Authors_Response_To_Reviewer_Comments_Revision_2

giaf104_GIGA-D-24-00595_Original_Submission

giaf104_GIGA-D-24-00595_Revision_1

giaf104_GIGA-D-24-00595_Revision_2

giaf104_GIGA-D-24-00595_Revision_3

giaf104_Reviewer_1_Report_Original_Submission Stefano Monti -- 2/9/2025

giaf104_Reviewer_2_Report_Original_Submission Ellen Visscher -- 2/20/2025

giaf104_Reviewer_2_Report_Revision_1 Ellen Visscher -- 6/11/2025

giaf104_Reviewer_3_Report_Original_Submission Sampsa Hautaniemi -- 3/11/2025

giaf104_Reviewer_3_Report_Revision_1 Sampsa Hautaniemi -- 6/28/2025

## Abbreviations

AI: allelic imbalance; BRCA: breast invasive carcinoma; CN: copy number; CNN: convolutional neural network; DNN: deep neural network; ENet: elastic Net; GnD: genome not diploid; ICGC: International Cancer Genome Consortium; KIRC: kidney renal cell carcinoma; LoH: loss of heterozygosity; LUAD: lung adenocarcinoma; LUSC: lung squamous cell carcinoma; NMD: normalized Manhattan distance; NSCLC: non–small cell lung carcinoma; OV: ovarian cancer; PCAWG: Pan-Cancer Analysis of Whole Genomes; PRAD: prostate adenocarcinoma; PS: peak score; RF: random forest; SCNA: somatic copy-number alteration; SNP: single-nucleotide polymorphism; TCGA: The Cancer Genome Atlas; TRACERx: TRAcking Cancer Evolution through therapy (Rx); WES: whole-exome sequencing; WGD: whole-genome doubling; WGS: whole-genome sequencing.

## Data Availability

The preprocessed input data are available at [32]. The data produced by CNSistent are available at [33]. The deep learning code and results are available at [34]. The data have been obtained from the following sources, accessed in December 2023: PCAWG data from the ICGC data portal [12]; TCGA data obtained from the ASCATv3 repository [35]; TRACERx data obtained from Zenodo [13]; COSMIC cancer set obtained from the COSMIC portal [36]; human genome gene set obtained using PyENSEMBL (2023) [16]; and Cytoband and Gap data obtained from the UCSC Genome Browser [37]. For TCGA, the ASCAT team called the CNs by ASCATv3 from SNP arrays, the TRACERx team used ASCATv2 from WES, and the PCAWG consortium published CNs obtained as a consensus of 5 different callers [38] from WGS.
