# Supplementary material for: CNSistent integration and feature extraction from somatic copy number profiles
Source: Gigascience. 2025 Sep 13;14:giaf104. doi: 10.1093/gigascience/giaf104 (PMC12448185; doi:10.1093/gigascience/giaf104)
Supplement: giaf104_Supplemental_Files [file giaf104_supplemental_files.zip › Supplementary Figures CNSistent.docx]

Supplementary Figures
CNSistent integration and feature extraction from somatic copy number profiles

Adam Streck^1,3^, Roland F. Schwarz^1,2,3^

1 Institute for Computational Cancer Biology (ICCB), Center for Integrated Oncology (CIO), Cancer Research Center Cologne Essen (CCCE), Faculty of Medicine and University Hospital Cologne, University of Cologne, Germany

2 Berlin Institute for the Foundations of Learning and Data, Berlin, Germany

3 Berlin Institute for Medical Systems Biology, Max Delbrück Center for Molecular Medicine in the Helmholtz Association, Berlin, Germany


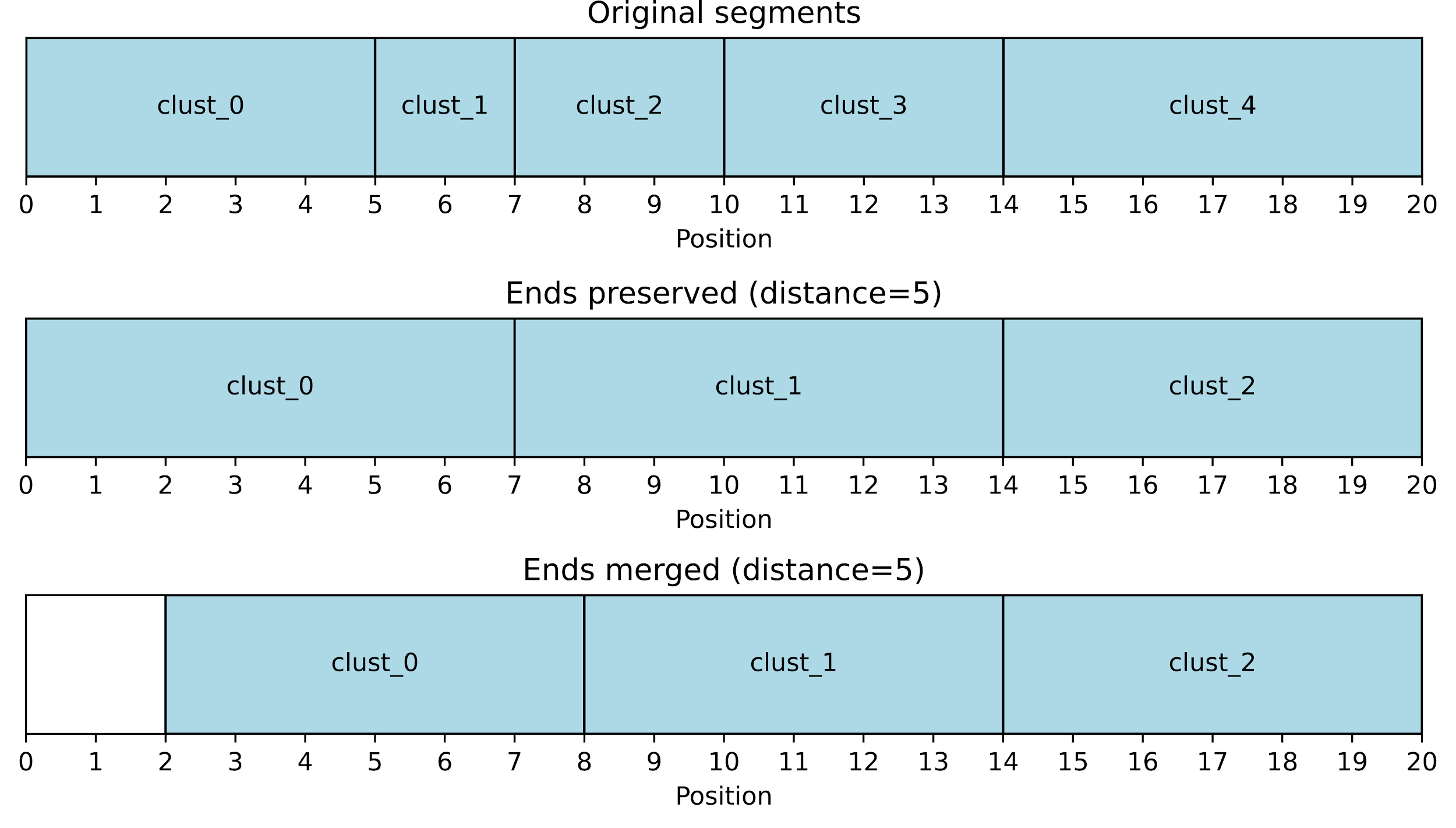


*Supplementary Figure 1: Example of clustering method changing segmentation on a region (top) from 0 to 20, with breakpoints at 5, 7, 10, and 14. The first version with distance 5 preserves the endpoints, therefore 0 and 20 are not considered; 5, 7, and 10 are merged into 10 (rounding down), while the breakpoint at 14 does not get merged on either side–the previous breakpoint at 10 is closer than 5, but has already been merged with 5 and 7. The second version also merges ends, causing 0 and 5 to merge into 2, while 7 and 10 merge into 8.*


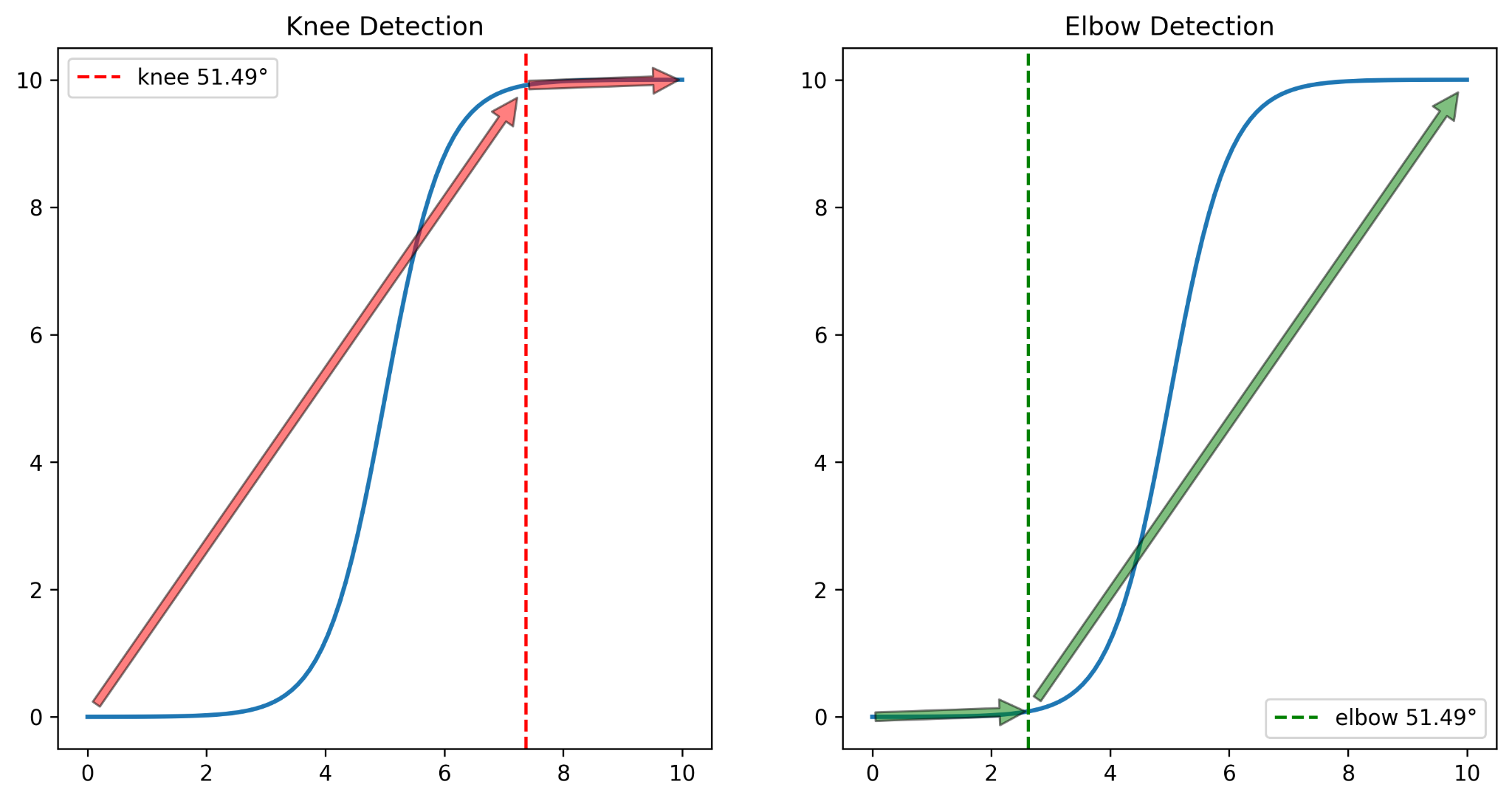


*Supplementary Figure 2: Knee and elbow detection. The angles in the legend are the absolute change in the slope, convex (slope decrease) for the knee, concave (slope increase) for the elbow.*


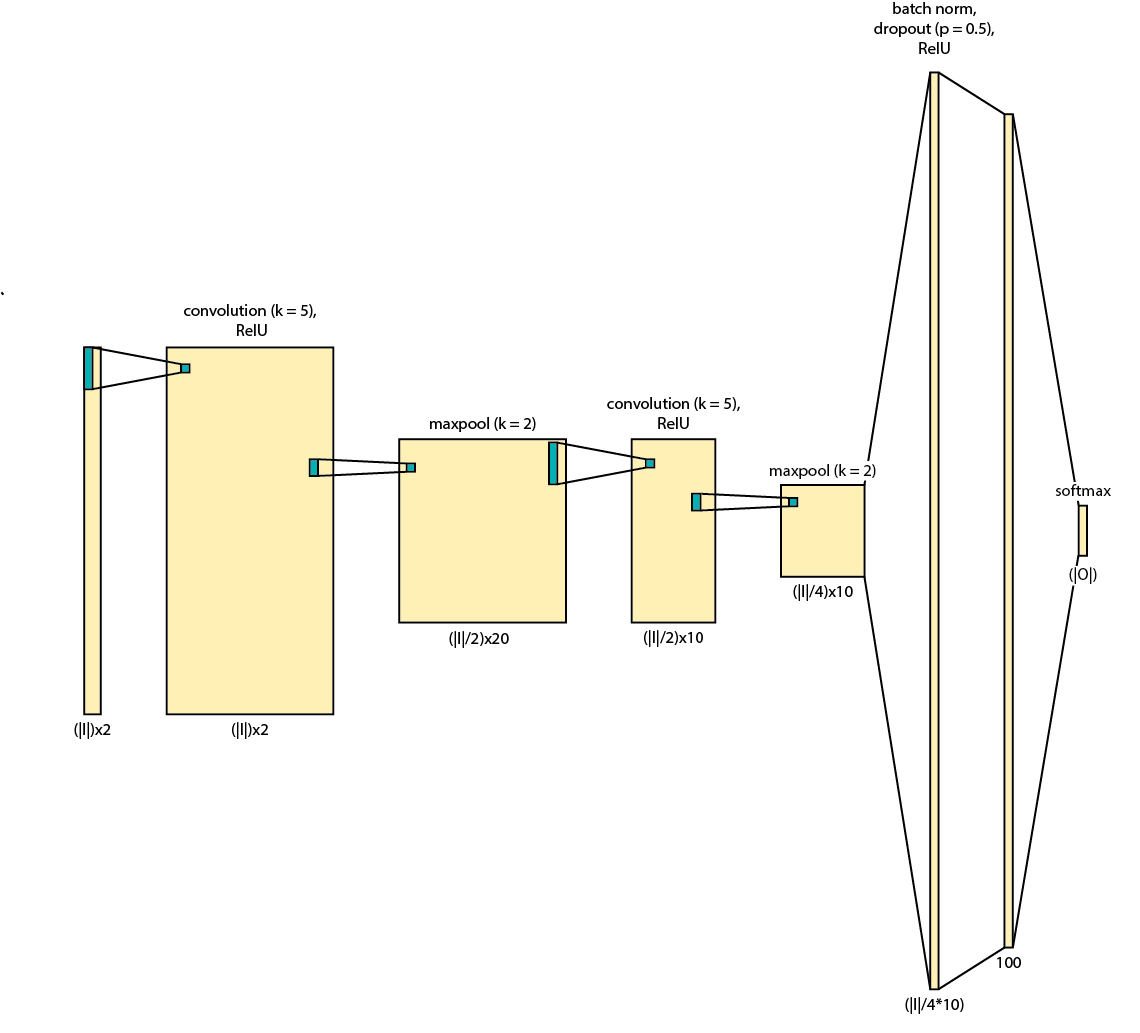


*Supplementary Figure 3: The CNN+ model of auto-scaling 1D convolutional neural network. The input layer I has size |I| and the output layer O has the size |O|, corresponding to the number of classes. The example is visualized for the case of 6-type classification (|O|=6) on filtered chromosome arms (|I|=40).*

*
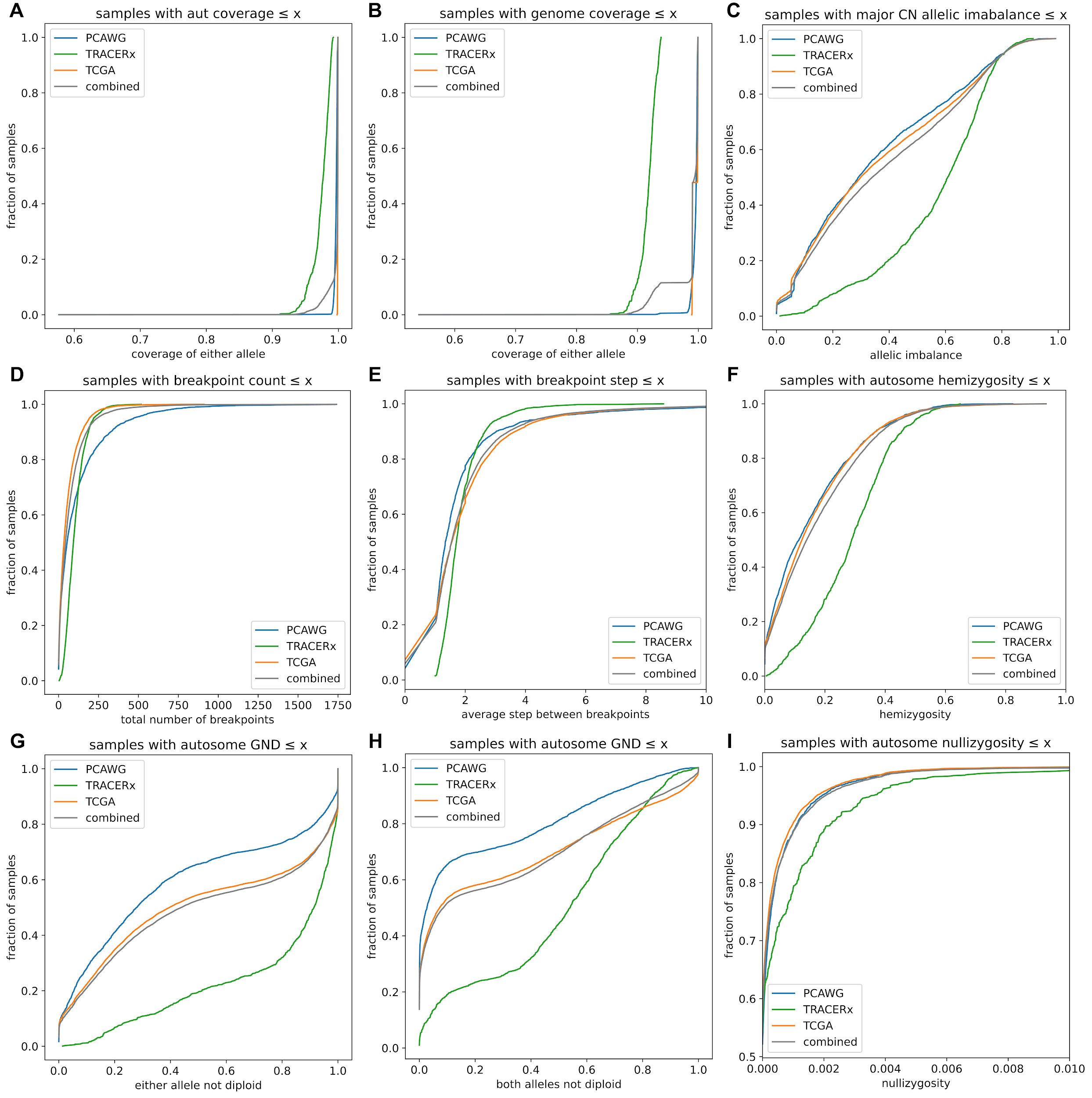
*

*Supplementary Figure 4: Summary features across datasets. Unless otherwise specified, only autosomes are considered. Except for D and E, all features are calculated using gap-mask.* ***A)*** *Coverage for autosomes only,* ***B)*** *Coverage of the whole genome - note the shift on the TRACERx samples which lack sex chromosomes.* ***C)*** *Proportion of the genome with the major allele has higher CN than the minor one.* ***D)*** *Number of breakpoints per sample.* ***E)*** *The average step per breakpoint - there is no value between 0 and 1 as any two breakpoints differ by at least 1.* ***F)*** *Proportion of samples with one of the alleles lost.* ***G)*** *Genome not diploid on either of the alleles in the autosomes.* ***H)*** *Genome not diploid on both of the alleles in the autosomes.* ***I)*** *Proportion of samples where both alleles are 0. The x-axis is scaled for better visibility, as barely more than 1% of samples exhibit nullizygosity.*


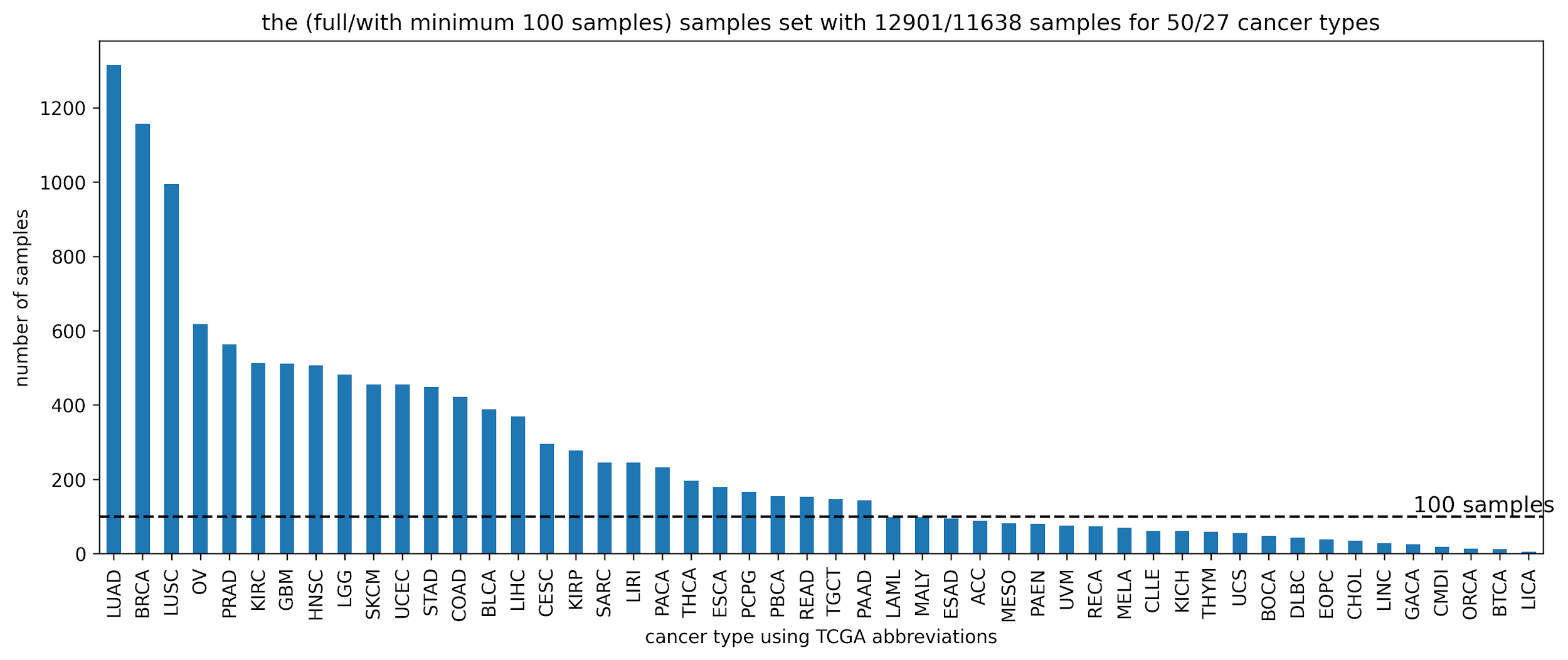


*Supplementary Figure 5: Distribution of samples per cancer type in the final joint dataset. A threshold of 100 samples was set as a minimum for consideration for machine learning, with 27 out of the 50 cancer types within the limit.*


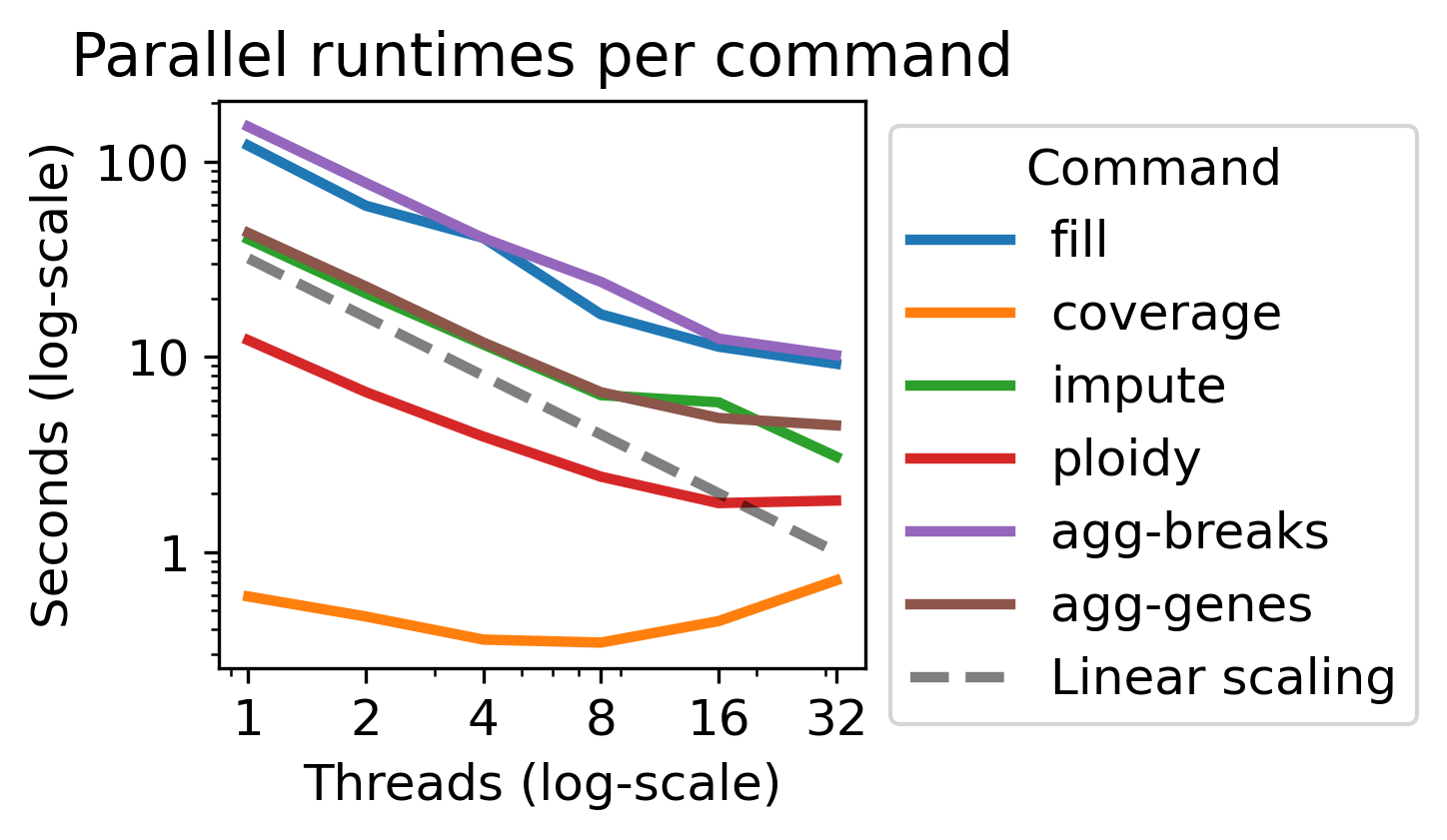


*Supplementary Figure 6: Runtime of the individual CNSistent commands across 1, 2, 4, 8, 16, and 32 threads show near-linear scaling on the PCAWG dataset.*

*
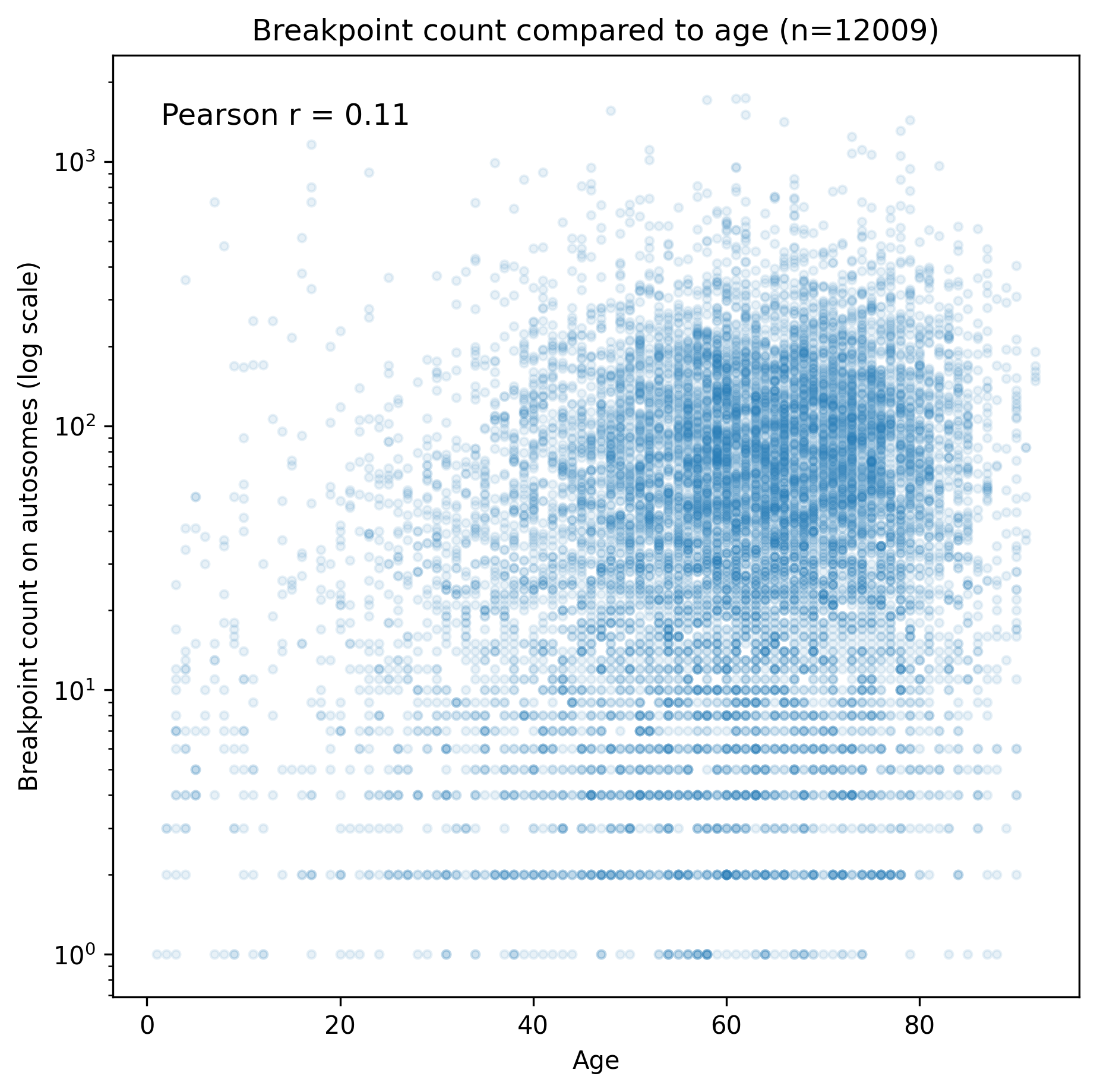
*

*Supplementary Figure 7: Relationship between the number of breakpoints on the imputed dataset and age. Not all samples had age information, therefore only a subset of 12009 samples is displayed. As we can see, there is only a weak association between the variables.*

*~~
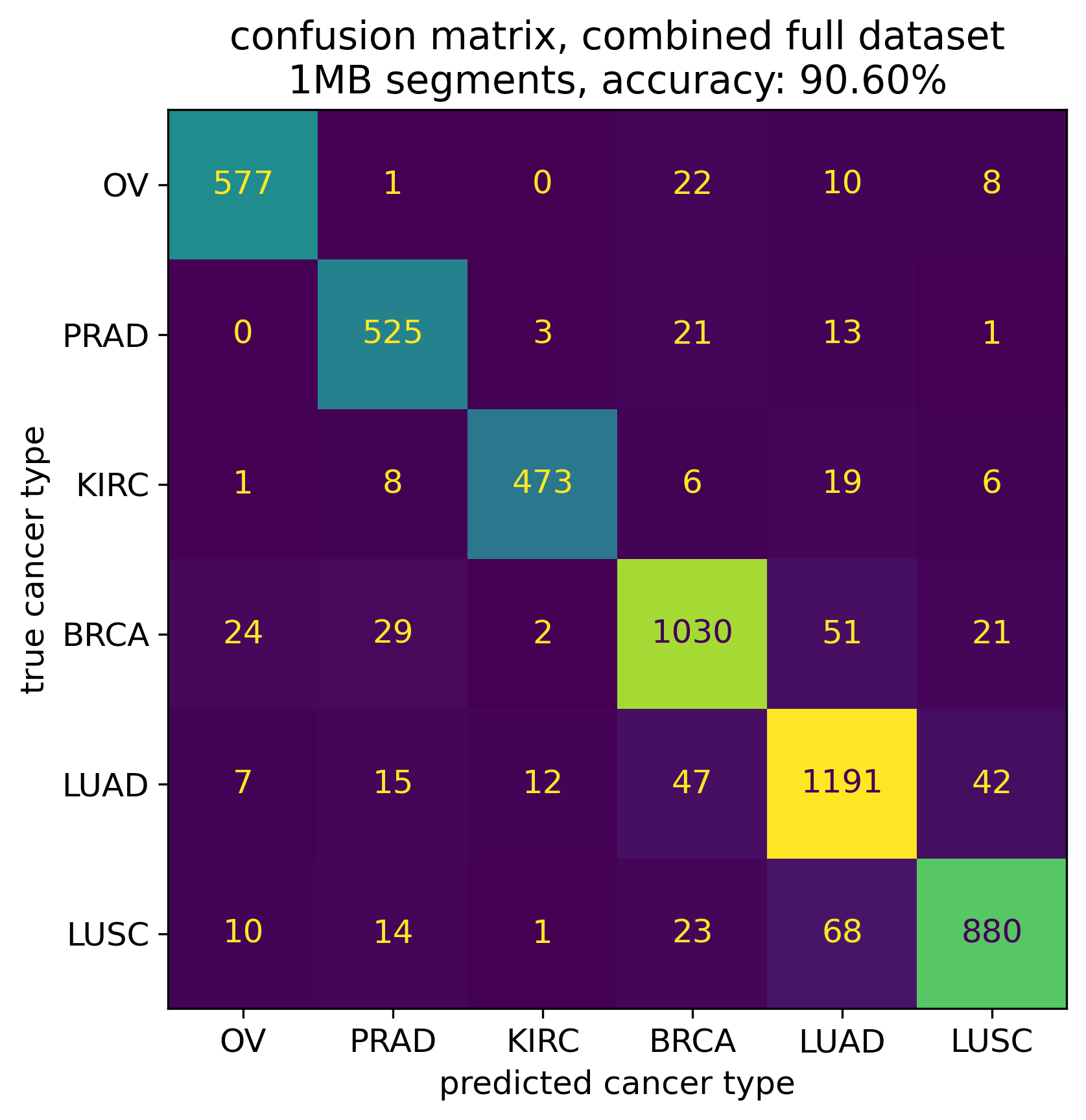
~~Supplementary Figure 8: Confusion matrix for the CNN+ model with 90.60% validation accuracy on top 6 cancer types.*

*
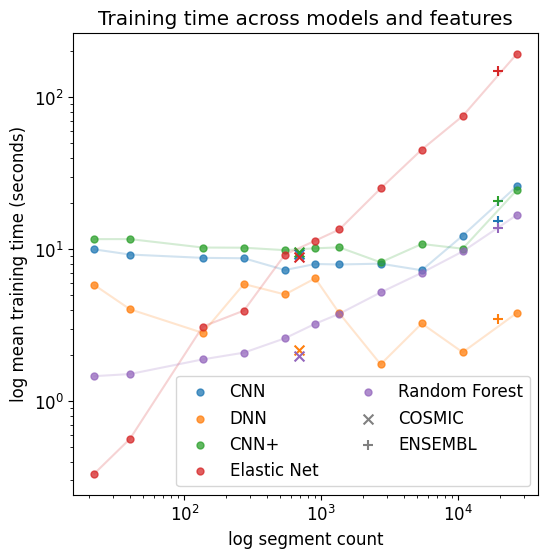
*

*Supplementary Figure 9: Training runtime for different models across segmentation sizes on desktop PC with an NVIDIA RTX 4090 GPU, 128 GB Ram and AMD Ryzen Threadripper 3970X CPU. The flexible convolutional architectures start increasing around 10000 segments, however still finish on average at around 20 seconds. The DNN architecture seems mostly unaffected with training times below 10 seconds. The ENet and RF increase almost linearly with segment count, with the ENet taking around 200 seconds on 100 Kb segments. It should be noted that the neural architectures were trained on GPU whereas the remainder on CPU, making the comparison only illustrative.*

*
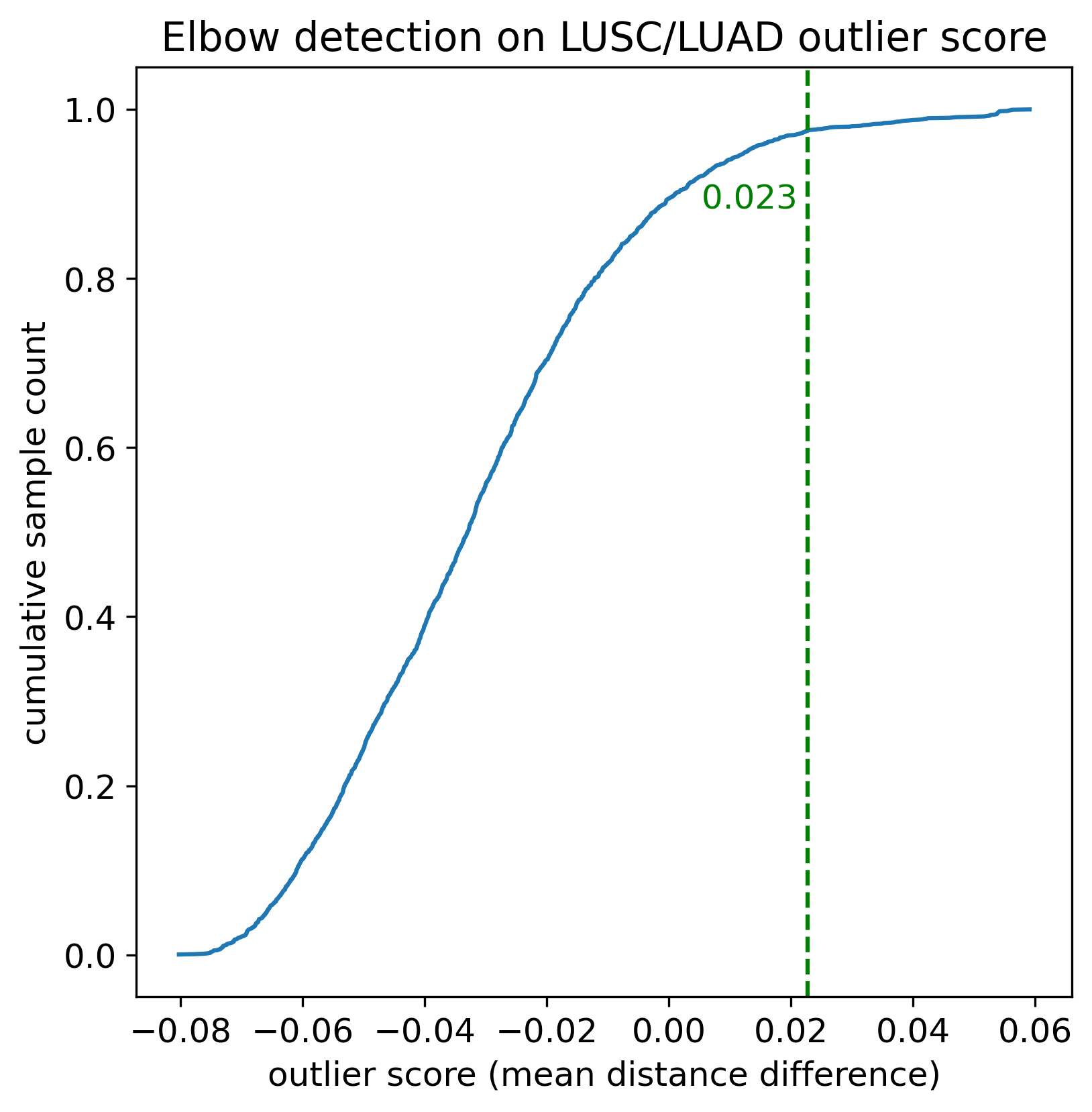
*

*Supplementary Figure 10: Cumulative proportion of samples based on the negative outlier score (the mean distance to other samples of the same type minus the mean distance to the samples of the other type) and detected an elbow point at 0.023, which we then used as a threshold for detection of the outlier samples (above the elbow point).*
