# Supplementary material for: CNSistent integration and feature extraction from somatic copy number profiles
Source: Gigascience. 2025 Sep 13;14:giaf104. doi: 10.1093/gigascience/giaf104 (PMC12448185; doi:10.1093/gigascience/giaf104)
Supplement: giaf104_GIGA-D-24-00595_Revision_1 [file giaf104_giga-d-24-00595_revision_1.pdf]

GigaScience

CNSistent integration and feature extraction from somatic copy number profiles

--Manuscript Draft--

**Manuscript Number:**	GIGA-D-24-00595R1
**Full Title:**	CNSistent integration and feature extraction from somatic copy number profiles
**Article Type:**	Research
**Funding Information:**	German Ministry for Education and Research (01IS18025A, 01IS18037A) — Prof. Dr. Roland F Schwarz<br>Cancer Research Center Cologne Essen (CCCE) — Dr. Adam Streck<br>Bruno und Helene Jöster Foundation (CLONETRAC) — Prof. Dr. Roland F Schwarz
**Abstract:**	The vast majority of cancers exhibit Somatic Copy Number Alterations (SCNAs)—gains and losses of variable regions of DNA. SCNAs play a key role in cancer adaptation through modulation of gene expression, deletion of tumour suppressor genes or amplification of oncogenes. Systematic analysis of SCNAs is now a routine task in both the clinic and research, and can help identify novel cancer genes, improve our understanding of cancer gene regulation and enable us to accurately reconstruct cancer phylogenies. To conduct such analyses however SCNA profiles have to be integrated between samples, patients, and cohorts, an often non-trivial task, for which dedicated toolkits are lacking.<br>To fill this gap, we developed CNSistent, a Python package for imputation, filtering, consistent segmentation, feature extraction, and visualization of cancer copy number profiles from heterogeneous datasets. We demonstrate the utility of CNSistent by applying it to the publicly available TCGA, PCAWG, and TRACERx cohorts. We compare the effect of sample preprocessing and of different segmentation and aggregation strategies on cancer type and subtype classification tasks using various classification models. We also evaluate how well a classifier trained on one cohort generalizes to another. Lastly, we introduce two segment-based peak and outlier scores to investigate relationships between segments, between samples, and between cancer types. Using these scores, we investigate non-small cell lung cancer samples, highlighting that SOX2 amplification is the dominant copy number alteration in lung squamous cell carcinoma and the main distinction to lung adenocarcinoma.
**Corresponding Author:**	Adam Streck, Dr. rer. nat.<br>University Hospital Cologne: Universitatsklinikum Koln<br>Cologne, GERMANY
**Corresponding Author Secondary Information:**	
**Corresponding Author's Institution:**	University Hospital Cologne: Universitatsklinikum Koln
**Corresponding Author's Secondary Institution:**	
**First Author:**	Adam Streck
**First Author Secondary Information:**	
**Order of Authors:**	Adam Streck<br>Roland F Schwarz
**Order of Authors Secondary Information:**	
**Response to Reviewers:**	Below are our itemized responses to reviewers in plain text. For a formatted text with included reference graphics, please refer to the document: "Revision - Responses to Reviewers"<br><br>Reviewer #1:<br>This is a well-written paper that aims to develop a tool that can integrate SCNA from

Powered by Editorial Manager® and ProduXion Manager® from Aries Systems Corporation

large datasets possibly generated using different platforms to identify alteration patterns that are often undetected in smaller data subsets. Authors have used CNN-based method for integrating the data, extracting features and predicting cancer types from SCNA profiles. The tool has the potential to significantly simplify the integration and analysis of large scale SCNA studies. However, some (hopefully addressable) weaknesses are noted:

1. The choice of a classification task as the (only) way to evaluate the proposed method is questioned. I would argue that the most important use of SCNA detection is in support of mechanistic investigations, by identifying novel candidate loci likely to harbor tumor suppressors (copy losses) and oncogenes (copy gains). This type of analysis is hardly mentioned in the manuscript, and it is not clear how well the proposed tool would support it. I surmise it can, but the authors should discuss (and present results about) it.

Response: We agree with the reviewer that a classification task is not the only and arguably not the ideal way of demonstrating the power of CNSistent. CNSistent will prove useful every time CN profiles have to be integrated between samples from the same patient, between patients or between cohorts. Peak detection certainly is one such example. Since GISTIC uses its own internal integration method for CN profiles, it is not ideally combined with CNsistent.

To follow the reviewer's suggestion, we thus implemented our own simple peak detection algorithm, described in the Methods section Peak Detection: "To find regions of interest in the samples, CNSistent provides the peak score (PS), which shows how much each bin differs from its neighbours. Have an aggregated sample S = (s1,,sn) we set the boundary values s0 = s1, sn+1 = sn and calculate i (1,, n): PS(S, i) =(si - si-1) - (si+1-si). This score will be positive for segments higher than their neighbours, negative for those lower and close to zero for segments with monotonous behaviour. We therefore use the PS to detect the highest and lowest values, which show the locations of most abrupt change in CN accumulation. Note that for meaningful calculation this requires that the segments are connected to each other and about the same size."

To illustrate the flexibility of CNSistent we applied this simple algorithm to the LUSC samples with varying segmentation sizes from 500 KB to 20 MB. We observe that the top 3 peaks vary between segment sizes, with chr3 being detected mostly in big segments due to a wide slope on the q-arm, while chr8 is being mostly detected in middle sizes due to amplification at the end of the p-arm (new Fig. 4A).

Based on the reviewer comments, we have also removed the Integrated Gradients method in favour of a statistical test in the Results, which reads: "To systematically determine which genes differ significantly in their CNs between LUAD and LUSC, we conducted a Mann-Whitney U-Test with Benjamini-Hochberg correction on the mean CNs of the COSMIC genes. Out of 722 genes, 599 had adjusted p-value below 0.05. The top 5 most copy number altered genes were all on the q-arm of chr3 (Fig. 4D). All these had the adjusted p-value below 10-169, with SOX230 being the most significant at p10-187. The SOX2 gene also has the highest mean CN of 7.56."

To further illustrate the broad applicability of CNSistent we now provide phylogenetic inference from somatic copy number profiles as an additional example. To this end we leveraged samples from the TRACERx cohort as described together with our phylogenetic inference tool MEDICC2 (cite Kaufmann et al. 2022). Before any phylogenetic analysis can be carried out CN profiles from the same patient have to undergo joint segmentation to make them comparable by an evolutionary model. Figure 4E now shows the CN profiles from TRACERx case CRUK0001 aligned with CNSistent as well as the phylogenetic tree inferred by MEDICC2.

2. If we were to focus on the task of recurrent SCNA detection, then meta-analysis approaches (where separate analyses are performed on each of the datasets, and only the results are integrated) would need to be considered as an alternative to the approach here proposed (e.g., application of GISTIC to each of PCAWG, TCGA, TRACERx separately, followed by meta-analysis integration of the results). I am not saying meta-analysis would be superior, but the authors should discuss it, and possibly

evaluate it.

Response: We agree with the reviewer that meta-analysis techniques can be an alternative to CNSistent in some situations. For example, simple gene-level aggregations of CN states might also be possible with ad-hoc implementations. However, for larger segment sizes or as soon as whole-genome CN profiles are considered, technical differences between samples, patients or cohorts, for example with respect to missing data or blacklisted regions will require algorithmic design decisions that are not trivial. CNSistent provides such tools to enable reproducible processing of large cohorts in a unified manner.

Unfortunately, a direct comparison with GISTIC is not possible, since GISTIC uses its own internal integration strategy. However, as described in response to this reviewer's comment #1, we have implemented our own simple peak detection algorithm to illustrate the use of CNSistent for this purpose and would refer the reviewer to the above comment for details. We have also added a paragraph in the Discussion to clarify this point. It now reads:

"On the analysis side, there are many well known tools for detection of regions of interest, in particular GISTIC34 and BISCUT35, which take SCNA profiles and combine them, however, this is done internally by the tool and not accessible or controllable by the user."

To demonstrate the increase in power in combining many patients or cohorts we show on the task of NSCLC classification that training on TRACERx and applying the results to PCAWG provides better results than training on PCAWG itself. To make this clearer in text, we have changed the Methods to the following:

"To demonstrate the potential of integration using CNSistent across different datasets. For the binary lung cancer classification task we have trained the models on one dataset and tested on another (Fig. 3E). We see that the results transfer well, with up to 93.90% accuracy for the TRACERx model applied to PCAWG. We also see that compared to self-training, the models trained on bigger sets (TRACERx, TCGA) outperform self-training on the small PCAWG model. Likewise, training on TRACERx slightly outperforms self-training on TCGA. The 5-fold cross-validation accuracy on the combined dataset was 92.12%, considerably improving on the previous result of 84% in Qui et al.(Qiu et al. 2017)."

3. The reported metrics to quantify the quality of the integration are insufficient to assess the results. There is some lack of clarity about the classification accuracy results reported, since it is not clear whether all the components of the model building were adequately brought into the cross-validation (or train/test) loop. More specifically, when reporting the accuracy of the cancer type classification, it is reported that 1 megabase segmentation yields the best results. It is not clear if this size selection was performed within the train set only (and/or within the CV loop) or across the entire dataset. If the latter, this may significantly affect the accuracy results, which could not be deemed (unbiased) "test set" results. This should be clarified, and if the segment size selection was indeed performed outside the train/test split, accuracy measures should be computed again by performing the segment size selection properly (which of course it would mean a potentially different size would be selected for each of the folds).

Response: We thank the reviewer for raising this point, which we hopefully can clarify. We here aimed to compare the different segmentation strategies, rather than to select the best model. The split into the 5 folds is the same for each segmentation strategy and the accuracy has been averaged across the 5 folds in order to best demonstrate how well a method would perform on such a segmentation. We are aware that using this method makes the nomenclature somewhat confusing, since the validation accuracy is the mean of the test accuracies. We have aimed to address this by including a new paragraph in the Machine learning subsection:

"To obtain scores of individual models on the individual datasets, we use 5-fold cross validation, i.e. we split each dataset into 5 groups and always withheld one while training on the other 4. The validation accuracy for each model/size combination is

Powered by Editorial Manager® and ProduXion Manager® from Aries Systems Corporation

then the mean of the 5 different splits (Yadav and Shukla 2016)."

We use this validation technique rather than train-test-validation split because i) the dataset is quite small, ii) we mostly aim to compare between different options and averaging training results prevents possibility of favorable selection of validation set for a particular method.

The segmentation strategies are pre-selected and tested independently, i.e. there's no segment size selection during the train/test process, we just report the results of all the options. We tried to make the segment size selection process clearer with the following paragraph in Results:

"We next set out to explore the effects of different segmentation strategies on the cancer classification task (see Methods). We processed the data using the following segmentation strategies: (i) fixed-size segments of 20 Mb, 10Mb, 5 Mb, 3 Mb, 2 Mb, 1 Mb, 500 Kb, 250 Kb, 100 Kb size; (ii) Whole chromosomes, chromosome arms; (iii) Gene-level CN values based on the ENSEMBL and COSMIC gene sets; and (iv) breakpoint merging using distance thresholds of 1 Mb, 500 Kb, and 250 Kb. The segment sizes roughly cover the ranges used by other authors for feature discovery(Drews et al. 2022)"

4. Comparisons with other methods: The authors only compare their method to random forest (RF). Related to the previous point: I presume the RF model used the segment size that was optimized for the CNN model (i.e., 1Mb). If this is the case, it would be an unfair comparison, since RF might favor a different size. Also, additional classifiers should be evaluated (e.g., Elastic Net, SVM, etc.).

Response: We have now calculated the performance of the RF classifier across all segment sizes and added an additional linear classifier with Elastic Net regularization for comparison. We decided against inclusion of a SVM as the performance of this kernel-based classifier would strongly depend on the kernel and hyperparameters used.

While our original neural network was very close to the architecture used by Attique et al., it included an additional linear layer. We therefore now reconstructed the DNN3 and CNN of Attique et al. to the best of our ability based on their manuscript and included our model as an additional one, called CNN+.

The corresponding text in the results section reads:

"We compared the DNN3 and CNN architectures of Attique et al., Random Forest, Elastic Net, and our extension of the CNN architecture–CNN+ (Fig. 3B)–across decreasing segment sizes. On whole chromosomes the most performing models reached a validation accuracy of ~80% and considering the arms separately reached almost ~86% on the DNN architecture. Increasing the resolution improves accuracy of the two convolutional models, which peak in the region of around 1000 segments. The best validation (mean of 5 folds) accuracy of 90.60% was achieved with the CNN+ at 1 Mb segments, which we used for the subsequent tasks, however sizes from 5 Mb to 250 Kb all had validation accuracy above 90%, and we would therefore consider all of them to be suitable for any further analyses.

The RF and DNN models however peak around 200 segments and increasing the resolution further decreases the validation performance, likely due to overfitting. The only architecture that improved monotonously was ENet, where the penalty regularization seemed to prevent overfitting, however from 20 Mb onwards it always underperforms compared to the CNN+. Comparing the full segmentation with the COSMIC and ENSEMBL gene sets we saw that taking only the CNs for genes performs equivalently to creating a segmentation with a similar number of features."

5. There is no sufficient discussion of existing tools/methods. This should be corrected (see also my comment about meta-analysis approaches).

Response: To better situate CNSistent in the field and to provide a better overview of existing alternative tools, we have added the following paragraph to the Discussion:

There are tools available that call CNs from various sequencing data and provide related visualizations in Python, e.g. CNVKit31 or Segmentum32, with many more outside Python, with comparison studies done e.g. by Masood et al.33. On the analysis side, there are many well known tools for detection of regions of interest, in particular GISTIC34 and BISCUT35, which take SCNA profiles and combine them, however, this is done internally by the tool and not accessible or controllable by the user. To the best of our knowledge the only tool for integrative analysis of SCNA profiles is the web-based CNApp14, which shares some of the functionality with CNSistent, in particular re-segmentation and calculation of profile statistics. However, CNApp is designed for analysis within a web dashboard, while CNSistent serves as a tool for the integration of data before application of downstream tools. We did not fully compare the tooling to CNApp as the hosting was not available at the time of writing.

6. Metadata effects: Age influences the copy number alterations. The authors don't consider age or any other metadata and their implication in the classification task.

Response: We thank the reviewer for pointing this out. To evaluate if age might act as a confounding in our cancer type classification task we investigated the predictive power of age on the cancer types considered. A simple Linear classifier on the top 6 types showed a test accuracy of 0.322, indicating that age alone is not a strong predictor of cancer type and thus is unlikely to confound our classification results.

Unfortunately, we were not able to obtain age values for ~10% of the samples. In the interest of manuscript length, we have decided not to include this analysis in the text but naturally would be happy to do so if the reviewer feels strongly about this.

7. Run time statistics and user requirement: While the authors report runtime curves per command (S Fig 6), it is difficult to translate this to total runtime. It would be useful if runtime for the entire training of a model were reported. Additionally, if available, comparison of run time stats with the established model that they cite would be useful.

Response: We have reproduced two of the existing architectures and compared the times to our model, Random Forest and Elastic Net.

8. IG-based explanation. I found this section sort of perfunctory, not sufficiently justified, and adding little to the manuscript. IG is computationally expensive, and it does not provide any way to statistically quantify the found associations. Simpler methods, such as testing for association between SCNA occurrence and cancer type should be evaluated and compared to.

Response: We originally used IGs as a preliminary exploration of points of interests, but we agree with the sentiment concerning the computational costs. We have now completely removed the IG section and instead replaced it with a simpler section focused on a statistical test for association between cancer type and the prevalence of copy number changes.

Briefly, we applied our Peak Score to 1 Mb segments. This detected the peak in the vicinity of SOX2 on chromosome 3 and the focal amplification at the p-arm of chromosome 11 near the centromere, matching the results of the application of IGs to Segments.

We then conducted Mann-Whitney U-Test on the COSMIC gene sets comparing the LUAD-LUSC sample sets. The set of the 5 most statistically significant differences between the genes and the set of 5 genes with the highest IG score have both on 3 of the genes: SOX2, PIK3CA, and TBL1XR1.

We therefore conclude that these much simpler and more explainable methods suit as a good replacement of our previous IG-based analysis.

For details, please see the updated section: Identifying commonly altered regions and outliers .

9. Model selection: No adequate justification of why they picked CNN for this task

when the referenced paper itself claims the DNN architecture performs better. Not sure but is this because of the varying segment size? Again, this is not clearly stated. https://pmc.ncbi.nlm.nih.gov/articles/PMC9203194/#tab1

Response: We have attempted to reconstruct the models of Attique et al. as faithfully as we could based on the manuscript, however there were limitations, which we summarized at the end of the Discussion:

“Unfortunately, the comparison of our models to the one from Attique et al18 was partially limited by the fact that the authors did neither provide all of the model parameters, nor the model source code, and that the source dataset was no longer available at the time of writing this article.”

In the methods we specify that the Dropout probability and MaxPool kernel size are not declared. Additionally, it was not clear what the layer sizes for DNN5 were. As DNN3 has already been overfitting in our implementation, we have not included the DNN5. Also the declared test accuracies of DNN3 and DNN5 were 91% and 92% respectively, therefore we have not felt that there was a meaningful difference.
Reviewer #2:
The paper introduces a python package for imputation, filtering, segmentation, feature extraction and visualisation of CNA profiles. It explains some of the elements of the package, and then demonstrates how data from multiple cohorts can be processed and combined using the package preprocessing pipeline. The authors then use processed data from 3 different cohorts to perform cancer type prediction using a CNN. From this, they get an interesting result to find a biomarker that differentiates two different lung cancers. Throughout, they show visualisations using their package. The package itself seems well documented and designed to be used. There is some clarification required in the methods section specifically around the CNN training and the models therein. There is also one major question of whether all the preprocessing steps are actually required for the downstream CNN analysis. Overall, however, this is a well written manuscript, providing a useful
software tool for further analysis of CNA data.

Major comments:
- CNN section- how are the segments decided- is it based on all the training data, or just data in a batch?

The segmentation strategies are pre-selected and tested independently, i.e. there’s no segment size selection during the train/test process, we just report the results of all the options. We tried to make the segment size selection process clearer with the following paragraph in Results:

“We next set out to explore the effects of different segmentation strategies on the cancer classification task (see Methods). We processed the data using the following segmentation strategies: (i) fixed-size segments of 20 Mb, 10Mb, 5 Mb, 3 Mb, 2 Mb, 1 Mb, 500 Kb, 250 Kb, 100 Kb size; (ii) Whole chromosomes, chromosome arms; (iii) Gene-level CN values based on the ENSEMBL and COSMIC gene sets; and (iv) breakpoint merging using distance thresholds of 1 Mb, 500 Kb, and 250 Kb. The segment sizes roughly cover the ranges used by other authors for feature discovery(Drews et al. 2022)”

- Throughout the results pertaining to figure 3A-C, you call it test accuracy- to be clear is this is based on your CV hold outs? This should be reworded everywhere to reflect this. As cross validation indicates, this is not a test set and is a validation set- which is also the way you use it.

We thank the reviewer for raising this point, which was also raised by Reviewer 1. In essence, we aimed to compare the different segmentation strategies, rather than to select the best model. The split into the 5 folds is the same for each segmentation strategy and the accuracy has been averaged across the 5 folds in order to best demonstrate how well a method would perform on such a segmentation. We are aware that using this method makes the nomenclature somewhat confusing, since the validation accuracy is the mean of the test accuracies. We have aimed to address this by including a new Methods paragraph in the text as shown below:

Powered by Editorial Manager® and ProduXion Manager® from Aries Systems Corporation

“To obtain scores of individual models on the individual datasets, we use 5-fold cross validation, i.e. we split each dataset into 5 groups and always withheld one while training on the other 4. The validation accuracy for each model/size combination is then the mean of the 5 different splits (Yadav and Shukla 2016).”

- Regarding the above, you have a comment saying: "the best test accuracy without cross-validation was 92.34%". Could you please clarify what you mean by this. Only in the CNN section do you describe your training approach, which does not mention a test or separate validation set.

Response: We hope the validation approach has been made clearer by the above paragraph. The test accuracy of 92.34% comes from the fact that Attique et al. did not conduct validation, therefore we can only compare to their test scores. However, we believe the validation score to be a more informative metric of classification performance and therefore chose to work with that number instead. We have updated the Results to reflect this as follows:

“The best test accuracy (maximum of 5 folds) was 92.42% on 1 Mb segments with the CNN+ model. This is slightly above the best test accuracy of Attique et al. (92%).“

This is further discussed in the Discussion:

“Validation on a hold-out set or cross-validation has not been conducted by the authors, we therefore only performed our comparisons on the test accuracies.“

- It reads slightly unclearly- you have a section called "model transfer", but are you training 3 different models- one per dataset? You only have one figure for training results which suggests one dataset, but then you have this section called model transfer?

Response: We agree with the reviewer that our terminology might have been confusing and have removed the term “transfer” altogether. We have indeed trained 3 different models, each on one dataset and applied it independently to the remaining two, creating 6 combinations, where one dataset is the training set and the other is the validation set. We have then compared these to the results of the 5-fold cross validation on each individual dataset. We have generally scaled this section back in line with the decrease of focus on deep learning and tried to express the process as clearly as possible in the Results:

“To demonstrate the potential of integration using CNSistent across different datasets we used the NSCLC classification task, training the models on one dataset and validating on another (Fig. 3E). We see that the accuracies of models trained on a different dataset match or sometimes even outperform models trained and validated on the same dataset, with up to 91.46% accuracy for the TRACERx model applied to PCAWG. We also see that compared to self-training, the models trained on bigger sets (TRACERx, TCGA) outperform self-training on the small PCAWG model. Likewise, training on TRACERx slightly outperforms self-training on TCGA. The 5-fold cross-validation accuracy on the combined dataset was 92.73%, considerably improving on the previous result of 84% in Qui et al. When training the models individually, we obtain only 91.21% mean validation accuracy, showing that combining the datasets leads to a (1.52%) improvement.”

- Re all the above, please dedicate a small subsection in methods making this clearer. Are there dedicated test sets? If your main results are for aggregated data, then what are you testing on to ensure generalisability? What is the point of training the 3 different models on 3 different datasets? Perhaps it would make more sense to hold one dataset out as your test set. In some ways, that is what the model transfer is showing, but it would be less confusing to clarify that aim instead of suddenly introducing 3 models.

We have now updated the Methods section to accommodate the comments by the Reviewer as indicated in detail in the answers above, to which we would kindly refer you here.

Powered by Editorial Manager® and ProduXion Manager® from Aries Systems Corporation

Regarding the question about the three models, we use this to test whether classifiers can be transferred from one cohort to another, similar to a train/validation split, i.e. we hold out the remaining two cohorts and train only on one cohort with the remaining two used to obtain two validation scores. We hope that this is also clearer from the text quoted above.

- If the CNN architecture is essentially the same as in Attique et. al., the performance is basically the same and they use only CNs a gene locations- how does this demonstrate that the preprocessing from CNSistent is necessary or advantageous for this task? Maybe having a result which combines CN calls naively over gene locations and comparing to this across the aggregate datasets would be a good way of comparing? I.e showing that preproccessing does offer an advantage when combining different datasets together? Also because this is what you argue in your abstract. For this analysis you would have to make sure you also compare across the same samples to differentiate between filtering/other preprocessing steps.

Response: We agree with the reviewer that aggregation or meta-analysis techniques can be an alternative to CNSistent in some situations. For example, simple gene-level aggregations of CN states might also be possible with ad-hoc implementations. However, for larger segment sizes or as soon as whole-genome CN profiles are considered, technical differences between samples, patients or cohorts, for example with respect to missing data or blacklisted regions will require algorithmic design decisions that are not trivial. CNSistent provides such tools to enable reproducible processing of large cohorts in a unified manner.

To demonstrate the increase in power in combining many patients or cohorts we show on the task of NSCLC classification that training on TRACERx and applying the results to PCAWG provides better results than training on PCAWG itself, as discussed in the paragraph above.

Furthermore, we have added a comparison of filtered (pre-processed) and non-filtered (all available) sample sets in Figure 3C, which shows clear and consistent improvement after the filtering process:

- In Figure 3I, you say "notice the similarity of chromosome 3 pattern for the correctly classified LUSC samples (red) and the misclassified ones (orange)". This is confusing because the orange and red are not similar. In fact for this whole section, it seems that figure 3I does not align with what you are saying?

Response: We apologise for this mistake on our part. There was indeed an error in Figure 3. However, in Response to Reviewer 1 comment #8, we have now removed the IG analysis from the paper and replaced it with a statistical association test. This also removes Figure 3I. We reproduce the new Results paragraph and corresponding Figure 4C here for your convenience:

“We then calculated the outlier score between LUAD and LUSC and used the kneepoint detection to find an outlier threshold (Supp. Fig. 8), finding 3 LUAD samples with LUSC-like pattern (amplification of SOX2) and 54 LUSC samples with neutral LUAD-like pattern (Fig. 4C). We also observed that the majority of these samples (58.75%) came from the TCGA dataset, while the TRACERx dataset had the least outliers (15.79%).”

Minor comments/errors:
- Clarification on why CNSistent needs a reference genome if it's dealing with segments? How is this information used- is it just for the known gaps?

Response: The reference genome is needed to know the expected chromosome lengths for the imputation as described in the Segment Imputation subsection “(ii) CNSistent extends the first and last segment of each chromosome to the chromosome boundaries”. CNSistent also provides built-in segmentation based on cytobands (listed as option in Consistent Segmentation subsection), whose locations are likewise taken from the reference.

Powered by Editorial Manager® and ProduXion Manager® from Aries Systems Corporation

- Your caption of Supplementary Figure 1 has a typo about a breakpoint at 16 instead of 14.

Response: Thank you for noticing, we have fixed the typo.

- You do not explain how you use the knee pt to filter (i.e is it samples above/below the knee pt.)

Response: “We used individual thresholds to filter the samples” changed to “We used individual thresholds to remove samples whose values were strictly smaller than the threshold”

- Your CNN graphic is difficult to interpret and non-standard.

Response: We have updated the figure to more closely match the standard visual scheme for CNNs and updated the figure caption:

Supplementary Figure 3: The CNN+ model of auto-scaling 1D convolutional neural network. The input layer I has size |I| and the output layer O has the size |O|, corresponding to the number of classes. The example is visualized for the case of 6-type classification (|O|=6) on filtered chromosome arms (|I|=40).

- CNN section should clarify at the beginning what the input is and what the output is (i.e a prediction that a sample belongs to a particular cancer type) before explaining the architectural details.

Response: We have added the following to the Machine learning subsection to clarify: “In this task, each binned sample as illustrated in Fig. 1 represents one feature vector. The output probability that a sample belongs to each cancer class under consideration.”

- Even though you control for class imbalance, some cancer types are so poorly represented it is unlikely a CNN could learn that, you do kind of mention this in the discussion, but maybe some sort of minimum threshold for inclusion would make sense.

Response: Thank you for the suggestion, we agree with the point and we have hence limited the classification to only classes with at least 100 samples. The supplementary figure has been updated to reflect this:

- For Fig2D you refer to it as GND, but the axes/title says hemizygosity-are these things equivalent? E.g could have 3-3, low hemizygosity but not diploid? Or if it's aggregated across the whole genome its assumed equivalent?

Response: Thank you for spotting the discrepancy. The x-axis label and title were supposed to read GnD, which we have now corrected.

- There is a grammatical error "Runtimes decreased in a near-linearly with the number of compute cores"

Response: Thank you for spotting the mistake, we have corrected it to: “Runtime decreased in a near-linear fashion with the number of compute cores available.”

- You make a comment that "We therefore suspect some TCGA lung cancers might be cases of co-occurring adeno and squamous carcinomas." This is a possibility but given pleiotropy of many phenotypes- it may also be that the biomarker is not always unique to squamous carcinomas.

Response: We have updated our analysis using the outlier detection method where the SOX2 amplification shows a considerably clearer pattern (see below), however it is still a hypothesis and we cannot confirm or reject it from copy numbers alone.

Suggestions/Nice to haves:

- Maybe make it clearer inside the paper what visualisations come with CNSistent. Looking at the software documentation, there's obviously a lot of useful visualisations

Powered by Editorial Manager® and ProduXion Manager® from Aries Systems Corporation

that come with that- and some of them you have used in Figure 3 for e.g.

Response: All of the plots in Figure 4 are now produced by CNSistent. The label also states that explicitly: “Except for the phylogenetic tree, all plots are produced using CNSistent plotting functions.”

- Given there are more total CN callers, maybe good to mention somewhere how CNSistent would work for total CNs only.

Response: We have added the following sentence to the methods: “The processing is the same for both allele-specific and total copy numbers, however some of the statistics are limited in the case of copy numbers, as detailed below.”

- You remove profiles that you say are uninformative, could you not include this and then just show how accuracy correlates with no. of break-pts (for e.g). In some ways one might think that there could be useful information in few alteration profiles- because those alterations might be more upstream/causal.

Response: We hope that the Figure 3C demonstrating the difference between filtered and unfiltered samples demonstrates the benefit of filtering:

We preferred to display this on our filtering results rather than on the breakpoint count as we don’t see breakpoints as we tried to argue for the GnD as an evidence of SCNAs rather than a breakpoint count, as we argued in Fig. 2E and the related text:

“Other authors have used the number of breakpoints9 as evidence for SCNAs, however we have not observed a clear knee-point in the data (Fig. 2E) and any threshold would therefore be arbitrary.”

- The aggregation step could maybe affect downstream analysis. I.e taking the average could introduce CNs that were never called. Even using min/max- this implies a constant copy number in that region, which may lose information- e.g if it is a functional region having two diff CNs across gene might imply non-functionality. Did you explore the effect of aggregation step? Perhaps taking a small enough resolution of segment types would account for this anyway.

Response: We have added a 100 Kb resolution, which is still about 30-times smaller than what a full minimum consistent segmentation would be, however even there we see a trend of flattening training accuracy and decreasing test accuracy, therefore we presume smaller segmentations would not help.

Additionally we compared the Min/Mean/Max strategies for COSMIC and ENSEMBL. The following paragraph has been added to the results:

“Similarly, considering different aggregation strategies for COSMIC and ENSEMBL has not affected the results significantly: for COSMIC the results were Min: 90.94% Mean: 90.25%, Max: 90.05%. For ENSEMBL: Min: 87.44%, Mean: 89.92%, Max: 89.54%.”
Reviewer #3:
Streck and Schwarz present a method, CNSintent, for consistent segmentation of copy-number data. The utility of the tool is demonstrated using three large cancer cohorts and a neural network classifier built upon the consistently segmented data. CNSintent can facilitate solving an important biomedical problem: the advanced analysis of copy-number data. The authors are lauded for their excellent Python code and thorough documentation. While the contribution is timely and likely important, there are several areas for improvement.

The manuscript's readability could be better. There are typos, textual errors, and inconsistencies in figure captions, such as incorrect figure references or mismatched values between the text and figures. The "Consistent Segmentation" section is difficult to follow. It is unclear whether this step involves merging pre-existing breakpoints in the data to produce new, longer segments or if larger segments, such as whole chromosomes, are split into smaller, constant-sized segments. The writing suggests that segments are first merged and then split; however, later in the manuscript, they appear to be used separately. In our testing, combining these approaches did not yield

meaningful results. Since consistent segmentation is the method's most critical step, we strongly suggest clarifying this section.

Response: We apologise if the workflow was not fully clear. Both merging and splitting are optional. While technically doing both consecutively is possible, i.e. it is possible to first merge the breakpoints and then subdivide newly created regions, this is not very common in practice and we did not use both steps at the same time. We have updated the first paragraph as follows:

“Segmentation consists of the following 4 steps: (i) define regions of interest (e.g. whole chromosomes, coding genes, etc.), (ii) remove exclusion regions (e.g. telomeric or centromeric regions), (iii) share existing breakpoints between samples and merge them based on a distance threshold, and/or (iv) subdivide the segments into fixed-width bins. Each of the four steps is optional.”

The Results section now describes the segmentations as follows:

“We next set out to explore the effects of different segmentation strategies on the cancer classification task (see Methods). We processed the data using the following segmentation strategies: (i) fixed-size segments of 20 Mb, 10Mb, 5 Mb, 3 Mb, 2 Mb, 1 Mb, 500 Kb, 250 Kb, 100 Kb size; (ii) Whole chromosomes, chromosome arms; (iii) Gene-level CN values based on the ENSEMBL and COSMIC gene sets; and (iv) breakpoint merging using distance thresholds of 1 Mb, 500 Kb, and 250 Kb. The segment sizes roughly cover the ranges used by other authors for feature discovery (Drews et al. 2022).”

We hope that improves the clarity of the process.

The manuscript is unbalanced in its content, with excessive focus on the tool's application and the discoveries derived from it, rather than on the tool itself. This reduces the clarity of the key message. We recommend compressing the application section (deep learning in cancer classification) while expanding the tool description with additional explanations.

Response: We thank the Reviewer for this comment and while we would have loved to reduce the application part of the manuscript in favor of a more detailed explanation of the tool itself, both Reviewer 1 and Reviewer 2 asked for additional data analyses, so we had to find a compromise. In line with your request and also Reviewer 1, we have removed the Integrated Gradients Method altogether and replaced it with a more compact subsection of statistical analysis (section title: Identifying commonly altered regions and outliers) instead. We have also removed not strictly necessary parts including the UMAP analysis, and added more general examples of the application of CNSistent instead. We hope that this improves upon the clarity of the main message of the paper. We have also overall streamlined the application section and edited it for brevity, to further improve on this point.

It is also unclear what type of data the authors are using in the cancer classification section. To improve clarity, this information should be explicitly included in the methods section, detailing the sequencing strategy and copy-number tools used for each cohort.

Response: We have added the following to the data availability:

“For TCGA the ASCAT team called the CNs by ASCATv3 from WGS, the TRACERx team used ASCATv2 from WES and PCAWG consortium published CNs obtained as a consensus of 5 different callers (PCAWG Consortium 2020) from WGS.”

The methods section would benefit from a more detailed explanation of the CNSintent steps. Both Figure 1 and the text leave some parts unclear, particularly in the "Consistent Segmentation" section. Additionally, methods such as random forest and UMAP are only briefly mentioned in a supplementary figure rather than being described in the methods section. Moving these descriptions to the methods section would improve clarity.

Response: We have now clarified the Methods section and updated Figure 1 as

Powered by Editorial Manager® and ProduXion Manager® from Aries Systems Corporation

described in the first comment. We have removed the UMAP analysis as we felt it does not provide enough interesting additional information and it is not part of the CNSistent package, in line with your comment #1. Since the Random Forest method is widely used in the field and not specific to the task we consider here, we decided in the interest of space to not provide a technical description of the method. We detail information about which version of the corresponding Python libraries was used in which part of the analysis in the Methods section:

“The splitting is done using the StratifiedGroupKFold object from scikit-learn v1.4.1, which was also used for ENet and RF classifiers.
For ENet we used the SGDClassifier with log loss and the elasticnet penalty. For RF we used RandomForestClassifier with default parameters.”

Figures are generally clear, but improving color differentiation would be beneficial. For example, in Figure 1, the dark red and dark orange shades are too similar, making them difficult to distinguish. A more optimized color scheme with slightly lighter tones (i.e., increased luminance) would enhance readability.

Response: We agree with the point and we have changed the colors accordingly, as shown below. The dark red and dark orange are now “tab:red” and “tab:purple” respectively. We have also switched all plots to a standard unified palette with more discernible colors. We hope that this improves upon the readability and accessibility of the Figures.

The introduction promotes copy-number signatures; however, these signatures rely on segment lengths and unique breakpoints, which vary between samples. Since this method enforces consistent segmentation and breakpoints across all samples, its applicability to copy-number signatures is unclear. This should be discussed in the Discussion section or removed from the introduction.

We agree with the reviewer that our original intention to promote the use of CN signatures has not been reflected in further sections. CNSistent can generally produce features used for detection of signatures, e.g. breakpoint counts or step sizes, however we have in the end did not develop this to a full extent. Consequently, we have removed mentions of CN signatures from the manuscript altogether.

Out of curiosity: Is it possible to prioritize one type of segmentation over another? For instance, if both WGS and WES data are available, can CNSintent be configured to prioritize WGS calls? Similarly, some tools provide highly precise breakpoint calls that are valuable for detecting fusion genes or rearrangements. In such cases, it would be useful to prioritize these calls and harmonize results from other tools accordingly.

We thank the Reviewer for this suggestion, which is a really interesting one. Unfortunately, currently CNSistent does not support such a weighting of breakpoints. When discussing a potential implementation we realised that there are quite some technical details to be decided upon that would depend from use case to use case and that this is actually a somewhat larger feature that we would like to postpone for the next version of the package.

Terminology Clarifications:

Blacklist, blacklisted regions, gap regions, mask: These terms should be used consistently, particularly since blacklists can be applied at different processing stages. Notably, PCAWG blacklists samples, not regions.

Response: Thank you for the suggestions. To make the text consistent we have used these three exclusive terms:
Gaps refers to the gap regions table as defined by UCSC genome browser. We retained this term without changes.
Region exclusion refers to the process of removing specific regions from downstream analysis. Now defined in methods “Optionally, exclusion regions can be provided to the pipeline to remove locations in the genome where we expect lower quality of information.” All occurrences of the term blacklisting in this context have been removed.

Powered by Editorial Manager® and ProduXion Manager® from Aries Systems Corporation

	Blacklisting refers to removal for samples from the PCAWG dataset which were not marked as "whitelisted". We retained this term without changes.<br><br>Segmentation: The term is commonly used in CNV analysis to refer to inferring continuous genomic segments from raw read counts or probe intensities. Here, it has a slightly different meaning—computing consistent breakpoints across all samples—so a more explicit definition would be helpful.<br><br>Response: Thank you for the comment, we decided to declare the meaning explicitly in the Methods section to avoid any confusion:<br><br>"Note that we use the term segmentation to refer to a consistent segmentation between samples, i.e. a set of positions inside each chromosome that split the chromosome into segments."<br><br>Breakpoint merging/clustering: If these terms are synonymous, choosing one would improve readability.<br><br>Response: Thank you for pointing the issue out. To avoid any further confusion, we have completely removed the term clustering, and now use "merging" throughout the manuscript..<br><br>Coverage: Since "coverage" often refers to sequencing depth, a critical quality metric in DNA sequencing, it might be clearer to use "copy-number coverage" or a similar term. For example, the sentence "Next, samples with low coverage were removed using the…" could be ambiguous if read without context.<br><br>Response: We agree with the possibility of confusion. To prevent it, we have everywhere replaced the term "coverage" with a form of the suggested: "CN-coverage".<br><br>At the end of the subsection "Explainability and the Effect of SOX2 Gene," the phrase "which exhibits significant local amplification in LUSC" should be revised to "which exhibits significant focal amplification in LUSC." The correct terminology is "focal" rather than "local," as established in Beroukhim et al. (2010).<br><br>Response: Thank you for the correction. Due to the changes to this section, the original sentence has been removed altogether.
**Additional Information:**	
**Question**	**Response**
Are you submitting this manuscript to a special series or article collection?	No
**Experimental design and statistics**<br><br>Full details of the experimental design and statistical methods used should be given in the Methods section, as detailed in our Minimum Standards Reporting Checklist. Information essential to interpreting the data presented should be made available in the figure legends.<br><br>Have you included all the information requested in your manuscript?	Yes
**Resources**	Yes

Powered by Editorial Manager® and ProduXion Manager® from Aries Systems Corporation

A description of all resources used, including antibodies, cell lines, animals and software tools, with enough information to allow them to be uniquely identified, should be included in the Methods section. Authors are strongly encouraged to cite Research Resource Identifiers (RRIDs) for antibodies, model organisms and tools, where possible.<br><br>Have you included the information requested as detailed in our Minimum Standards Reporting Checklist?	
**Availability of data and materials**<br><br>All datasets and code on which the conclusions of the paper rely must be either included in your submission or deposited in publicly available repositories (where available and ethically appropriate), referencing such data using a unique identifier in the references and in the "Availability of Data and Materials" section of your manuscript.<br><br>Have you have met the above requirement as detailed in our Minimum Standards Reporting Checklist?	Yes
GigaScience has policies and guidelines in place for the use of generative AI-writing tools such as ChatGPT. If you have used such writing tools to assist with writing the manuscript this must be declared and cited in the text. Authors should not list AI-writing tools and other AI-assisted technologies as an author or co-author and should acknowledge that they are fully responsible for text generated or refined by AI-writing tools.<p><br><br>A summary of use (particularly in the introduction or among methods) needs to be included at the end of the paper, and the outputs should also be included as a supplementary file hosted in GigaDB or other open repositories. Please <a href=https://academic.oup.com/gigascienc	No

e/pages/editorial_policies_and_reporting_ standards target="_new" > read our guidelines for more information. <p><br><br>By submitting to GigaScience, you are aware of the journal's AI-writing tools policy, and if you have declared use of such tools below, you have acknowledged this where appropriate in your manuscript and have made a summary of use and outputs available. <p><br><b>AI-assisted writing tools have been used in the preparation of this manuscript?	

Powered by Editorial Manager® and ProduXion Manager® from Aries Systems Corporation

Manuscript

Click here to access/download;Manuscript;Manuscript CNSistent.pdf

CNSistent integration and feature extraction from somatic copy number profiles

Adam Streck[1,3], Roland F. Schwarz[1,2,3]

1) Institute for Computational Cancer Biology (ICCB), Center for Integrated Oncology (CIO), Cancer Research Center Cologne Essen (CCCE), Faculty of Medicine and University Hospital Cologne, University of Cologne, Germany

2) Berlin Institute for the Foundations of Learning and Data, Berlin, Germany

3) Berlin Institute for Medical Systems Biology, Max Delbrück Center for Molecular Medicine in the Helmholtz Association, Berlin, Germany

Contact: roland.schwarz@iccb-cologne.org

Abstract

The vast majority of cancers exhibit Somatic Copy Number Alterations (SCNAs)—gains and losses of variable regions of DNA. SCNAs play a key role in cancer adaptation through modulation of gene expression, deletion of tumour suppressor genes or amplification of oncogenes. Systematic analysis of SCNAs is now a routine task in both the clinic and research, and can help identify novel cancer genes, improve our understanding of cancer gene regulation and enable us to accurately reconstruct cancer phylogenies. To conduct such analyses however SCNA profiles have to be integrated between samples, patients, and cohorts, an often non-trivial task, for which dedicated toolkits are lacking.

To fill this gap, we developed CNSistent, a Python package for imputation, filtering, consistent segmentation, feature extraction, and visualization of cancer copy number profiles from heterogeneous datasets. We demonstrate the utility of CNSistent by applying it to the publicly available TCGA, PCAWG, and TRACERx cohorts. We compare the effect of sample preprocessing and of different segmentation and aggregation strategies on cancer type and subtype classification tasks using various classification models. We also evaluate how well a classifier trained on one cohort generalizes to another. Lastly, we introduce two segment-based peak and outlier scores to investigate relationships between segments, between samples, and between cancer types. Using these scores, we investigate non-small cell lung cancer samples, highlighting that SOX2 amplification is the dominant copy number alteration in lung squamous cell carcinoma and the main distinction to lung adenocarcinoma.

Keywords

cancer, data processing, SCNA, deep learning, cancer classification

Introduction

Somatic copy number alterations (SCNAs)—gains and losses of long regions of DNA—are found across almost all cancer types and are one of the key defining features separating cancer cells from normal cells[1]. It has been demonstrated that quantifying SCNAs has predictive value in the clinic for both progression free and overall survival [2,3] and that they

can serve as sensitive biomarkers for cancer classification and subtyping[4]. We and others have shown that many cancers demonstrate ongoing chromosomal instability (CIN) and continuously accumulate SCNAs throughout their evolution[5], and that SCNAs are excellent markers for inferring cancer evolution[6,7]. Recently, copy number signatures have linked SCNAs to their underlying molecular mechanisms, further strengthening their prognostic value[8,9].

SCNA profiles are commonly derived from a variety of experimental techniques, including SNP arrays, whole-exome and whole-genome sequencing[10], and recently also increasingly from single-cell sequencing[11]. One major advantage of SCNAs over other genomic data types including somatic single nucleotide variants (SNVs) is ease of handling. Due to their aggregate nature, SCNA profiles of individual patients can be published without concern for privacy and the resulting access restrictions, leading to a growing set of publicly available and easily accessible samples from large cohorts such as TCGA, ICGC[12], and the TRACERx[13] lung and renal cancer cohorts.

Unfortunately, copy number profiles, typically defined as lists of segments with given start and end positions and copy number states, are not directly comparable across samples, patients or cohorts. For example, for phylogenetic reconstructions within a patient, profiles have to undergo minimum consistent segmentation where breakpoints are shared between samples to enable evolutionary comparisons [6,7]. For machine learning classifiers, profiles are often aggregated in fixed-width bins or on the gene level. Additionally, different experimental techniques and different copy number calling algorithms can lead to specific biases, missing data, and varying resolutions, further complicating the matter.

To foster reproducible research and avoid reimplementation of common tasks, a tool that enables integration and joint segmentation and thereby caters to the specific demands of copy number profiles would be desirable. To our knowledge, the only available tool that does not require access to the raw sequencing data is the web-based application CNApp[14], which due to its web-based nature is not easily integratable into data science workflows and was not available at its hosted site at the time of this writing.

To fill this gap, we here present CNSistent, a Python package for preprocessing, consistent segmentation, integration, statistical analysis and visualization of SCNA profiles coming from heterogeneous data sources. We demonstrate the utility of CNSistent by integrating available copy number profiles from the TCGA, PCAWG and TRACERx cohorts. We evaluate various segmentation strategies, comparing the performance of deep learning-based multiclass cancer classification tasks and on the classification of non-small cell lung carcinomas (NSCLC) and demonstrate the use of CNSistent for enabling phylogenetic inference from copy number profiles using the minimum consistent segmentation algorithm.

Methods

CNSistent processes SCNA profiles using a multi-step approach. Input data takes the form of copy number segment tables with either allele-specific or total copy numbers (Fig. 1A). The processing is identical for both allele-specific and total copy numbers, however some of the statistics are limited in the case of copy numbers, as detailed below. Optionally,

exclusion regions can be provided to the pipeline to remove locations in the genome where we expect lower quality of information. CNSistent first calculates the proportions of missing genome, which we here refer to as *CN-coverage*, and then utilises imputation strategies to fill in missing data (Fig. 1B). CNSistent then calculates information about breakpoints in each sample. Using the imputed data here has the advantage that spurious breaks are not created between non-consecutive regions purely by missing data. Once the data are imputed we remove the exclusion regions and calculate statistics relating to aberrant copy number values. In the final step, CNSistent offers various strategies for creating a consistent segmentation across samples (Fig. 1D), which are subsequently aggregated to create a final set of complete SCNA profiles with shared segment boundaries for all samples. The pipeline is fully modular and the steps can be skipped or executed in different order. Note that we use the term *segmentation* to refer to a consistent segmentation between samples, i.e. a set of positions inside each chromosome that split the chromosome into segments.

For its calculations, CNSistent can work with any reference genome; hg19 and hg38 reference assemblies are provided as a default. If the sex of the donors is not provided, CNSistent will determine the sex for each sample based on the presence of the Y chromosome.

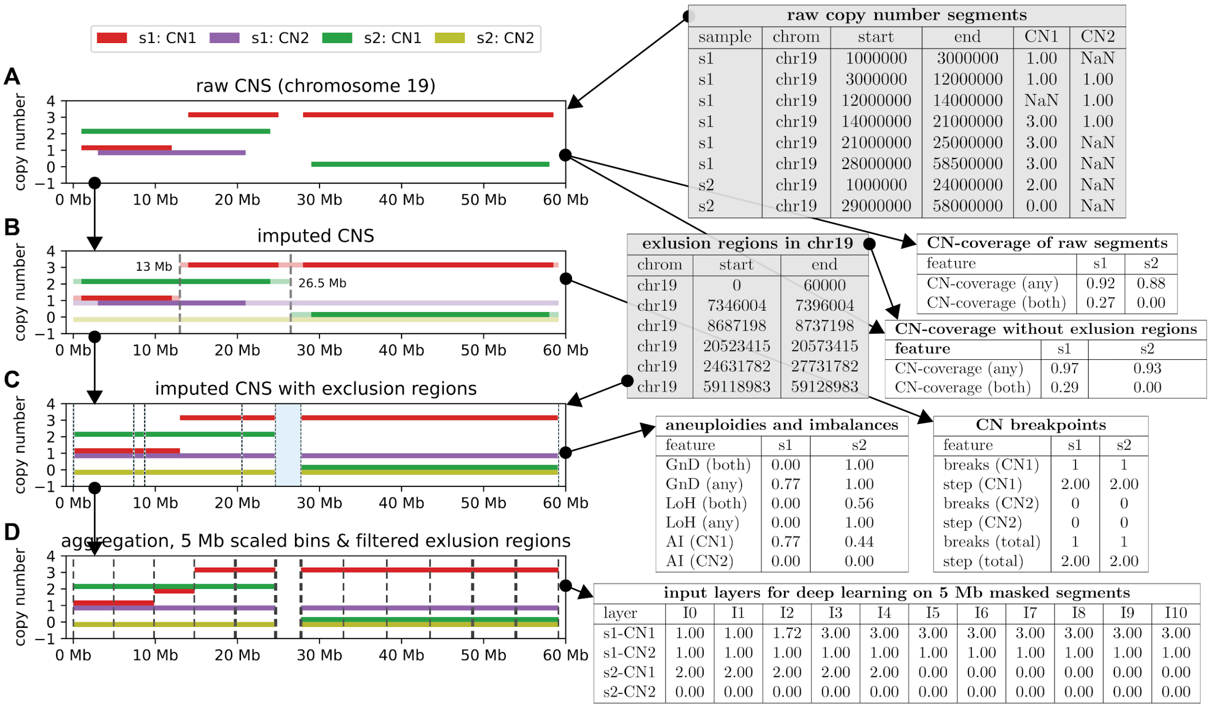

Figure 1: Illustrative example of processing of two SCNA profiles (s1, s2) for two alleles (CN1, CN2), on human chromosome 19. ***A)*** *The input data (gray tables) consist of non-contiguous major and minor copy number segments for each sample. From this the proportion of the genome that is missing is calculated for each sample. For comparison, the CN-coverage is calculated both with and without considering the gap regions. Note that as there are no minor CNs for s2, the homozygous CN-coverage is 0.* ***B)*** *During imputation two new breakpoints are introduced at 13 Mb and 26.5 Mb, while the breakpoints on the boundaries of missing segments are no longer present. From the imputed data CNSistent calculates the CN breakpoint-related statistical features.* ***C)*** *Ploidy and allelic imbalance-related statistical features that are derived from the imputed data and removal of the gap regions.* ***D)*** *Small regions are not used in region exclusion, retaining only the gap between 20 and 30 Mb, which splits the chromosome into two arms, which are then further split into ~5 Mb bins. The same-size strategy is used, meaning that the bins in the left segment are slightly smaller (4.9 Mb), while the ones on the right are slightly bigger (5.27 Mb). Each profile is then converted into a vector of CN values for downstream analysis. Note that as there was a breakpoint at 13 Mb, the resulting value is a weighted mean of the previous values, i.e. 1.72.*

Imputation of missing values

SCNA profiles from different cohorts often vary in the extent to which they span the genome. This can be due to a variety of reasons including different underlying technologies (WES vs WGS sequencing), different segmentation strategies, or different exclusion of regions surrounding the centromeres and telomeres. To retain as much information as possible, CNSistent offers an imputation step capable of filling the gaps in SCNA profiles using an *extension* method (Fig. 1C).

The *extension* imputation method executes the following five steps: (i) Segments are pruned such that they are fully contained within the coordinates and named chromosomes of the reference genome. (ii) CNSistent extends the first and last segment of each chromosome to the chromosome boundaries. (iii) Each gap between two segments is split into two halves (rounded down), and each half is then assigned the CN of its neighboring segment. (iv) If any chromosomes are fully missing from the sample, they are set to 0. (v) The neighboring segments that have the same CN are merged.

Alternatively, two additional imputation options are available: *diploid* and *null*. The diploid method changes the steps (ii-iv) in such a way that all newly created segments are set to diploid, e.g. if a sample is male and major/minor CN columns are used, CNSistent will create a segment on the whole chromosome Y with major and minor CN of 1 and 0 respectively. The null option will analogously fill all the newly created segments with 0.

Feature extraction

CNSistent can calculate a set of statistical features. As CNSistent is sex chromosome aware, the length of the linear genome depends on the sex of the sample. Each feature is therefore calculated three times: for autosomes, for sex chromosomes, and for the whole genome:

CN-coverage: Calculates the proportion of the whole genome where any CN value is assigned (as opposed to missing values). In case of allele-specific CNs, both mono-allelic CN-coverage (either allele has a CN value assigned) and bi-allelic CN-coverage (both alleles must have a CN value assigned) are calculated.

Genome not Diploid (GnD): Defines the proportion of the genome where an allele does not have the CN a diploid cell of the same sex would have. In case of having only total CN this is a lower bound approximation.

Loss of Heterozygosity (LoH): Calculates the proportion of segments with CN=0 on either allele (hemizygous) or on both alleles (nullizygous). The segment is only considered LoH if and only if its CN value is 0 and its normal value is not zero (e.g. chromosome Y for female samples).

Allelic imbalance (AI): The proportion where one allele has a strictly higher CN than the other.

Breakpoints: The number of breakpoints per chromosome for each allele. If two column format is used, a total number of breakpoints is also calculated to account for cases where

both alleles have a breakpoint in the same location (meaning that the total number of breakpoints is less than the sum of the alleles).

Breakpoint Step: The mean difference between the CNs of consecutive segments. Note that it is preferable to impute the segments first to avoid inducing spurious gaps.

Consistent segmentation

One major goal of CNSistent is to obtain segments that are consistent between sample sets and from which then features can be derived in a unified manner. This requires the same set of breakpoints to be present in every sample. Segmentation consists of the following 4 steps: (i) define regions of interest (e.g. whole chromosomes, coding genes, etc.), (ii) remove exclusion regions (e.g. telomeric or centromeric regions), (iii) share existing breakpoints between samples and merge them based on a distance threshold, and/or (iv) subdivide the segments into fixed-width bins. Each of the four steps is optional.

The segments for step (i) can be provided as a BED file, or one of five predefined options can be used: whole chromosomes (default option), chromosome arms, cytobands, COSMIC consensus cancer gene set[15], or the Ensembl coding genes set[16]. From these segments, exclusion regions can be optionally removed (Fig. 1D). As a default option, the regions of low mappability as defined by the UCSC[17] genome browser are provided. During the exclusion process, if the regions are small or close to each other, fragmentation can occur. This can be avoided by segment filtering—the user specifies a filter of size f, where any exclusion region smaller than f is removed, and likewise if after the exclusion regions are removed from segmentation, any newly created segments smaller than f are also removed.

The breakpoints are then merged using a greedy algorithm on a predefined region (usually a whole chromosome). Starting from the leftmost breakpoint, all breakpoints within the merge distance m are accumulated and a new breakpoint is created at their average. This is then repeated from the leftmost not yet merged breakpoint, until the end of the region is reached. A detailed example is shown in Supp. Fig. 1.

Lastly, the resulting segments can be subdivided into smaller bins based on user defined split size s (Fig. 1E). Three subdivision strategies are provided: (a) From the start of the segment, breakpoints are inserted every s bases. Here, the last bin is likely to be of a different size. If it is smaller than $s/2$, it is merged with the previous segment. (b) Is similar to (a) where instead of creating the padding only at the end, the padding is split in half and added to both ends. Likewise, if the first and last bins are smaller than $s/2$, they are merged with their neighboring segments. (c) The bins are scaled so that they are all the same length, slightly different from s. Consider a segment that has c bins, including the padding–If the padding is smaller or equal to $s/2$**,** split the segment into $c - 1$ equally sized bins, otherwise into c bins.

Aggregation of copy numbers

After joint segmentation, the copy numbers from the original segments are aggregated to create CNs for the new segments. First the old segments are split at the breakpoints given by the new segmentation. Second, the resulting refined segments are aggregated between the breakpoints given by the segmentation, using one of four possible aggregation

strategies: The *Min* and *Max* strategies will assign the minimum or maximum CN to the whole segment–the *Min* strategy is particularly relevant when considering genes, since incomplete segments are unlikely to yield functional gene copies. The *Mean* strategy will take a mean of CNs across bins weighted by their lengths, preserving the overall CN per sample. Lastly, merging can be skipped altogether, which can be used if we want to select only a subsection of each profile, e.g. only q-arms.

Sample filtering

The features obtained in the feature extraction step can be used to filter undesirable samples. For base quality metrics, like CN-coverage, a simple z-score outlier detection method is provided, meaning that for a feature f over a set of samples S, $z = \frac{f(S) - \mu(f(S))}{\sigma(f(S))}$ is calculated and samples greater than 3 standard deviations from the mean ($|z| \geq 3$) are removed. The value 3 is a typical threshold for the method, but it can be adjusted by the user.

In certain cases, a qualitative separation of data is preferable, e.g. to remove samples with negligible SCNA activity. CNSistent offers an automated solution for finding such thresholds using a knee-detection algorithm. A knee-point is a point of the plot where the maximum angle between the line to the first point and the last point of the plot. To find the knee-points for a feature f in a set of samples S, a tuple of monotonically increasing feature values $T = (min(f(S)), ..., max(f(S)), \forall i \in 1, |T| - 1: t_i \leq t_{i+1}$ and a cumulative distribution of values smaller than each threshold $Y = (|f(S) \leq t|)_{\{t \in T\}}$ is created. Second, T is normalized such that $\forall t \in T$: $t' = \frac{t - t_1}{t_n - t_1}$, and analogously for Y'. The knee-point is then the $t_i, 1 \leq i \leq n$ with the maximum angle between the vector from origin to the normalized threshold, (t_i', y_i'), and the vector from the threshold to the endpoint, $(1 - t_i', 1 - y_i')$. If the angle is negative (clockwise rotation), we call it a *knee*, otherwise (counter-clockwise rotation), we call it an *elbow*. A visualization of the method is provided in Supp. Fig. 2.

Outlier detection

CNSistent can detect outlier samples based on the normalized Manhattan Distance (NMD) between pairs of samples. To calculate NMD we normalize each sample by dividing the value of each bin by its sum. This normalization allows us to ignore the effects of whole genome doubling, since the normalized values are the same before and after WGD. Formally, having two aggregated samples $S = (s_1, \cdots, s_n)$, $R = (r_1, \cdots, r_n)$, the $NMD(S, R) = \sum_{j=1}^{n} |\frac{s_i}{\Sigma(S)} - \frac{r_i}{\Sigma(R)}|$. To compare a sample S to a cluster of samples $C = (S_1, \cdots, S_m)$, we calculate the outlier score $OS(S, C) = \frac{\sum_{j \in 1}^{m} NMD(S_i, S_j)}{|C|}$. To compare between two cancer types $C1, C2$ and a sample $S \in C1$ we extend the outlier score as $OS(S, C1, C2) = OS(S, C2) - OS(S, C1)$.

Peak detection

To find regions of interest in the samples, CNSistent provides the peak score (PS), which shows how much each bin differs from its neighbours. Have an aggregated sample $S = (s_1, \cdots, s_n)$ we set the boundary values $s_0 = s_1$, $s_{n+1} = s_n$ and calculate $\forall i \in (1, \cdots, n)$: $PS(S, i) = (s_i - s_{i-1}) - (s_{i+1} - s_i)$. This score will be positive for segments higher than their neighbours, negative for those lower and close to zero for segments with monotonous behaviour. We therefore use the PS to detect the highest and lowest values, which show the locations of most abrupt change in CN accumulation. Note that for meaningful calculation this requires that the segments are connected to each other and about the same size.

Identifying discriminatory features

To identify features that most differ between groups of samples, we use the Mann-Whitney U-Test using the `mannwhitneyu` function in scipy v1.15.0. All p-values are corrected for multiple tests using the `multipletests` function with Benjamin-Hochberg correction in statsmodels v0.14.0.

Machine learning

To evaluate how different filtering and segmentation strategies affect the downstream analysis, we used two cancer type classification tasks: classifying between 6 types with the most samples, as introduced in *Attique et al.*[18] and NSCLC classification, as introduced in *Qiu et al.*[19]. In this task, each binned sample as illustrated in Fig. 1 represents one feature vector. The output probability that a sample belongs to each cancer class under consideration. We then compare 4 different classification methods: Random Forest (RF), Elastic Net (ENet), Deep Neural Network (DNN), and Convolutional Neural Network (CNN).

For each of these models we apply 5-fold cross validation, i.e. we split each dataset into 5 groups and always withhold one while training on the other 4. The validation accuracy for each model is then the mean of the test scores of the 5 different splits[20].

As the number of patients per cancer type varies, the classes are imbalanced. To avoid a possible bias due to an overrepresentation of one class, a stratified split is used, meaning that the ratio of the individual cancer classes is preserved across the 5 subsets. Additionally, some samples are obtained through multi-region sampling. While the samples from different regions show different profiles, there is a risk of being able to guess the class based on the similarity to the original profile. This is prevented by sample grouping where each group (in this case patient) can only be part of one subset. The splitting is done using the `StratifiedGroupKFold` object from scikit-learn v1.4.1, which was also used for ENet and RF classifiers.

For ENet we used the `SGDClassifier` with log loss and the `elasticnet` penalty. For RF we used `RandomForestClassifier` with default parameters. For deep learning we used CNN, and DNN3 neural network architectures as described in Attique et al[18], as well as our own extended CNN, which we called CNN+. In summary, the CNN uses two convolutional layers (kernel=5) and RelU activation, followed by maxpool, batch

normalization and dropout after each, followed by a flattening and the output layer with softmax. The DNN3 uses 3 hidden layers with sizes 600, 300, and 150, with batch normalization, dropout and RelU, except for the output layer which uses softmax. The following was not declared in the manuscript and therefore has been set to default PyTorch values: maxpool kernel size of 2, dropout probability of 0.5. Our CNN+ model builds on the CNN, however an additional fully connected layer is added after the flattening layer, with size half in between the flattening and output layer. The CNN+ also uses two separate input channels, one for each allele. The full architecture of CNN+ is given in Supp. Fig. 3.

Optimization was done using the PyTorch library v2.2.1[21], accelerated using CUDA v12.1. Optimization was conducted using the Adam optimizer with a learning rate of 0.001, weight decay of 0.01 and batch size of 64. The error is evaluated using cross-entropy loss. The training was limited to 1000 epochs. The training process was accelerated by an early stopping strategy where the minimum loss is recorded and if past 10 epochs lead to a training loss higher than the existing global minimum, the training stops. To accommodate for the two alleles, we have concatenated the major and the minor CNs into a single vector.

Data and code availability

CNSistent package, source data, and plotting notebooks can be downloaded at: https://bitbucket.org/schwarzlab/cnsistent and available on PyPI: https://pypi.org/project/CNSistent/. CNSistent is registered on bio.tools with ID: `cnsistent` and at SciCrunc with RRID: **SCR_027025**. The preprocessed input data are available at https://zenodo.org/records/14677713[22]. The data produced by CNSistent are available at https://zenodo.org/records/14547456[23]. The deep learning code and results are available at https://zenodo.org/records/14546762[24]. The deep learning model information is stored in the DOME registry: https://registry.dome-ml.org/review/59bcqzam2c [25].

The data have been obtained from the following sources, accessed in December 2023: PCAWG data obtained from: https://dcc.icgc.org/releases/PCAWG/consensus_cnv[12]; The results published here are in part based upon data generated by the TCGA Research Network: https://www.cancer.gov/tcga. TCGA data obtained from ASCATv3 at: https://github.com/VanLoo-lab/ascat[26]. TRACERx data obtained from: https://zenodo.org/records/7649257[13]. COSMIC cancer set obtained from: https://cancer.sanger.ac.uk/census[27]. Human genome gene set obtained using PyENSEMBL (2023)[16]. Cytoband and Gap data obtained from the UCSC Genome Browser: https://genome.ucsc.edu[28]. For TCGA the ASCAT team called the CNs by ASCATv3 from WGS, the TRACERx team used ASCATv2 from WES and PCAWG consortium published CNs obtained as a consensus of 5 different callers[29] from WGS.

Results

We illustrate the use of CNSistent on a cancer type classification task using 15,072 publicly available SCNA profiles from The Cancer Genome Atlas (TCGA[9], n=10674), the Pan-Cancer Analysis of Whole Genomes (PCAWG[12], n=2778) and the TRACERx cohort of non-small cell lung cancer[13] (n=1620).

Where the TCGA and PCAWG datasets overlap (829 samples), we gave preference to the PCAWG callset. The PCAWG dataset blacklists 195 low-quality samples, which were removed before further processing.The TRACERx dataset consists of two parts, primary tumor samples (n=1428) and primary with metastatic samples (n=694). We used the primary sample set in the 502 samples on which they overlap. This yielded a total set of 14174 SCNA profiles which were subjected to CNSistent for pre-processing and integration. A summary of sample counts is provided in Supp. Table 1.

CNSistent segmentation of 14174 copy number profiles

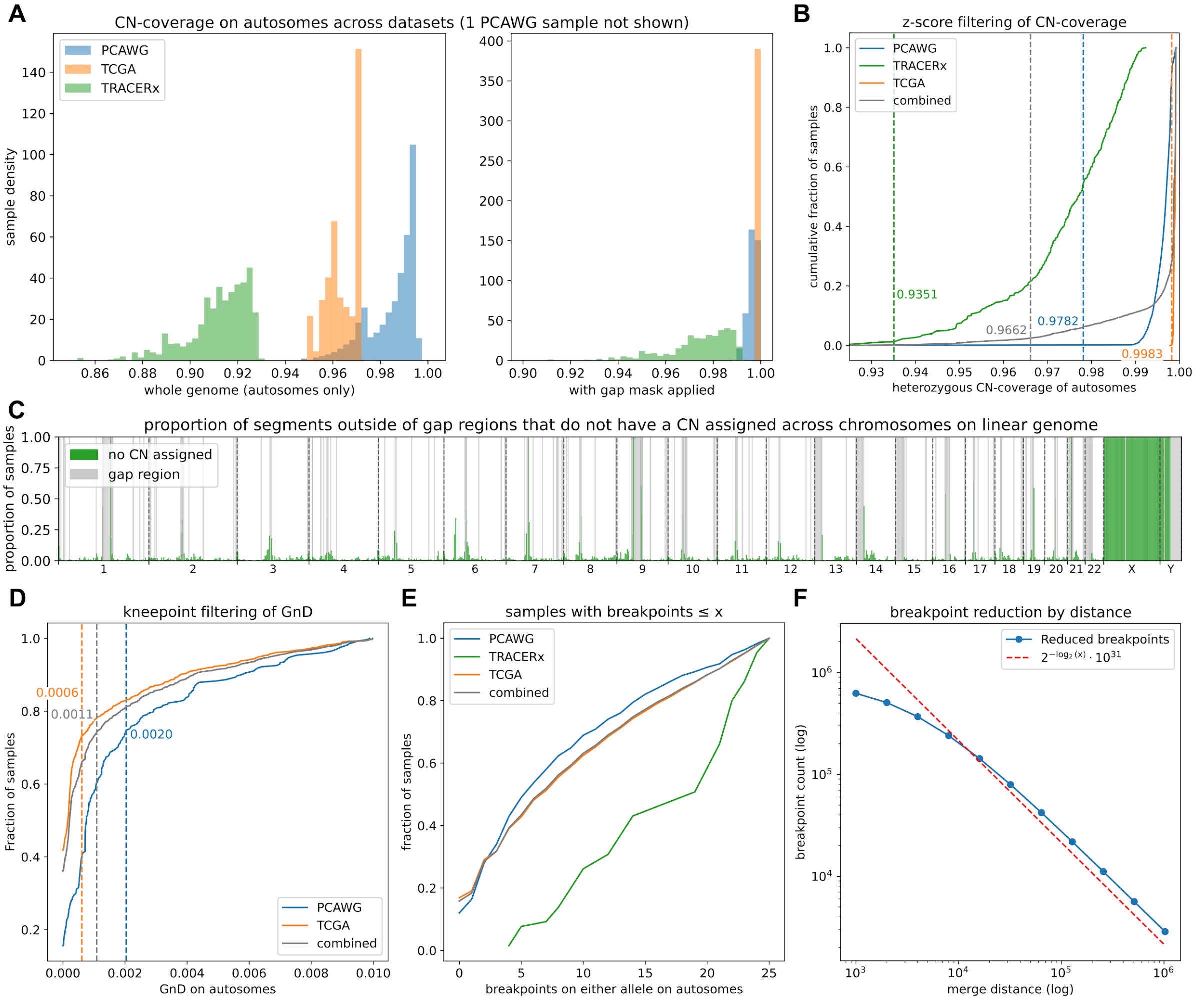

Figure 2: Processing of the PCAWG, TRACERx, and TCGA datasets. ***A)*** *Histograms of heterozygous CN-coverage before and after gap region filtering. Note that the PCAWG and TCGA datasets have almost full CN-coverage after filtering. In contrast, while TRACERx shows a major shift, there are still substantial portions missing.* ***B)*** *Cumulative distribution of samples by heterozygous CN-coverage, with the threshold for filtering given by the z-score. The position of the threshold is much higher for the combined dataset compared to the individual ones.* ***C)*** *Distribution of the missing values in the TRACERx dataset along the linear genome (X and Y*

are not present in the data). Data are mostly missing in regions close to the centromeres and telomeres, in particular for chromosomes 1 and 9. ***D)*** *Cumulative distribution of GnD for a subset of samples below 1%. TRACERx is not shown as none of the samples has hemizygosity below this value. Note the clear slope change around 0.1%, also detected by our kneepoint algorithm.* ***E)*** *Cumulative distribution of breakpoint counts for a subset of samples with less than or equal to 25 breakpoints. The curve is almost linear for all datasets, demonstrating that there's no clear cutoff value in this region.* ***F)*** *The result of breakpoint reduction using 11 log-distributed merge distances between 1 Kb and 1 Mb. Note that the relationship is proportional—doubling the distance leads to halving the number of resulting segments, as shown by the hyperbolic curve.*

We started by imputing any missing data and calculated the sample features (see Supp. Fig. 4 for complete results). Since SCNA profiles for sex chromosomes were not available in the TRACERx cohort, all sex chromosomes were removed from further analysis. Before region exclusion, the SCNA profiles covered on average 98.47%, 96.39%, and 91.18% for PCAWG, TCGA and TRACERx respectively (Fig. 2A). When using the UCSC gap regions for exclusion, the CN-coverage rose to 99.62%, 99.89%, and 97.38%. The gap regions of hg19 on autosomes sum to 19.65 Mb, which is 6.82% of the total genome. For TCGA and PCAWG virtually all the missing segments fell into these gap regions. In TRACERx, there are regions missing also outside these gap regions, however mostly on their boundaries (Fig. 2C). This was likely due to the sequencing method: PCAWG data has been sourced using WGS, whereas TCGA combines multiple data sources.

Next, samples with low CN-coverage were removed using the z-score based outlier detection (Methods). Thresholds were calculated for each of the datasets separately as well as using the combined dataset of all samples (Fig. 2B). For the individual samples there was only a small set of outliers: 3, 16, and 19 for the thresholds of 97.82% for PCAWG, 99.83% for TCGA, and 93.51% for TRACERx respectively. However, when the combined dataset was used, 352 samples were below the detected threshold of 96.62%, stemming from the fact that the CN-coverage distribution of TRACERx significantly differs from the other two. In this case, filtering each set separately leads to significantly lower removal rate. Additionally, one sample in the PCAWG dataset, SP107557, had CN-coverage of only 57.67% and presumably should have been blacklisted in the original dataset.

We also removed samples with few or no copy number alterations. Other authors have used the number of breakpoints[9] as evidence for SCNAs, however we have not observed a clear knee-point in the data (Fig. 2E) and any threshold would therefore be arbitrary. Instead, we used the knee point detection algorithm on the GnD statistic for samples below 1% GnD to determine the following cutoffs (Fig. 2D): 0.06% for TCGA (745 samples removed) and 0.2% for PCAWG (211 samples removed). For TRACERx all samples were retained. The filtering process then leads to the final filtered sample set of 12901 samples (see Supp. Fig. 5 for full sample distribution).

We next evaluated the effects of breakpoint merging. Without any merging, the whole filtered dataset has 826910 unique breakpoints, i.e. one breakpoint per 3.7 Kb on average. We explored different merge distances from 1 Kb to 1 Mb, leading to reductions between 24.39%-99.65% (Fig. 2F), and selected 1 Mb, 500 Kb, and 250 Kb distances, leading to 2797, 5569, and 10797 autosomal segments respectively. To compute all combinations of segmentation strategy and datasets efficiently, we made use of CNSistent's internal parallelization strategy. Runtime decreased in a near-linear fashion with the number of compute cores available (Supp. Fig. 6). All segmentation configurations are listed in Supp. Table 2.

Evaluating segmentation strategies on a cancer classification task

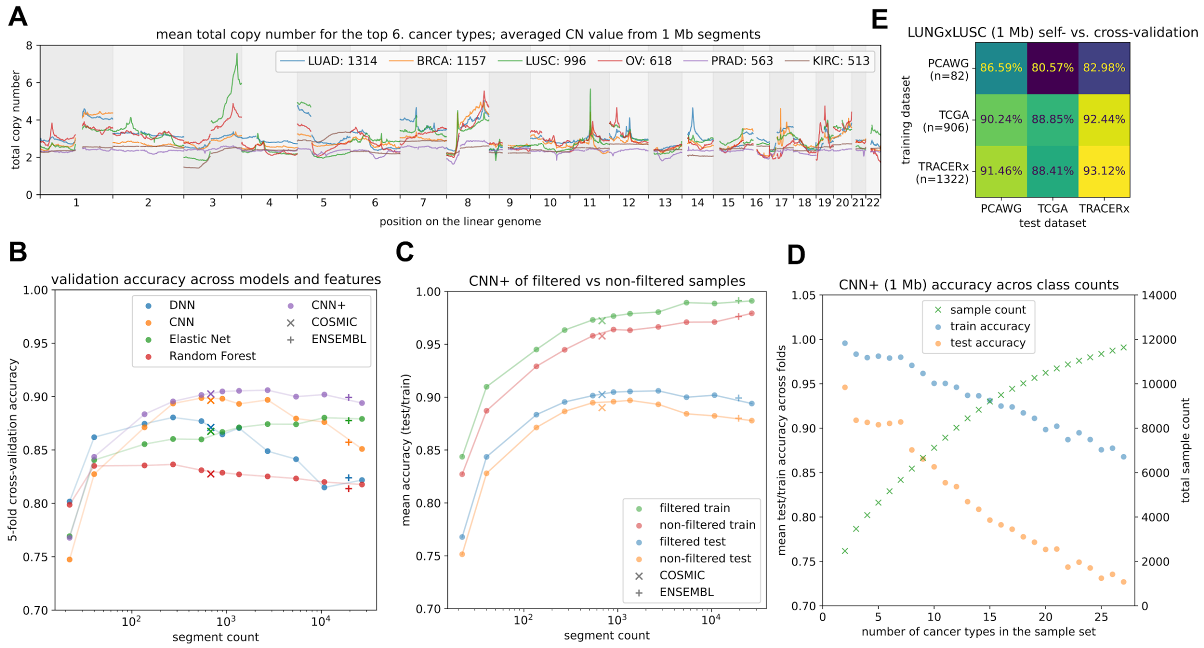

Figure 3: Evaluation of multi-class prediction task. ***A)*** *Averaged SCNA profiles of the 6 cancer classes considered for classification.* ***B)*** *Validation accuracy of the different models tested across increasing granularity of segmentation. DNN and RF quickly reach maximum accuracy and degrade with an increasing number of segments, while the CNN and CNN+ architectures increase until ~1000 segments and ENet even increases monotonously. Results of training on gene-based CNs for each model are displayed using crosses (the number of genes then gives the number of segments).* ***C)*** *Comparison of training on filtered and unfiltered data. We see that both the train and test accuracy improves after filtering. Additionally we see that the training accuracy increases almost monotonously, while the validation degrades after ~1000 segments, likely pointing to overfitting on smaller segments.* ***D)*** *Results of classification across 2-27 classes on 2 Mb segments. We see nearly linear degradation of both training and testing accuracy, however even for 27 classes the accuracy is still over 70%.* ***E)*** *The NSCLC classification task using 2 Mb segments. On the diagonal the models are scored using 5-fold cross-validation on each individual dataset. The remaining values show results of training on one full dataset (row) and validating on another full dataset (column). We can see that in particular training on bigger sets of TCGA and TRACERx yields better results on PCAWG than training on self.*

We next set out to explore the effects of different segmentation strategies on the cancer classification task (see Methods). We processed the data using the following segmentation strategies: (i) fixed-size segments of 20 Mb, 10Mb, 5 Mb, 3 Mb, 2 Mb, 1 Mb, 500 Kb, 250 Kb, 100 Kb size; (ii) Whole chromosomes, chromosome arms; (iii) Gene-level CN values based on the ENSEMBL and COSMIC gene sets; and (iv) breakpoint merging using distance thresholds of 1 Mb, 500 Kb, and 250 Kb. The segment sizes roughly cover the ranges used by other authors for feature discovery[9].

To our knowledge the best result to date on the cancer classification task has been reported on classification of top 6 cancer types in the dataset in Attique *et al.*[18], with up to 92% test accuracy on the best model. Using our combined dataset, the selection of top 6 classes resulted in a set of 5172 samples with the following class labels: lung adenocarcinoma (LUAD, n=1314), breast invasive carcinoma (BRCA, n=1157), lung squamous cell carcinoma (LUSC, n=996), ovarian cancer (OV, n=618), prostate adenocarcinoma (PRAD, n=563), and kidney renal cell carcinoma (KIRC, n=513), with their mean profiles displayed in Fig. 3A.

We compared the DNN3 and CNN architectures of Attique *et al.*, Random Forest, Elastic Net, and our extension of the CNN architecture–CNN+ (Fig. 3B)–across decreasing segment sizes. On whole chromosomes the most performing models reached a validation accuracy of ~80% and considering the arms separately reached almost ~86% on the DNN architecture. Increasing the resolution improves accuracy of the two convolutional models, which peak in the region of around 1000 segments. The best validation (mean of 5 folds) accuracy of 90.60% was achieved with the CNN+ at 1 Mb segments, with full confusion matrix given in Supp. Fig. 7. We used the 1 Mb segments for the subsequent tasks, however sizes from 5 Mb to 250 Kb all had validation accuracy above 90%, and we would therefore consider all of them to be suitable for any further analyses.

The RF and DNN models however peak around 200 segments and increasing the resolution further decreases the validation performance, likely due to overfitting. The only architecture that improved monotonously was ENet, where the penalty regularization seemed to prevent overfitting, however from 20 Mb onwards it always underperforms compared to the CNN+. Comparing the full segmentation with the COSMIC and ENSEMBL gene sets we saw that taking only the CNs for genes performs equivalently to creating a segmentation with a similar number of features. Breakpoint merging performed comparably to bins of the same size, e.g. a 500 Kb merge window showed an accuracy of 90.09%, while 500 Kb segments showed an accuracy of 90.0%. Similarly, considering different aggregation strategies for COSMIC and ENSEMBL has not affected the results significantly: for COSMIC the results were Min: 90.94% Mean: 90.25%, Max: 90.05%. For ENSEMBL: Min: 87.44%, Mean: 89.92%, Max: 89.54%. The best test accuracy (maximum of 5 folds) was 92.42% on 1 Mb segments with the CNN+ model. This is slightly above the best test accuracy of Attique *et al.* (92%). All the deep learning models trained within 100 seconds on a desktop GPU. Full training times are shown in Supp. Fig. 8. Full training and test results are given in the Supp. Table 3.

To evaluate the results of filtering, we have compared the results on filtered (5161 samples) and unfiltered (5257 samples) on the CNN+ model (Fig. 3C). We saw that both training and testing accuracy has been consistently better in the filtered dataset. The average test score improvement was 1.28%. Additionally we were interested in how the CNN+ performs for different numbers of classes. We limited ourselves to classes with at least 100 samples; this yielded 27 classes (Supp. Fig. 5). In Fig. 3D it can be seen that the accuracy is quite high for all the cases and decreases in almost a linear fashion. In the easiest binary classification task we saw 94.6% validation accuracy, while the 27-class task reached 72.69%.

To demonstrate the potential of integration using CNSistent across different datasets we used the NSCLC classification task, training the models on one dataset and validating on another (Fig. 3E). We see that the accuracies of models trained on a different dataset match or sometimes even outperform models trained and validated on the same dataset, with up to 91.46% accuracy for the TRACERx model applied to PCAWG. We also see that compared to self-training, the models trained on bigger sets (TRACERx, TCGA) outperform self-training on the small PCAWG model. Likewise, training on TRACERx slightly outperforms self-training on TCGA. The 5-fold cross-validation accuracy on the combined dataset was 92.73%, considerably improving on the previous result of 84% in *Qui et al.*[19]. When training the models individually, we obtain only 91.21% mean validation accuracy, showing that combining the datasets leads to a (1.52%) improvement.

Identifying commonly altered regions and outliers

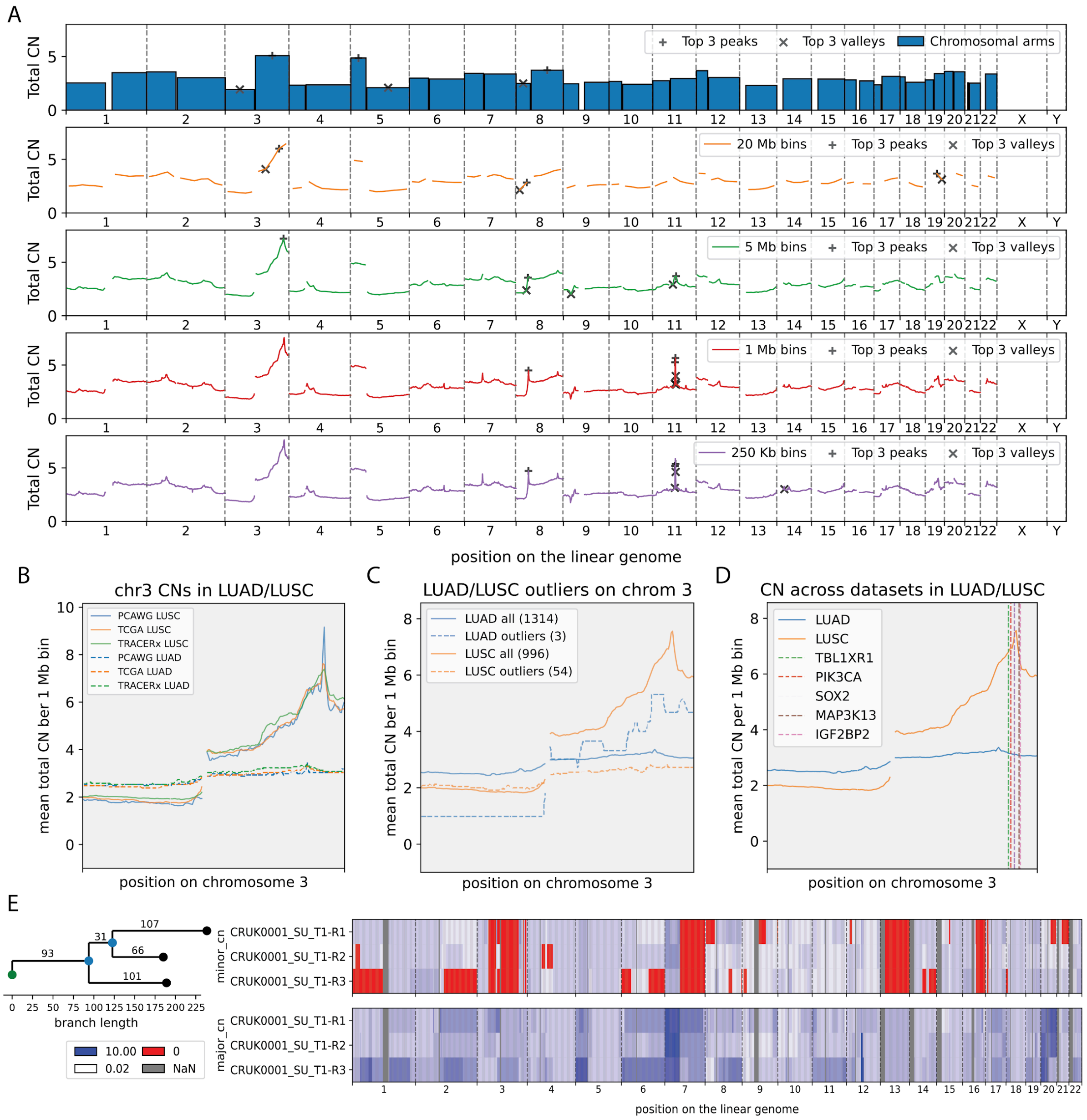

Figure 4: Analyses of LUAD/LUSC CN profiles. Except for the phylogenetic tree, all plots are produced using CNSistent segment plotting functions. ***A)*** *The mean CN profiles of the LUSC samples across different segmentations. For each segmentation, the segments with the 3 highest positive (peaks) and highest negative (valleys) peak scores are shown.* ***B)*** *The 2Mb CN profiles between different cohorts for both LUSC and LUAD, showing strongly correlated patterns of selection between the cohorts of the same cancer type.* ***C)*** *Mean CN profile for LUAD and LUSC with the positions of the top 5 most copy number changed genes.* ***D)*** *Mean CN profiles of both LUAD and LUSC compared to the outlier samples (samples with high NMD its own type and low to the other type).* ***E)*** *Example of CN heatmap for major and minor CN values in three regions of a single tumor together with inferred phylogeny.*

Lastly we demonstrate our segment-derived metrics on the LUNG-LUSC sample set. First, we investigated how the identification of recurrently altered genomic regions is influenced by the segmentation strategies using our simple peak detection algorithm (Methods). We found that the regions with the highest and lowest peak scores differ between segmentation sizes (Fig. 4A), with chr3 being detected mostly in big segments due to a wide slope on the q-arm, while chr8 is being mostly detected in middle sizes due to focal amplification at the end of

the p-arm, and on chr11 there is very narrow peak which becomes most prominent in smaller segmentations.

Next, we investigated all CN profiles for outliers (Methods). The highest NMD between LUAD and LUSC samples is on chromosome 3, where we can see that LUSC has a distinctive, wide peak on chr 3q (Fig. 4B), while LUAD is mostly neutral. This pattern is extremely well correlated across cohorts. We then calculated the outlier score between LUAD and LUSC and used the kneepoint detection to find an outlier threshold (Supp. Fig. 9), finding 3 LUAD samples with LUSC-like pattern (amplification of SOX2) and 54 LUSC samples with neutral LUAD-like pattern (Fig. 4C). We also observed that the majority of these samples (58.75%) came from the TCGA dataset, while the TRACERx dataset had the least outliers (15.79%).

To systematically determine which genes differ significantly in their CNs between LUAD and LUSC, we conducted a Mann-Whitney U-Test with Benjamini-Hochberg correction on the mean CNs of the COSMIC genes. Out of 722 genes, 599 had adjusted p-value below 0.05. The top 5 most copy number altered genes were all on the q-arm of chr3 (Fig. 4D). All these had the adjusted p-value below 10^{-169}, with SOX2[30] being the most significant at $p \approx 10^{-187}$. The SOX2 gene also has the highest mean CN of 7.56.

Lastly, to validate CNSistent segments outside of the tool, we have used the 1 Mb segments for phylogeny reconstruction. For this we have used MEDICC2[6] and applied it to the first patient in the TRACERx dataset with 3 regions. A CONSistent-produced bar plot of the major and minor CNs and a MEDICC phylogenetic tree are shown in Fig. 4E.

Discussion

We have introduced CNSistent, a new Python-based library for processing and exploratory data analysis of SCNA profiles. Using the PCAWG, TCGA, and TRACERx datasets.The main goal of CNSistent is to provide the user with tools for easy data processing so that SCNA profiles can be jointly used for downstream analysis. There are tools available that call CNs from various sequencing data and provide related visualizations in Python, e.g. CNVKit[31] or Segmentum[32], with many more outside Python, with comparison studies done e.g. by Masood et al.[33]. On the analysis side, there are many well known tools for detection of regions of interest, in particular GISTIC[34] and BISCUT[35], which take SCNA profiles and combine them, however, this is done internally by the tool and not accessible or controllable by the user. To the best of our knowledge the only tool for integrative analysis of SCNA profiles is the web-based CNApp[14], which shares some of the functionality with CNSistent, in particular re-segmentation and calculation of profile statistics. However, CNApp is designed for analysis within a web dashboard, while CNSistent serves as a tool for the integration of data before application of downstream tools. We did not fully compare the tooling to CNApp as the hosting was not available at the time of writing.

Using the filtered and combined datasets, we compared several segmentation methods in providing features for a cancer type classification task. We observed that the relationship between segmentation size and model accuracy is highly model dependent: the Random Forest model quickly started to overfit, while the Elastic Net improved near-linearly with the number of segments. In our best performing model, segmentations within the region of 5 Mb

to 500 Kb performed quite equivalently, and also matched the results obtained when classifying based on the hand-picked list of COSMIC[15] cancer genes. Unfortunately, the comparison of our models to the one from *Attique et al*[18] was partially limited by the fact that the authors did neither provide all of the model parameters, nor the model source code, and that the source dataset was no longer available at the time of writing this article. Validation on a hold-out set or cross-validation has not been conducted by the authors, we therefore only performed our comparisons on the test accuracies. All models and the input dataset used in this study are available online (see Data and code availability section). The main purpose of the classification task in this work was to evaluate different segmentation sizes. We assume that better performing models could still be developed by fine-tuning for a particular segmentation or cancer type.

To investigate whether the results are consistent between cohorts, we compared classification between NSCLC cancers in all three datasets and saw that the per-sample accuracy improved by combining these three studies when compared to classification of each of them separately. We saw that models trained on one dataset can be successfully applied to classify another, sometimes even outperforming the source dataset, demonstrating that the classifier generalizes well. We also saw that our model trained on the joint dataset had a better accuracy than the average of the individual models trained on the individual dataset. On the joint dataset our model also considerably outperformed the previous result of *Qiu et al.*[19]. We therefore conclude that it is worthwhile to aim to integrate datasets from heterogeneous sources.

To show the utility of sample integration in analysis, we investigated the NSCLC samples using statistical methods. We identified chromosome 3 as the region of interest, in particular in the context of LUSC, with a wide peak in the location of the SOX2 gene, a well-known actor in LUSC[36]. Using gene-based segments we conducted a statistical test to find the most differently altered genes between LUSC and LUAD, which likewise all additionally we used a normalized Manhattan Distances score to detect outlier samples, with our detected outliers showing the selection pattern of the other cancer, possibly hinting at either mislabelling or co-occurrence of both cancers in the outlier samples[37]. Arguably, these results primarily demonstrate the applicability of our method and warrant further detailed investigations. Future work might also focus on developing methods for within-sample comparison of segments, as well as between-samples and between-types distance calculations.

Acknowledgements

The results published here are in part based upon data generated by the TCGA Research Network: https://www.cancer.gov/tcga.

The authors would like to thank Tom L. Kaufmann, Cody B. Strange, Philipp G. Keyl, and Tom B. K. Watkins, Thomas J. Y. Kono, Laura Godfrey, and Daniel Schütte for their feedback.

Literature

1. Beroukhim, R. *et al.* The landscape of somatic copy-number alteration across human cancers.

Nature **463**, 899–905 (2010).

2. Jamal-Hanjani, M. *et al.* Tracking the Evolution of Non–Small-Cell Lung Cancer. *N. Engl. J. Med.* **376**, 2109–2121 (2017).
3. Turajlic, S. *et al.* Tracking Cancer Evolution Reveals Constrained Routes to Metastases: TRACERx Renal. *Cell* **173**, 581–594.e12 (2018).
4. Pan, X. *et al.* Identification of the copy number variant biomarkers for breast cancer subtypes. *Mol. Genet. Genomics* **294**, 95–110 (2019).
5. Watkins, T. B. K. *et al.* Pervasive chromosomal instability and karyotype order in tumour evolution. *Nature* **587**, 126–132 (2020).
6. Kaufmann, T. L. *et al.* MEDICC2: whole-genome doubling aware copy-number phylogenies for cancer evolution. *Genome Biol.* **23**, 241 (2022).
7. Watkins, T. B. K. *et al.* Refphase: Multi-sample phasing reveals haplotype-specific copy number heterogeneity. *PLoS Comput. Biol.* **19**, e1011379 (2023).
8. Steele, C. D. *et al.* Signatures of copy number alterations in human cancer. *Nature* **606**, 984–991 (2022).
9. Drews, R. M. *et al.* A pan-cancer compendium of chromosomal instability. *Nature* **606**, 976–983 (2022).
10. Gabrielaite, M. *et al.* A Comparison of Tools for Copy-Number Variation Detection in Germline Whole Exome and Whole Genome Sequencing Data. *Cancers* **13**, 6283 (2021).
11. Kuipers, J., Tuncel, M. A., Ferreira, P. F., Jahn, K. & Beerenwinkel, N. Single-cell copy number calling and event history reconstruction. *bioRxiv* (2024) doi:10.1101/2020.04.28.065755.
12. Zhang, J. *et al.* The International Cancer Genome Consortium Data Portal. *Nat. Biotechnol.* **37**, 367–369 (2019).
13. Al Bakir, M. *et al.* The evolution of non-small cell lung cancer metastases in TRACERx. *Nature* **616**, 534–542 (2023).
14. Franch-Expósito, S. *et al.* CNApp, a tool for the quantification of copy number alterations and integrative analysis revealing clinical implications. *Elife* **9**, e50267 (2020).
15. Tate, J. G. *et al.* COSMIC: the Catalogue Of Somatic Mutations In Cancer. *Nucleic Acids Res.* **47**, D941–D947 (2019).
16. Harrison, P. W. *et al.* Ensembl 2024. *Nucleic Acids Res.* **52**, D891–D899 (2024).

17. Perez, G. *et al.* The UCSC Genome Browser database: 2025 update. *Nucleic Acids Res.* (2024) doi:10.1093/nar/gkae974.
18. Attique, H. *et al.* Multiclass Cancer Prediction Based on Copy Number Variation Using Deep Learning. *Comput. Intell. Neurosci.* **2022**, 4742986 (2022).
19. Qiu, Z.-W., Bi, J.-H., Gazdar, A. F. & Song, K. Genome-wide copy number variation pattern analysis and a classification signature for non-small cell lung cancer. *Genes Chromosomes Cancer* **56**, 559–569 (2017).
20. Yadav, S. & Shukla, S. Analysis of k-fold cross-validation over hold-out validation on colossal datasets for quality classification. in *2016 IEEE 6th International Conference on Advanced Computing (IACC)* 78–83 (IEEE, 2016).
21. Paszke, A. *et al.* Automatic differentiation in PyTorch. *NIPS 2017 Workshop on Autodiff* (2017).
22. Streck, A. Input dataset for CNSistent integration and feature extraction from somatic copy number profiles. Zenodo: https://zenodo.org/records/14677713 (2024).
23. Streck, A. Processed data for ‘CNSistent integration and feature extraction from somatic copy number profiles’. Zenodo: https://doi.org/10.5281/zenodo.15620292 (2025).
24. Streck, A. Deep Learning code for ‘CNSistent integration and feature extraction from somatic copy number profiles’. Zenodo: https://doi.org/10.5281/zenodo.14546762 (2024).
25. DOME Registry. DOME-ML. https://registry.dome-ml.org/review/59bcqzam2c. (2024).
26. Van Loo, P. *et al.* Allele-specific copy number analysis of tumors. *Proceedings of the National Academy of Sciences* **107**, 16910–16915 (2010).
27. Sondka, Z. *et al.* The COSMIC Cancer Gene Census: describing genetic dysfunction across all human cancers. *Nat. Rev. Cancer* **18**, 696–705 (2018).
28. Lander, E. S. *et al.* Initial sequencing and analysis of the human genome. *Nature* **409**, 860–921 (2001).
29. ICGC/TCGA Pan-Cancer Analysis of Whole Genomes Consortium. Pan-cancer analysis of whole genomes. *Nature* **578**, 82–93 (2020).
30. Boumahdi, S. *et al.* SOX2 controls tumour initiation and cancer stem-cell functions in squamous-cell carcinoma. *Nature* **511**, 246–250 (2014).
31. Talevich, E., Shain, A. H., Botton, T. & Bastian, B. C. CNVkit: Genome-Wide Copy Number Detection and Visualization from Targeted DNA Sequencing. *PLoS Comput. Biol.* **12**, e1004873

(2016).

32. Afyounian, E., Annala, M. & Nykter, M. Segmentum: a tool for copy number analysis of cancer genomes. *BMC Bioinformatics* **18**, 215 (2017).

33. Masood, D. *et al.* Evaluation of somatic copy number variation detection by NGS technologies and bioinformatics tools on a hyper-diploid cancer genome. *Genome Biol.* **25**, 163 (2024).

34. Mermel, C. H. *et al.* GISTIC2.0 facilitates sensitive and confident localization of the targets of focal somatic copy-number alteration in human cancers. *Genome Biol.* **12**, R41 (2011).

35. Shih, J. *et al.* Cancer aneuploidies are shaped primarily by effects on tumor fitness. *Nature* **619**, 793–800 (2023-7).

36. Wuebben, E. L. & Rizzino, A. The dark side of SOX2: cancer - a comprehensive overview. *Oncotarget* **8**, 44917–44943 (2017).

37. Zhang, T., He, R., Xiao, Y. & Geng, Q. Primary squamous cell carcinoma and adenocarcinoma simultaneously occurring in the same lung lobe: a case report and literature review. *Front. Oncol.* **14**, 1402297 (2024).

Supplementary Figures 1-9

Click here to access/download
Supplementary Material
Supplementary Figures CNSistent.pdf

Supplementary Table 1

Click here to access/download
Supplementary Material
supplementary_table_1.xlsx

Supplementary Table 2

Click here to access/download
Supplementary Material
supplementary_table_2.xlsx

Supplementary Table 3

Click here to access/download
Supplementary Material
supplementary_table_3.xlsx

Click here to access/download;Personal Cover;20250608_CNSistent_Revision_CoverLetter_rfs.pdf

University of Cologne
Medical Faculty

ICCB • https://iccb-cologne.org • @rfschwarz

Institute for Computational Cancer Biology

Prof. Dr Roland Schwarz

Phone: +49 221 478 51465

roland.schwarz@iccb-cologne.org
https://iccb-cologne.org

GigaScience
Editorial Board
Qing Lan

Cologne, 09/06/2025

Dear members of the editorial board, dear Mr. Lan,

Please find attached the revised version of our manuscript titled: “CNSistent integration and feature extraction from somatic copy number profiles”.

We would like to thank all the reviewers for their thorough and insightful analysis of our manuscript. Each of the reviews focused on a different aspect of the paper, with Reviewer #1 focusing on detection of recurrent SCNAs, loci detection, and overall analytical methods, Reviewer #2 focusing on the machine learning model and improved validation against other methods, and Reviewer #3 focusing on the toolset itself, applications, and internal algorithms and their description.

With the help of these comments we have updated our manuscript thoroughly and believe it to be in a much better state. The most important changes are:

1. We have removed the Integrated Gradients method and replaced it with i) a simple peak detection method and ii) a statistical test for differentially copy number-altered genes in LUAD/LUSC, in line with the request of Reviewer 1.
2. We have added additional models to the classification task (Random Forest, Elastic Net, and DNN3 and CNN as described by Attique et al.) and investigated the effect of segmentation size on these models, as requested by Reviewer 1. We show that our model outperforms all other models and that CNSistent pre-processing brings significant accuracy improvement across segmentation sizes, as requested by Revierer 1 and 2.
3. We have updated the method description to clarify terminology and the description of the segmentation process. The related Figure 1 has also been updated, in line with the requests of Reviewers 2 and 3. All figures have been updated for increased graphical clarity.

In addition, we have edited several sections for clarity and brevity as requested by Reviewer 3.

We feel that the paper is much stronger now and hope that you will agree that it will make a great contribution to *GigaScience*. The revised manuscript version is accompanied by a point-by-point response in PDF format with additional Figures for the reviewers’ convenience. Our responses and all updated text in the manuscript is marked in blue.

Yours sincerely,

Roland Schwarz
2025.06.09
11:22:45 +02'00'

Roland Schwarz

Revision - Responses to Reviewers
Click here to access/download;Personal Cover;2025_03_18 Reviews CNSistent.pdf

Reviewer #1:

This is a well-written paper that aims to develop a tool that can integrate SCNA from large datasets possibly generated using different platforms to identify alteration patterns that are often undetected in smaller data subsets. Authors have used CNN-based method for integrating the data, extracting features and predicting cancer types from SCNA profiles. The tool has the potential to significantly simplify the integration and analysis of large scale SCNA studies. However, some (hopefully addressable) weaknesses are noted:

1. The choice of a classification task as the (only) way to evaluate the proposed method is questioned. I would argue that the most important use of SCNA detection is in support of mechanistic investigations, by identifying novel candidate loci likely to harbor tumor suppressors (copy losses) and oncogenes (copy gains). This type of analysis is hardly mentioned in the manuscript, and it is not clear how well the proposed tool would support it. I surmise it can, but the authors should discuss (and present results about) it.

Response: We agree with the reviewer that a classification task is not the only and arguably not the ideal way of demonstrating the power of CNSistent. CNSistent will prove useful every time CN profiles have to be integrated between samples from the same patient, between patients or between cohorts. Peak detection certainly is one such example. Since GISTIC uses its own internal integration method for CN profiles, it is not ideally combined with CNsistent.

To follow the reviewer's suggestion, we thus implemented our own simple peak detection algorithm, described in the Methods section Peak Detection: "*To find regions of interest in the samples, CNSistent provides the peak score (PS), which shows how much each bin differs from its neighbours. Have an aggregated sample* $S = (s_1, \cdots, s_n)$ *we set the boundary values* $s_0 = s_1$, $s_{n+1} = s_n$ *and calculate* $\forall i \in (1, \cdots, n)$: $PS(S, i) = (s_i - s_{i-1}) - (s_{i+1} - s_i)$. *This score will be positive for segments higher than their neighbours, negative for those lower and close to zero for segments with monotonous behaviour. We therefore use the PS to detect the highest and lowest values, which show the locations of most abrupt change in CN accumulation. Note that for meaningful calculation this requires that the segments are connected to each other and about the same size.*"

To illustrate the flexibility of CNSistent we applied this simple algorithm to the LUSC samples with varying segmentation sizes from 500 KB to 20 MB. We observe that the top 3 peaks vary between segment sizes, with chr3 being detected mostly in big segments due to a wide slope on the q-arm, while chr8 is being mostly detected in middle sizes due to amplification at the end of the p-arm (new Fig. 4A). We reproduce the Figure here for the reviewer's convenience:

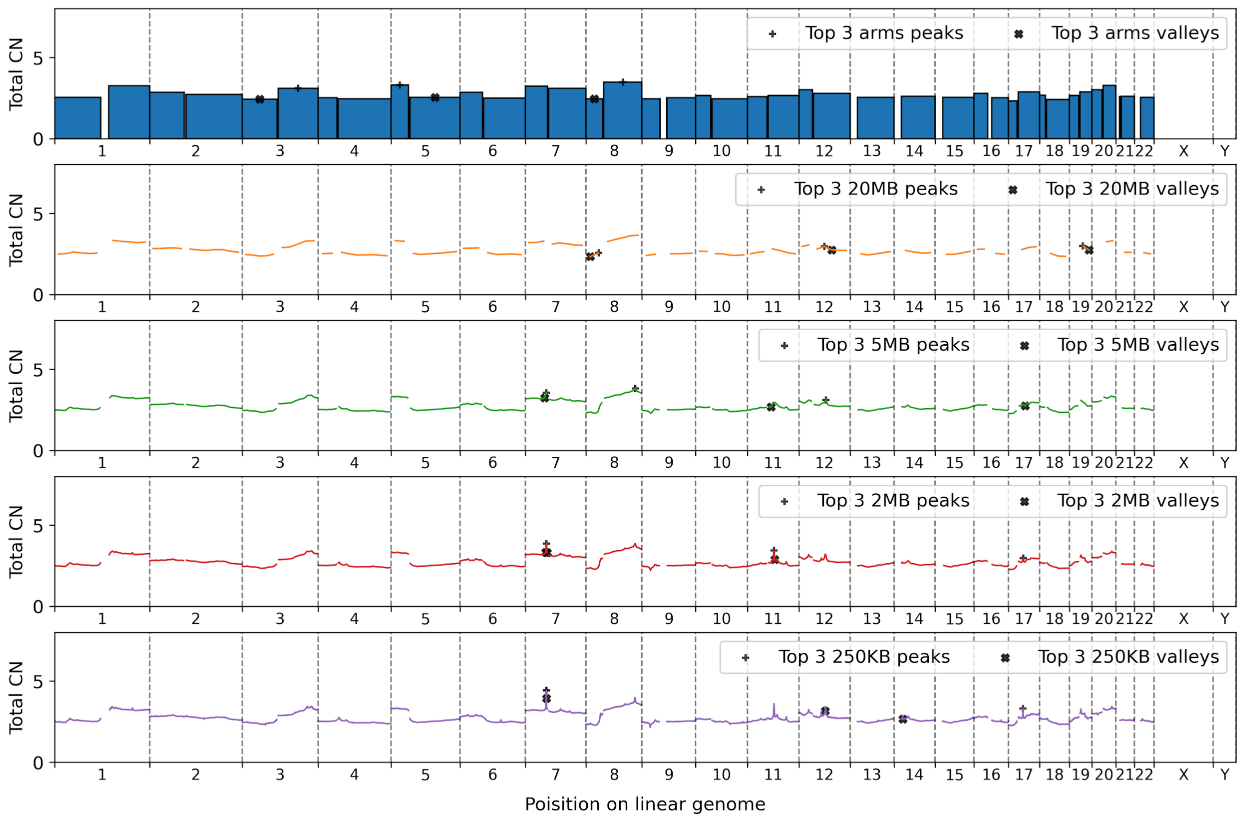

Based on the reviewer comments, we have also removed the Integrated Gradients method in favour of a statistical test in the Results, which reads: *"To systematically determine which genes differ significantly in their CNs between LUAD and LUSC, we conducted a Mann-Whitney U-Test with Benjamini-Hochberg correction on the mean CNs of the COSMIC genes. Out of 722 genes, 599 had adjusted p-value below 0.05. The top 5 most copy number altered genes were all on the q-arm of chr3 (Fig. 4D). All these had the adjusted p-value below* 10^{-169}*, with SOX2*[30] *being the most significant at* $p \approx 10^{-187}$*. The SOX2 gene also has the highest mean CN of 7.56."*

Figure 4D here for reviewer's reference:

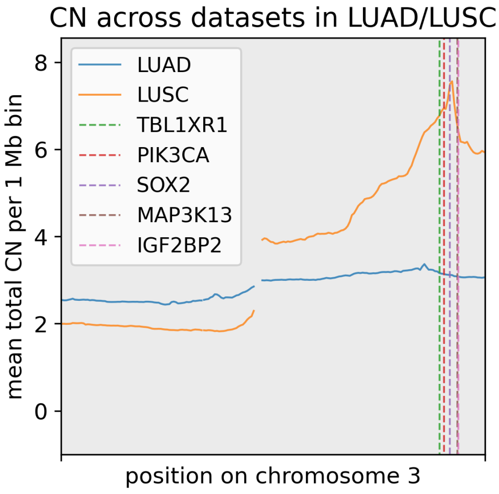

To further illustrate the broad applicability of CNSistent we now provide phylogenetic inference from somatic copy number profiles as an additional example. To this end we leveraged samples from the TRACERx cohort as described together with our phylogenetic inference tool MEDICC2 (cite Kaufmann et al. 2022). Before any phylogenetic analysis can be carried out CN profiles from the same patient have to undergo joint segmentation to make them comparable by an evolutionary model. Figure 4E now shows the CN profiles from TRACERx case CRUK0001 aligned with CNSistent as well as the phylogenetic tree inferred by MEDICC2. We provide the Figure 4E here for reviewer's reference:

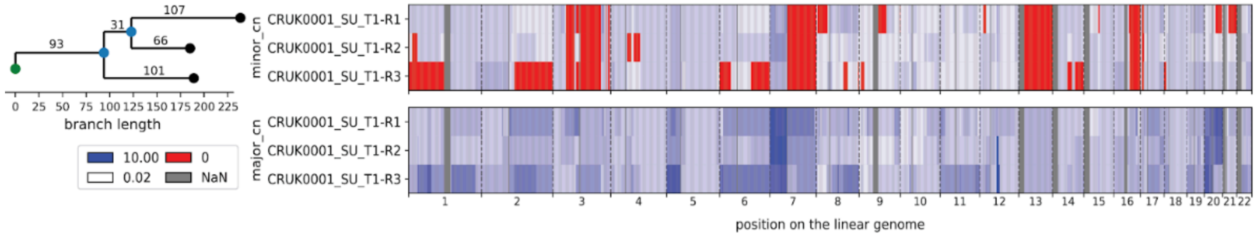

2. If we were to focus on the task of recurrent SCNA detection, then meta-analysis approaches (where separate analyses are performed on each of the datasets, and only the results are integrated) would need to be considered as an alternative to the approach here proposed (e.g., application of GISTIC to each of PCAWG, TCGA, TRACERx separately, followed by meta-analysis integration of the results). I am not saying meta-analysis would be superior, but the authors should discuss it, and possibly evaluate it.

Response: We agree with the reviewer that meta-analysis techniques can be an alternative to CNSistent in some situations. For example, simple gene-level aggregations of CN states might also be possible with ad-hoc implementations. However, for larger segment sizes or as soon as whole-genome CN profiles are considered, technical differences between samples, patients or cohorts, for example with respect to missing data or blacklisted regions will require algorithmic

design decisions that are not trivial. CNSistent provides such tools to enable reproducible processing of large cohorts in a unified manner.

Unfortunately, a direct comparison with GISTIC is not possible, since GISTIC uses its own internal integration strategy. However, as described in response to this reviewer's comment #1, we have implemented our own simple peak detection algorithm to illustrate the use of CNSistent for this purpose and would refer the reviewer to the above comment for details. We have also added a paragraph in the Discussion to clarify this point. It now reads:

"*On the analysis side, there are many well known tools for detection of regions of interest, in particular GISTIC[34] and BISCUT[35], which take SCNA profiles and combine them, however, this is done internally by the tool and not accessible or controllable by the user*."

To demonstrate the increase in power in combining many patients or cohorts we show on the task of NSCLC classification that training on TRACERx and applying the results to PCAWG provides better results than training on PCAWG itself. To make this clearer in text, we have changed the Methods to the following:

"To demonstrate the potential of integration using CNSistent across different datasets. For the binary lung cancer classification task we have trained the models on one dataset and tested on another (Fig. 3E). We see that the results transfer well, with up to 93.90% accuracy for the TRACERx model applied to PCAWG. We also see that compared to self-training, the models trained on bigger sets (TRACERx, TCGA) outperform self-training on the small PCAWG model. Likewise, training on TRACERx slightly outperforms self-training on TCGA. The 5-fold cross-validation accuracy on the combined dataset was 92.12%, considerably improving on the previous result of 84% in Qui et al.(Qiu et al. 2017)."

3. The reported metrics to quantify the quality of the integration are insufficient to assess the results. There is some lack of clarity about the classification accuracy results reported, since it is not clear whether all the components of the model building were adequately brought into the cross-validation (or train/test) loop. More specifically, when reporting the accuracy of the cancer type classification, it is reported that 1 megabase segmentation yields the best results. It is not clear if this size selection was performed within the train set only (and/or within the CV loop) or across the entire dataset. If the latter, this may significantly affect the accuracy results, which could not be deemed (unbiased) "test set" results. This should be clarified, and if the segment size selection was indeed performed outside the train/test split, accuracy measures should be computed again by performing the segment size selection properly (which of course it would mean a potentially different size would be selected for each of the folds).

Response: We thank the reviewer for raising this point, which we hopefully can clarify. We here aimed to compare the different segmentation strategies, rather than to select the best model. The split into the 5 folds is the same for each segmentation strategy and the accuracy has been averaged across the 5 folds in order to best demonstrate how well a method would perform on

such a segmentation. We are aware that using this method makes the nomenclature somewhat confusing, since the validation accuracy is the mean of the test accuracies. We have aimed to address this by including a new paragraph in the Machine learning subsection:

"*To obtain scores of individual models on the individual datasets, we use 5-fold cross validation, i.e. we split each dataset into 5 groups and always withheld one while training on the other 4. The validation accuracy for each model/size combination is then the mean of the 5 different splits (Yadav and Shukla 2016).*"

We use this validation technique rather than train-test-validation split because i) the dataset is quite small, ii) we mostly aim to compare between different options and averaging training results prevents possibility of favorable selection of validation set for a particular method.

The segmentation strategies are pre-selected and tested independently, i.e. there's no segment size selection during the train/test process, we just report the results of all the options. We tried to make the segment size selection process clearer with the following paragraph in Results:

"We next set out to explore the effects of different segmentation strategies on the cancer classification task (see Methods). We processed the data using the following segmentation strategies: (i) fixed-size segments of 20 Mb, 10Mb, 5 Mb, 3 Mb, 2 Mb, 1 Mb, 500 Kb, 250 Kb, 100 Kb size; (ii) Whole chromosomes, chromosome arms; (iii) Gene-level CN values based on the ENSEMBL and COSMIC gene sets; and (iv) breakpoint merging using distance thresholds of 1 Mb, 500 Kb, and 250 Kb. The segment sizes roughly cover the ranges used by other authors for feature discovery(Drews et al. 2022)"

4. Comparisons with other methods: The authors only compare their method to random forest (RF). Related to the previous point: I presume the RF model used the segment size that was optimized for the CNN model (i.e., 1Mb). If this is the case, it would be an unfair comparison, since RF might favor a different size. Also, additional classifiers should be evaluated (e.g., Elastic Net, SVM, etc.).

Response: We have now calculated the performance of the RF classifier across all segment sizes and added an additional linear classifier with Elastic Net regularization for comparison. We decided against inclusion of a SVM as the performance of this kernel-based classifier would strongly depend on the kernel and hyperparameters used.

While our original neural network was very close to the architecture used by Attique et al., it included an additional linear layer. We therefore now reconstructed the DNN3 and CNN of Attique et al. to the best of our ability based on their manuscript and included our model as an additional one, called CNN+.

The following plot has been included in the manuscript as new Figure 3B:

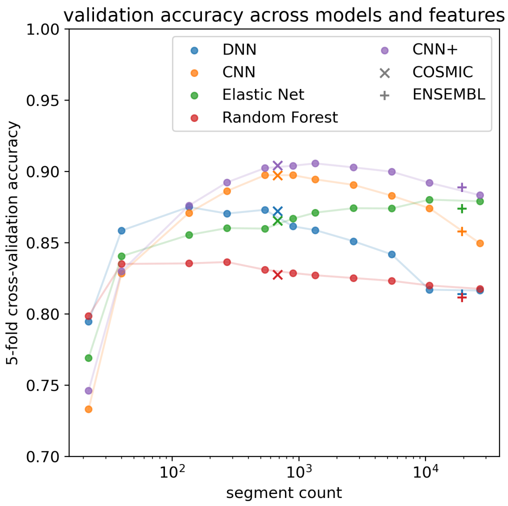

The corresponding text in the results section reads:

"We compared the DNN3 and CNN architectures of Attique et al., Random Forest, Elastic Net, and our extension of the CNN architecture–CNN+ (Fig. 3B)–across decreasing segment sizes. On whole chromosomes the most performing models reached a validation accuracy of ~80% and considering the arms separately reached almost ~86% on the DNN architecture. Increasing the resolution improves accuracy of the two convolutional models, which peak in the region of around 1000 segments. The best validation (mean of 5 folds) accuracy of 90.60% was achieved with the CNN+ at 1 Mb segments, which we used for the subsequent tasks, however sizes from 5 Mb to 250 Kb all had validation accuracy above 90%, and we would therefore consider all of them to be suitable for any further analyses.

The RF and DNN models however peak around 200 segments and increasing the resolution further decreases the validation performance, likely due to overfitting. The only architecture that improved monotonously was ENet, where the penalty regularization seemed to prevent overfitting, however from 20 Mb onwards it always underperforms compared to the CNN+. Comparing the full segmentation with the COSMIC and ENSEMBL gene sets we saw that taking only the CNs for genes performs equivalently to creating a segmentation with a similar number of features."

5. There is no sufficient discussion of existing tools/methods. This should be corrected (see also my comment about meta-analysis approaches).

Response: To better situate CNSistent in the field and to provide a better overview of existing alternative tools, we have added the following paragraph to the Discussion:

There are tools available that call CNs from various sequencing data and provide related visualizations in Python, e.g. CNVKit[31] or Segmentum[32], with many more outside Python, with comparison studies done e.g. by Masood et al.[33]. On the analysis side, there are many well known tools for detection of regions of interest, in particular GISTIC[34] and BISCUT[35], which take

SCNA profiles and combine them, however, this is done internally by the tool and not accessible or controllable by the user. To the best of our knowledge the only tool for integrative analysis of SCNA profiles is the web-based CNApp[14], which shares some of the functionality with CNSistent, in particular re-segmentation and calculation of profile statistics. However, CNApp is designed for analysis within a web dashboard, while CNSistent serves as a tool for the integration of data before application of downstream tools. We did not fully compare the tooling to CNApp as the hosting was not available at the time of writing.

6. Metadata effects: Age influences the copy number alterations. The authors don't consider age or any other metadata and their implication in the classification task.

Response: We thank the reviewer for pointing this out. To evaluate if age might act as a confounding in our cancer type classification task we investigated the predictive power of age on the cancer types considered. A simple Linear classifier on the top 6 types showed a test accuracy of 0.322, indicating that age alone is not a strong predictor of cancer type and thus is unlikely to confound our classification results.

For reference, please also see this Reviewer Figure 1:

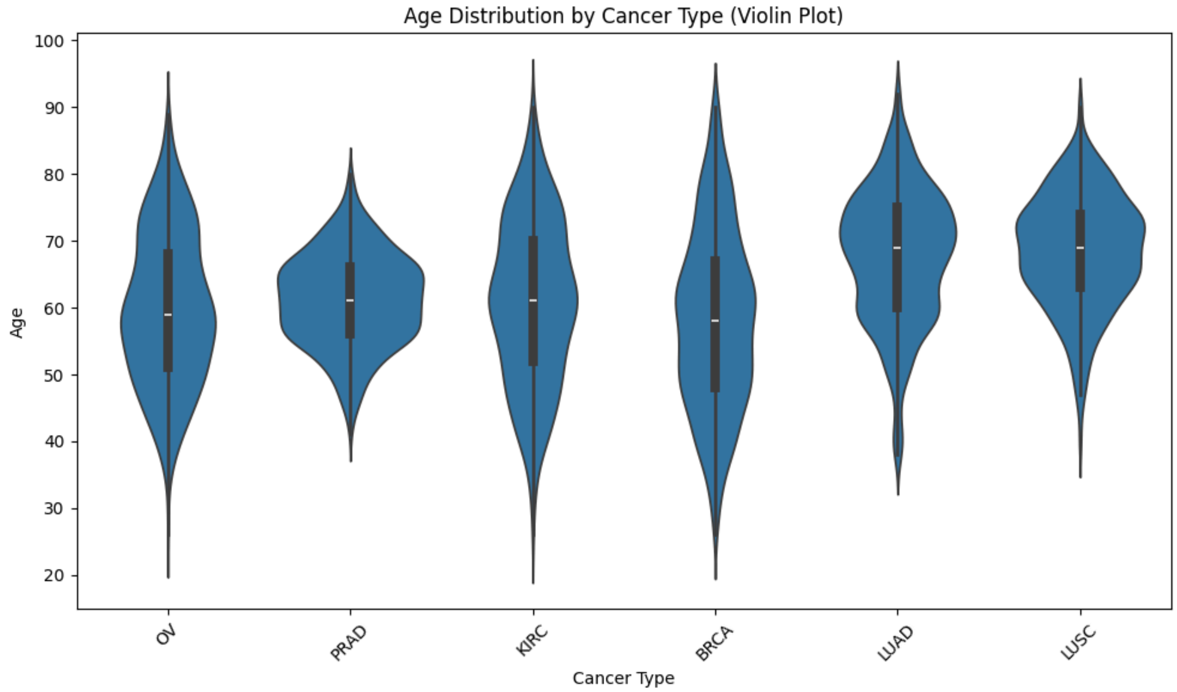

Unfortunately, we were not able to obtain age values for ~10% of the samples. In the interest of manuscript length, we have decided not to include this analysis in the text but naturally would be happy to do so if the reviewer feels strongly about this.

7. Run time statistics and user requirement: While the authors report runtime curves per command (S Fig 6), it is difficult to translate this to total runtime. It would be useful if runtime for

the entire training of a model were reported. Additionally, if available, comparison of run time stats with the established model that they cite would be useful.

Response: We have reproduced two of the existing architectures and compared the times to our model, Random Forest and Elastic Net. The following figure has been added as Supplementary Figure 7:

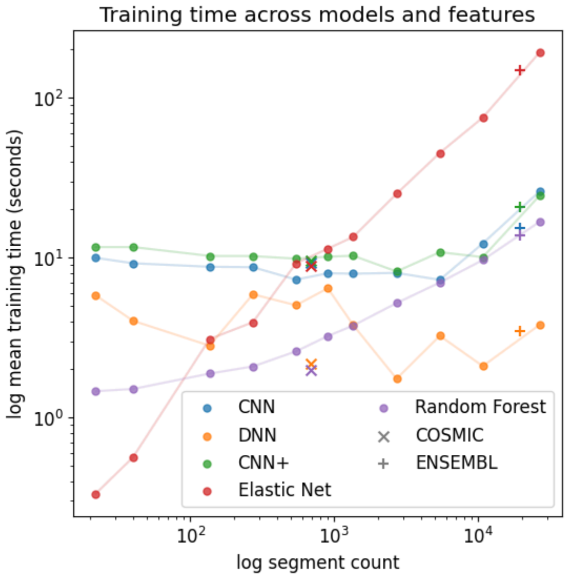

8. IG-based explanation. I found this section sort of perfunctory, not sufficiently justified, and adding little to the manuscript. IG is computationally expensive, and it does not provide any way to statistically quantify the found associations. Simpler methods, such as testing for association between SCNA occurrence and cancer type should be evaluated and compared to.

Response: We originally used IGs as a preliminary exploration of points of interests, but we agree with the sentiment concerning the computational costs. We have now completely removed the IG section and instead replaced it with a simpler section focused on a statistical test for association between cancer type and the prevalence of copy number changes.

Briefly, we applied our Peak Score to 1 Mb segments. This detected the peak in the vicinity of SOX2 on chromosome 3 and the focal amplification at the p-arm of chromosome 11 near the centromere, matching the results of the application of IGs to Segments.

We then conducted Mann-Whitney U-Test on the COSMIC gene sets comparing the LUAD-LUSC sample sets. The set of the 5 most statistically significant differences between the genes and the set of 5 genes with the highest IG score have both on 3 of the genes: SOX2, PIK3CA, and TBL1XR1.

We therefore conclude that these much simpler and more explainable methods suit as a good replacement of our previous IG-based analysis.

For details, please see the updated section: Identifying commonly altered regions and outliers .

9. Model selection: No adequate justification of why they picked CNN for this task when the referenced paper itself claims the DNN architecture performs better. Not sure but is this because of the varying segment size? Again, this is not clearly stated.
https://pmc.ncbi.nlm.nih.gov/articles/PMC9203194/#tab1

Response: We have attempted to reconstruct the models of Attique et al. as faithfully as we could based on the manuscript, however there were limitations, which we summarized at the end of the Discussion:

"Unfortunately, the comparison of our models to the one from Attique et al[18] *was partially limited by the fact that the authors did neither provide all of the model parameters, nor the model source code, and that the source dataset was no longer available at the time of writing this article."*

In the methods we specify that the Dropout probability and MaxPool kernel size are not declared. Additionally, it was not clear what the layer sizes for DNN5 were. As DNN3 has already been overfitting in our implementation, we have not included the DNN5. Also the declared test accuracies of DNN3 and DNN5 were 91% and 92% respectively, therefore we have not felt that there was a meaningful difference.

Reviewer #2:

The paper introduces a python package for imputation, filtering, segmentation, feature extraction and visualisation of CNA profiles. It explains some of the elements of the package, and then demonstrates how data from multiple cohorts can be processed and combined using the package preprocessing pipeline. The authors then use processed data from 3 different cohorts to perform cancer type prediction using a CNN. From this, they get an interesting result to find a biomarker that differentiates two different lung cancers. Throughout, they show visualisations using their package. The package itself seems well documented and designed to be used. There is some clarification required in the methods section specifically around the CNN training and the models therein. There is also one major question of whether all the preprocessing steps are actually required for the downstream CNN analysis. Overall, however, this is a well written manuscript, providing a useful
software tool for further analysis of CNA data.

Major comments:
- CNN section- how are the segments decided- is it based on all the training data, or just data in a batch?

The segmentation strategies are pre-selected and tested independently, i.e. there's no segment size selection during the train/test process, we just report the results of all the options. We tried

to make the segment size selection process clearer with the following paragraph in Results:

"We next set out to explore the effects of different segmentation strategies on the cancer classification task (see Methods). We processed the data using the following segmentation strategies: (i) fixed-size segments of 20 Mb, 10Mb, 5 Mb, 3 Mb, 2 Mb, 1 Mb, 500 Kb, 250 Kb, 100 Kb size; (ii) Whole chromosomes, chromosome arms; (iii) Gene-level CN values based on the ENSEMBL and COSMIC gene sets; and (iv) breakpoint merging using distance thresholds of 1 Mb, 500 Kb, and 250 Kb. The segment sizes roughly cover the ranges used by other authors for feature discovery(Drews et al. 2022)"

- Throughout the results pertaining to figure 3A-C, you call it test accuracy- to be clear is this is based on your CV hold outs? This should be reworded everywhere to reflect this. As cross validation indicates, this is not a test set and is a validation set- which is also the way you use it.

We thank the reviewer for raising this point, which was also raised by Reviewer 1. In essence, we aimed to compare the different segmentation strategies, rather than to select the best model. The split into the 5 folds is the same for each segmentation strategy and the accuracy has been averaged across the 5 folds in order to best demonstrate how well a method would perform on such a segmentation. We are aware that using this method makes the nomenclature somewhat confusing, since the validation accuracy is the mean of the test accuracies. We have aimed to address this by including a new Methods paragraph in the text as shown below:

"*To obtain scores of individual models on the individual datasets, we use 5-fold cross validation, i.e. we split each dataset into 5 groups and always withheld one while training on the other 4. The validation accuracy for each model/size combination is then the mean of the 5 different splits (Yadav and Shukla 2016)."*

- Regarding the above, you have a comment saying: "the best test accuracy without cross-validation was 92.34%". Could you please clarify what you mean by this. Only in the CNN section do you describe your training approach, which does not mention a test or separate validation set.

Response: We hope the validation approach has been made clearer by the above paragraph. The test accuracy of 92.34% comes from the fact that Attique et al. did not conduct validation, therefore we can only compare to their test scores. However, we believe the validation score to be a more informative metric of classification performance and therefore chose to work with that number instead. We have updated the Results to reflect this as follows:

"The best test accuracy (maximum of 5 folds) was 92.42% on 1 Mb segments with the CNN+ model. This is slightly above the best test accuracy of Attique et al. (92%)."

This is further discussed in the Discussion:

“Validation on a hold-out set or cross-validation has not been conducted by the authors, we therefore only performed our comparisons on the test accuracies.“

- It reads slightly unclearly- you have a section called "model transfer", but are you training 3 different models- one per dataset? You only have one figure for training results which suggests one dataset, but then you have this section called model transfer?

Response: We agree with the reviewer that our terminology might have been confusing and have removed the term “transfer” altogether. We have indeed trained 3 different models, each on one dataset and applied it independently to the remaining two, creating 6 combinations, where one dataset is the training set and the other is the validation set. We have then compared these to the results of the 5-fold cross validation on each individual dataset. We have generally scaled this section back in line with the decrease of focus on deep learning and tried to express the process as clearly as possible in the Results:

“To demonstrate the potential of integration using CNSistent across different datasets we used the NSCLC classification task, training the models on one dataset and validating on another (Fig. 3E). We see that the accuracies of models trained on a different dataset match or sometimes even outperform models trained and validated on the same dataset, with up to 91.46% accuracy for the TRACERx model applied to PCAWG. We also see that compared to self-training, the models trained on bigger sets (TRACERx, TCGA) outperform self-training on the small PCAWG model. Likewise, training on TRACERx slightly outperforms self-training on TCGA. The 5-fold cross-validation accuracy on the combined dataset was 92.73%, considerably improving on the previous result of 84% in Qui et al. When training the models individually, we obtain only 91.21% mean validation accuracy, showing that combining the datasets leads to a (1.52%) improvement.”

- Re all the above, please dedicate a small subsection in methods making this clearer. Are there dedicated test sets? If your main results are for aggregated data, then what are you testing on to ensure generalisability? What is the point of training the 3 different models on 3 different datasets? Perhaps it would make more sense to hold one dataset out as your test set. In some ways, that is what the model transfer is showing, but it would be less confusing to clarify that aim instead of suddenly introducing 3 models.

We have now updated the Methods section to accommodate the comments by the Reviewer as indicated in detail in the answers above, to which we would kindly refer you here.

Regarding the question about the three models, we use this to test whether classifiers can be transferred from one cohort to another, similar to a train/validation split, i.e. we hold out the remaining two cohorts and train only on one cohort with the remaining two used to obtain two validation scores. We hope that this is also clearer from the text quoted above.

- If the CNN architecture is essentially the same as in Attique et. al., the performance is basically the same and they use only CNs a gene locations- how does this demonstrate that the

preprocessing from CNSistent is necessary or advantageous for this task? Maybe having a result which combines CN calls naively over gene locations and comparing to this across the aggregate datasets would be a good way of comparing? I.e showing that preproccessing does offer an advantage when combining different datasets together? Also because this is what you argue in your abstract. For this analysis you would have to make sure you also compare across the same samples to differentiate between filtering/other preprocessing steps.

Response: We agree with the reviewer that aggregation or meta-analysis techniques can be an alternative to CNSistent in some situations. For example, simple gene-level aggregations of CN states might also be possible with ad-hoc implementations. However, for larger segment sizes or as soon as whole-genome CN profiles are considered, technical differences between samples, patients or cohorts, for example with respect to missing data or blacklisted regions will require algorithmic design decisions that are not trivial. CNSistent provides such tools to enable reproducible processing of large cohorts in a unified manner.

To demonstrate the increase in power in combining many patients or cohorts we show on the task of NSCLC classification that training on TRACERx and applying the results to PCAWG provides better results than training on PCAWG itself, as discussed in the paragraph above.

Furthermore, we have added a comparison of filtered (pre-processed) and non-filtered (all available) sample sets in Figure 3C, which shows clear and consistent improvement after the filtering process:

Figure 3C reproduced here:

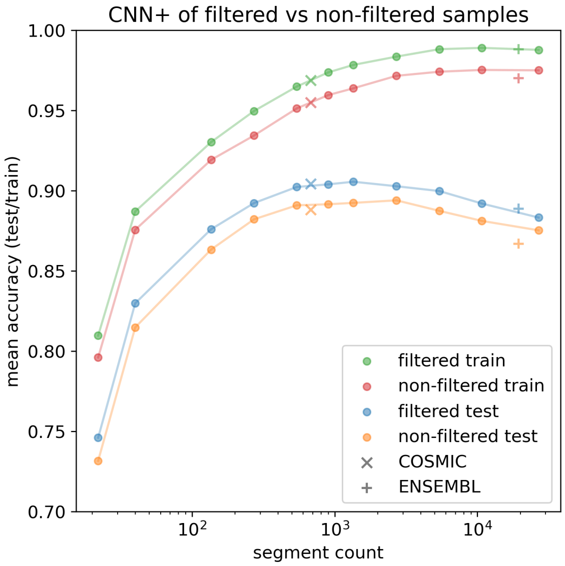

- In Figure 3I, you say "notice the similarity of chromosome 3 pattern for the correctly classified LUSC samples (red) and the misclassified ones (orange)". This is confusing because the orange and red are not similar. In fact for this whole section, it seems that figure 3I does not align with what you are saying?

Response: We apologise for this mistake on our part. There was indeed an error in Figure 3. However, in Response to Reviewer 1 comment #8, we have now removed the IG analysis from the paper and replaced it with a statistical association test. This also removes Figure 3I. We reproduce the new Results paragraph and corresponding Figure 4C here for your convenience:

“We then calculated the outlier score between LUAD and LUSC and used the kneepoint detection to find an outlier threshold (Supp. Fig. 8), finding 3 LUAD samples with LUSC-like pattern (amplification of SOX2) and 54 LUSC samples with neutral LUAD-like pattern (Fig. 4C). We also observed that the majority of these samples (58.75%) came from the TCGA dataset, while the TRACERx dataset had the least outliers (15.79%).”

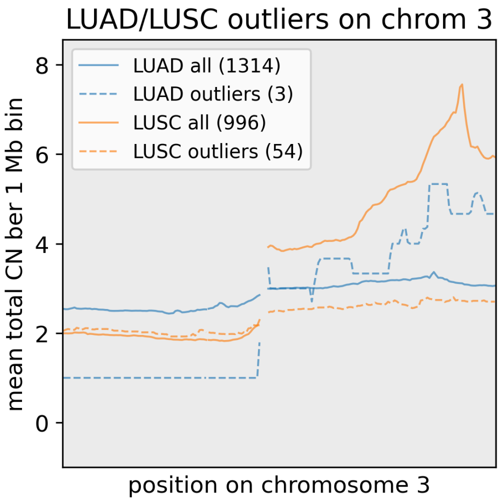

Minor comments/errors:

- Clarification on why CNSistent needs a reference genome if it's dealing with segments? How is this information used- is it just for the known gaps?

Response: The reference genome is needed to know the expected chromosome lengths for the imputation as described in the Segment Imputation subsection “*(ii) CNSistent extends the first and last segment of each chromosome to the chromosome boundaries*”. CNSistent also provides built-in segmentation based on cytobands (listed as option in Consistent Segmentation subsection), whose locations are likewise taken from the reference.

- Your caption of Supplementary Figure 1 has a typo about a breakpoint at 16 instead of 14.

Response: Thank you for noticing, we have fixed the typo.

- You do not explain how you use the knee pt to filter (i.e is it samples above/below the knee pt.)

Response: “We used individual thresholds to filter the samples” changed to *“We used individual thresholds to remove samples whose values were strictly smaller than the threshold”*

- Your CNN graphic is difficult to interpret and non-standard.

Response: We have updated the figure to more closely match the standard visual scheme for CNNs and updated the figure caption:

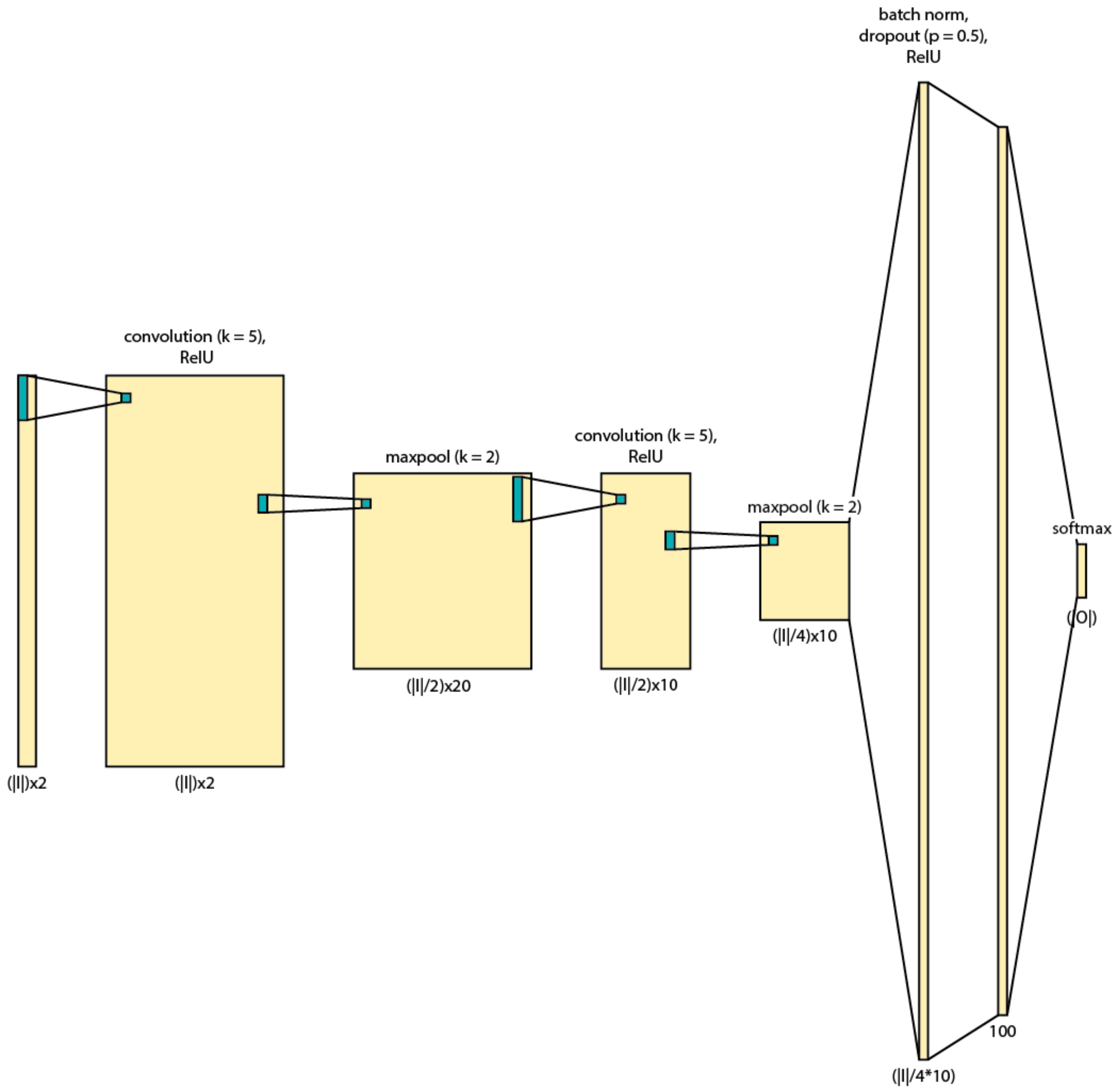

Supplementary Figure 3: The CNN+ model of auto-scaling 1D convolutional neural network. The input layer I has size |I| and the output layer O has the size |O|, corresponding to the number of classes. The example is visualized for the case of 6-type classification (|O|=6) on filtered chromosome arms (|I|=40).

- CNN section should clarify at the beginning what the input is and what the output is (i.e a prediction that a sample belongs to a particular cancer type) before explaining the architectural details.

Response: We have added the following to the Machine learning subsection to clarify: *"In this task, each binned sample as illustrated in Fig. 1 represents one feature vector. The output probability that a sample belongs to each cancer class under consideration."*

- Even though you control for class imbalance, some cancer types are so poorly represented it is unlikely a CNN could learn that, you do kind of mention this in the discussion, but maybe some sort of minimum threshold for inclusion would make sense.

Response: Thank you for the suggestion, we agree with the point and we have hence limited the classification to only classes with at least 100 samples. The supplementary figure has been updated to reflect this:

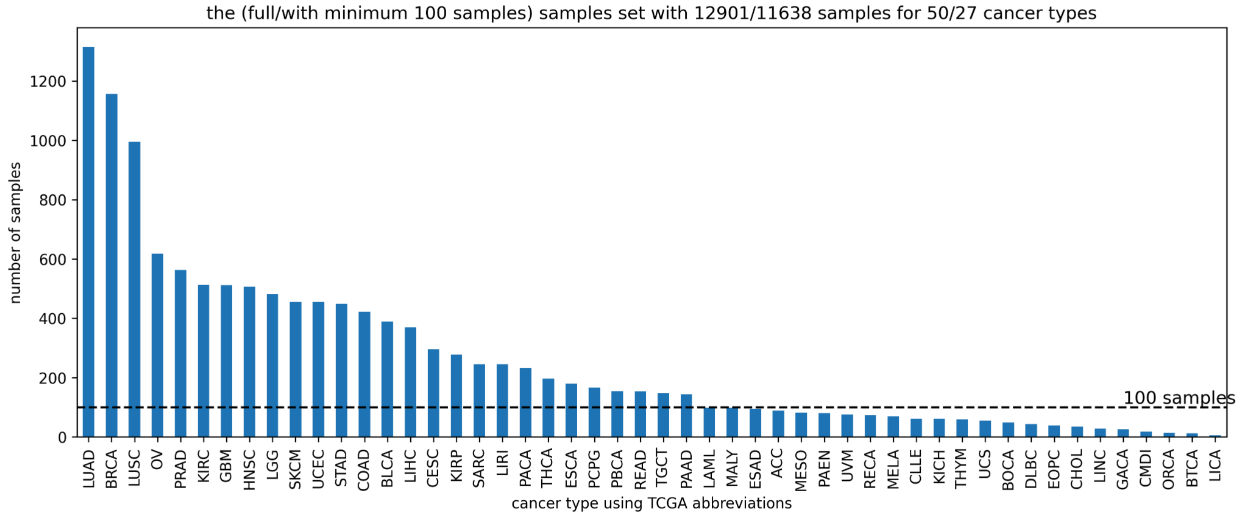

The result Figure 3D has been updated accordingly:

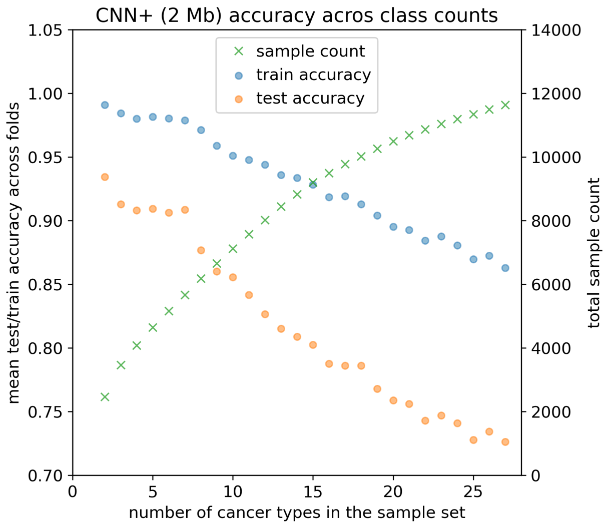

- For Fig2D you refer to it as GND, but the axes/title says hemizygosity-are these things equivalent? E.g could have 3-3, low hemizygosity but not diploid? Or if it's aggregated across the whole genome its assumed equivalent?

Response: Thank you for spotting the discrepancy. The x-axis label and title were supposed to read GnD, which we have now corrected.

- There is a grammatical error "Runtimes decreased in a near-linearly with the number of compute cores"

Response: Thank you for spotting the mistake, we have corrected it to: *"Runtime decreased in a near-linear fashion with the number of compute cores available."*

- You make a comment that "We therefore suspect some TCGA lung cancers might be cases of co-occurring adeno and squamous carcinomas." This is a possibility but given pleiotropy of many phenotypes- it may also be that the biomarker is not always unique to squamous carcinomas.

Response: We have updated our analysis using the outlier detection method where the SOX2 amplification shows a considerably clearer pattern (see below), however it is still a hypothesis and we cannot confirm or reject it from copy numbers alone.

New Figure 4C:

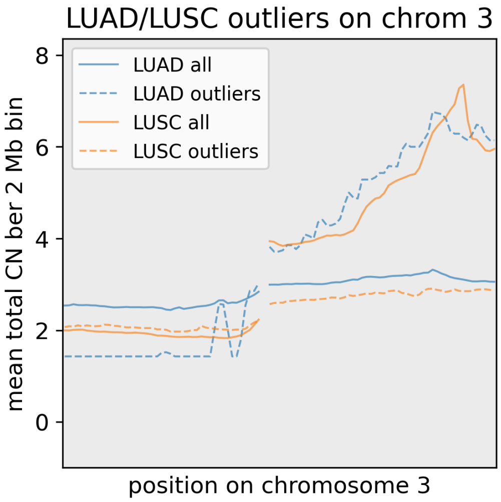

Suggestions/Nice to haves:

- Maybe make it clearer inside the paper what visualisations come with CNSistent. Looking at the software documentation, there's obviously a lot of useful visualisations that come with that- and some of them you have used in Figure 3 for e.g.

Response: All of the plots in Figure 4 are now produced by CNSistent. The label also states that explicitly: "*Except for the phylogenetic tree, all plots are produced using CNSistent plotting functions.*"

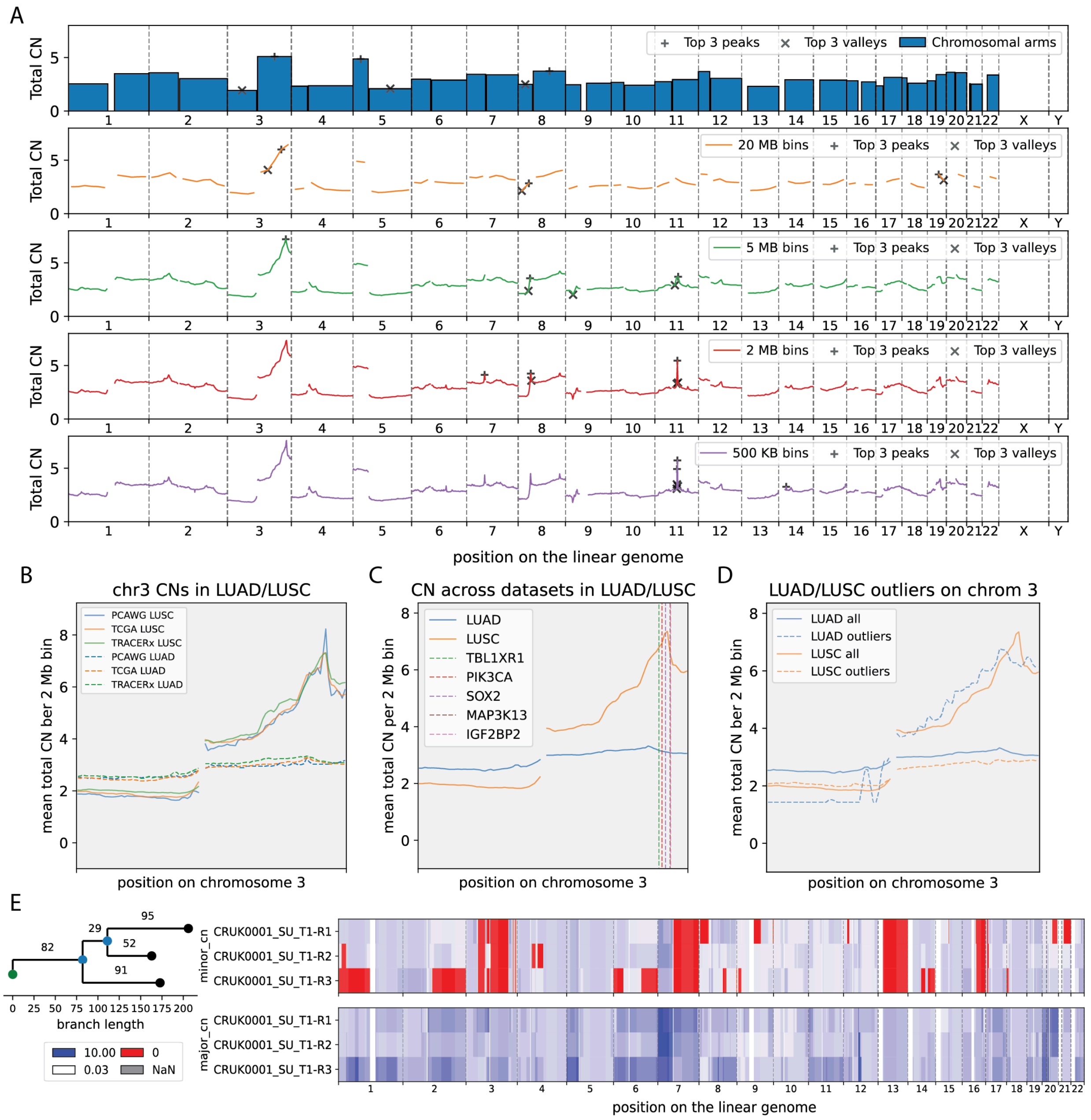

- Given there are more total CN callers, maybe good to mention somewhere how CNSistent would work for total CNs only.

Response: We have added the following sentence to the methods: *"The processing is the same for both allele-specific and total copy numbers, however some of the statistics are limited in the case of copy numbers, as detailed below."*

- You remove profiles that you say are uninformative, could you not include this and then just show how accuracy correlates with no. of break-pts (for e.g). In some ways one might think that

there could be useful information in few alteration profiles- because those alterations might be more upstream/causal.

Response: We hope that the Figure 3C demonstrating the difference between filtered and unfiltered samples demonstrates the benefit of filtering:

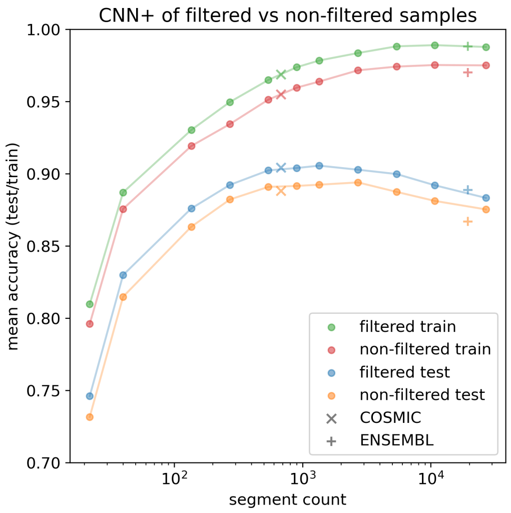

We preferred to display this on our filtering results rather than on the breakpoint count as we don't see breakpoints as we tried to argue for the GnD as an evidence of SCNAs rather than a breakpoint count, as we argued in Fig. 2E and the related text:

"Other authors have used the number of breakpoints[9] as evidence for SCNAs, however we have not observed a clear knee-point in the data (Fig. 2E) and any threshold would therefore be arbitrary."

- The aggregation step could maybe affect downstream analysis. I.e taking the average could introduce CNs that were never called. Even using min/max- this implies a constant copy number in that region, which may lose information- e.g if it is a functional region having two diff CNs across gene might imply non-functionality. Did you explore the effect of aggregation step? Perhaps taking a small enough resolution of segment types would account for this anyway.

Response: We have added a 100 Kb resolution, which is still about 30-times smaller than what a full minimum consistent segmentation would be, however even there we see a trend of flattening training accuracy and decreasing test accuracy, therefore we presume smaller segmentations would not help.

Additionally we compared the Min/Mean/Max strategies for COSMIC and ENSEMBL. The following paragraph has been added to the results:

"Similarly, considering different aggregation strategies for COSMIC and ENSEMBL has not affected the results significantly: for COSMIC the results were Min: 90.94% Mean: 90.25%, Max: 90.05%. For ENSEMBL: Min: 87.44%, Mean: 89.92%, Max: 89.54%."

Reviewer #3:

Streck and Schwarz present a method, CNSintent, for consistent segmentation of copy-number data. The utility of the tool is demonstrated using three large cancer cohorts and a neural network classifier built upon the consistently segmented data. CNSintent can facilitate solving an important biomedical problem: the advanced analysis of copy-number data. The authors are lauded for their excellent Python code and thorough documentation. While the contribution is timely and likely important, there are several areas for improvement.

The manuscript's readability could be better. There are typos, textual errors, and inconsistencies in figure captions, such as incorrect figure references or mismatched values between the text and figures. The "Consistent Segmentation" section is difficult to follow. It is unclear whether this step involves merging pre-existing breakpoints in the data to produce new, longer segments or if larger segments, such as whole chromosomes, are split into smaller, constant-sized segments. The writing suggests that segments are first merged and then split; however, later in the manuscript, they appear to be used separately. In our testing, combining these approaches did not yield meaningful results. Since consistent segmentation is the method's most critical step, we strongly suggest clarifying this section.

Response: We apologise if the workflow was not fully clear. Both merging and splitting are optional. While technically doing both consecutively is possible, i.e. it is possible to first merge the breakpoints and then subdivide newly created regions, this is not very common in practice and we did not use both steps at the same time. We have updated the first paragraph as follows:

"*Segmentation consists of the following 4 steps: (i) define regions of interest (e.g. whole chromosomes, coding genes, etc.), (ii) remove exclusion regions (e.g. telomeric or centromeric regions), (iii) share existing breakpoints between samples and merge them based on a distance threshold, and/or (iv) subdivide the segments into fixed-width bins. Each of the four steps is optional.*"

The Results section now describes the segmentations as follows:

"We next set out to explore the effects of different segmentation strategies on the cancer classification task (see Methods). We processed the data using the following segmentation strategies: (i) fixed-size segments of 20 Mb, 10Mb, 5 Mb, 3 Mb, 2 Mb, 1 Mb, 500 Kb, 250 Kb, 100 Kb size; (ii) Whole chromosomes, chromosome arms; (iii) Gene-level CN values based on the ENSEMBL and COSMIC gene sets; and (iv) breakpoint merging using distance thresholds of 1 Mb, 500 Kb, and 250 Kb. The segment sizes roughly cover the ranges used by other authors for feature discovery (Drews et al. 2022)."

We hope that improves the clarity of the process.

The manuscript is unbalanced in its content, with excessive focus on the tool's application and the discoveries derived from it, rather than on the tool itself. This reduces the clarity of the key

message. We recommend compressing the application section (deep learning in cancer classification) while expanding the tool description with additional explanations.

Response: We thank the Reviewer for this comment and while we would have loved to reduce the application part of the manuscript in favor of a more detailed explanation of the tool itself, both Reviewer 1 and Reviewer 2 asked for additional data analyses, so we had to find a compromise. In line with your request and also Reviewer 1, we have removed the Integrated Gradients Method altogether and replaced it with a more compact subsection of statistical analysis (section title: Identifying commonly altered regions and outliers) instead. We have also removed not strictly necessary parts including the UMAP analysis, and added more general examples of the application of CNSistent instead. We hope that this improves upon the clarity of the main message of the paper. We have also overall streamlined the application section and edited it for brevity, to further improve on this point.

It is also unclear what type of data the authors are using in the cancer classification section. To improve clarity, this information should be explicitly included in the methods section, detailing the sequencing strategy and copy-number tools used for each cohort.

Response: We have added the following to the data availability:

"For TCGA the ASCAT team called the CNs by ASCATv3 from WGS, the TRACERx team used ASCATv2 from WES and PCAWG consortium published CNs obtained as a consensus of 5 different callers (PCAWG Consortium 2020) from WGS."

The methods section would benefit from a more detailed explanation of the CNSintent steps. Both Figure 1 and the text leave some parts unclear, particularly in the "Consistent Segmentation" section. Additionally, methods such as random forest and UMAP are only briefly mentioned in a supplementary figure rather than being described in the methods section. Moving these descriptions to the methods section would improve clarity.

Response: We have now clarified the Methods section and updated Figure 1 as described in the first comment. We have removed the UMAP analysis as we felt it does not provide enough interesting additional information and it is not part of the CNSistent package, in line with your comment #1. Since the Random Forest method is widely used in the field and not specific to the task we consider here, we decided in the interest of space to not provide a technical description of the method. We detail information about which version of the corresponding Python libraries was used in which part of the analysis in the Methods section:

"The splitting is done using the `StratifiedGroupKFold` object from scikit-learn v1.4.1, which was also used for ENet and RF classifiers.

For ENet we used the `SGDClassifier` with log loss and the `elasticnet` penalty. For RF we used `RandomForestClassifier` with default parameters."

Figures are generally clear, but improving color differentiation would be beneficial. For example, in Figure 1, the dark red and dark orange shades are too similar, making them difficult to distinguish. A more optimized color scheme with slightly lighter tones (i.e., increased luminance) would enhance readability.

Response: We agree with the point and we have changed the colors accordingly, as shown below. The dark red and dark orange are now "tab:red" and "tab:purple" respectively. We have also switched all plots to a standard unified palette with more discernible colors. We hope that this improves upon the readability and accessibility of the Figures.

Figure 1A reproduced here:

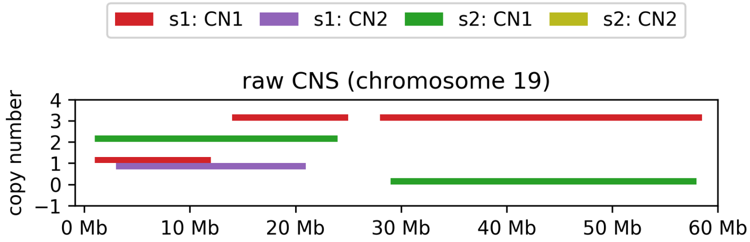

The introduction promotes copy-number signatures; however, these signatures rely on segment lengths and unique breakpoints, which vary between samples. Since this method enforces consistent segmentation and breakpoints across all samples, its applicability to copy-number signatures is unclear. This should be discussed in the Discussion section or removed from the introduction.

We agree with the reviewer that our original intention to promote the use of CN signatures has not been reflected in further sections. CNSistent can generally produce features used for detection of signatures, e.g. breakpoint counts or step sizes, however we have in the end did not develop this to a full extent. Consequently, we have removed mentions of CN signatures from the manuscript altogether.

Out of curiosity: Is it possible to prioritize one type of segmentation over another? For instance, if both WGS and WES data are available, can CNSintent be configured to prioritize WGS calls? Similarly, some tools provide highly precise breakpoint calls that are valuable for detecting fusion genes or rearrangements. In such cases, it would be useful to prioritize these calls and harmonize results from other tools accordingly.

We thank the Reviewer for this suggestion, which is a really interesting one. Unfortunately, currently CNSistent does not support such a weighting of breakpoints. When discussing a potential implementation we realised that there are quite some technical details to be decided upon that would depend from use case to use case and that this is actually a somewhat larger feature that we would like to postpone for the next version of the package.

Terminology Clarifications:

Blacklist, blacklisted regions, gap regions, mask: These terms should be used consistently, particularly since blacklists can be applied at different processing stages. Notably, PCAWG blacklists samples, not regions.

Response: Thank you for the suggestions. To make the text consistent we have used these three exclusive terms:

- **Gaps** refers to the gap regions table as defined by UCSC genome browser. We retained this term without changes.
- **Region exclusion** refers to the process of removing specific regions from downstream analysis. Now defined in methods *"Optionally, exclusion regions can be provided to the pipeline to remove locations in the genome where we expect lower quality of information."* All occurrences of the term blacklisting in this context have been removed.
- **Blacklisting** refers to removal for samples from the PCAWG dataset which were not marked as "whitelisted". We retained this term without changes.

Segmentation: The term is commonly used in CNV analysis to refer to inferring continuous genomic segments from raw read counts or probe intensities. Here, it has a slightly different meaning—computing consistent breakpoints across all samples—so a more explicit definition would be helpful.

Response: Thank you for the comment, we decided to declare the meaning explicitly in the Methods section to avoid any confusion:

"Note that we use the term segmentation to refer to a consistent segmentation between samples, i.e. a set of positions inside each chromosome that split the chromosome into segments."

Breakpoint merging/clustering: If these terms are synonymous, choosing one would improve readability.

Response: Thank you for pointing the issue out. To avoid any further confusion, we have completely removed the term clustering, and now use "merging" throughout the manuscript..

Coverage: Since "coverage" often refers to sequencing depth, a critical quality metric in DNA sequencing, it might be clearer to use "copy-number coverage" or a similar term. For example, the sentence "Next, samples with low coverage were removed using the…" could be ambiguous if read without context.

Response: We agree with the possibility of confusion. To prevent it, we have everywhere replaced the term "coverage" with a form of the suggested: "CN-coverage".

At the end of the subsection "Explainability and the Effect of SOX2 Gene," the phrase "which exhibits significant local amplification in LUSC" should be revised to "which exhibits significant

focal amplification in LUSC." The correct terminology is "focal" rather than "local," as established in Beroukhim et al. (2010).

Response: Thank you for the correction. Due to the changes to this section, the original sentence has been removed altogether.
