# Supplementary material for: CNSistent integration and feature extraction from somatic copy number profiles
Source: Gigascience. 2025 Sep 13;14:giaf104. doi: 10.1093/gigascience/giaf104 (PMC12448185; doi:10.1093/gigascience/giaf104)
Supplement: giaf104_GIGA-D-24-00595_Revision_2 [file giaf104_giga-d-24-00595_revision_2.pdf]

GigaScience

CNSistent integration and feature extraction from somatic copy number profiles

--Manuscript Draft--

**Manuscript Number:**	GIGA-D-24-00595R2
**Full Title:**	CNSistent integration and feature extraction from somatic copy number profiles
**Article Type:**	Research
**Funding Information:**	German Ministry for Education and Research (01IS18025A, 01IS18037A) — Prof. Dr. Roland F Schwarz<br>Cancer Research Center Cologne Essen (CCCE) — Dr. Adam Streck<br>Bruno und Helene Jöster Foundation (CLONETRAC) — Prof. Dr. Roland F Schwarz
**Abstract:**	The vast majority of cancers exhibit Somatic Copy Number Alterations (SCNAs)—gains and losses of variable regions of DNA. SCNAs play a key role in cancer adaptation through modulation of gene expression, deletion of tumour suppressor genes or amplification of oncogenes. Systematic analysis of SCNAs is now a routine task in both the clinic and research, and can help identify novel cancer genes, improve our understanding of cancer gene regulation and enable us to accurately reconstruct cancer phylogenies. To conduct such analyses however SCNA profiles have to be integrated between samples, patients, and cohorts, an often non-trivial task, for which dedicated toolkits are lacking.<br>To fill this gap, we developed CNSistent, a Python package for imputation, filtering, consistent segmentation, feature extraction, and visualization of cancer copy number profiles from heterogeneous datasets. We demonstrate the utility of CNSistent by applying it to the publicly available TCGA, PCAWG, and TRACERx cohorts. We compare the effect of sample preprocessing and of different segmentation and aggregation strategies on cancer type and subtype classification tasks using various classification models. We also evaluate how well a classifier trained on one cohort generalizes to another. Lastly, we introduce two segment-based peak and outlier scores to investigate relationships between segments, between samples, and between cancer types. Using these scores, we investigate non-small cell lung cancer samples, highlighting that SOX2 amplification is the dominant copy number alteration in lung squamous cell carcinoma and the main distinction to lung adenocarcinoma.
**Corresponding Author:**	Adam Streck, Dr. rer. nat.<br>University Hospital Cologne: Universitatsklinikum Koln<br>Cologne, GERMANY
**Corresponding Author Secondary Information:**	
**Corresponding Author's Institution:**	University Hospital Cologne: Universitatsklinikum Koln
**Corresponding Author's Secondary Institution:**	
**First Author:**	Adam Streck
**First Author Secondary Information:**	
**Order of Authors:**	Adam Streck<br>Roland F Schwarz
**Order of Authors Secondary Information:**	
**Response to Reviewers:**	Below please find the text-only response to reviews. For the version with included images and text formatting, please see the document "CNSistent_Reponse_To_Reviews_2.pdf".<br><br>Reviewer #1:

Powered by Editorial Manager® and ProduXion Manager® from Aries Systems Corporation

The only comment I'd make is with point 6. Metadata effects: Age influences the copy number alterations. The authors don't consider age or any other metadata and their implication in the classification task.

I agree with the authors that controlling for age is non-trivial. However, explicitly including it as a covariate could introduce collider bias (i.e if age is correlated with a specific cancer type it will be highly predictive even if it's effect on copy number profiles are trivial). The commentary around predictive accuracy is also not that useful as (for e.g) age might be highly predictive for one of the classes but not the others- accuracy here will not reflect this.

In some ways if prediction is the end goal it doesn't really matter if CNA profiles have age signatures that improve this prediction. Of course, if interpretation is the end goal, then understanding age specific factors is important. In this case, probably what would be required is some sort of model that predicts copy number at each genomic window- with a cancer type specific age covariate, a global age covariate and a cancer type covariate. Probably the age covariates would be shared across all genomic windows (or maybe could be chromosome specific but likely window specific would be too fine grained). This could be interesting future work to explore but is not necessary for this work.

We would like to thank Reviewer #2 for taking on the responsibility of also taking care of the responses to the comments from Reviewer #1. To observe if there is indeed not a trivial bias introduced through age as suggested by the reviewer, we have tested 1-vs-all binary linear classifiers on balanced datasets (also now included with the code) for the top 6 cancer types we use in classification, i.e. for each class we selected all the samples in the class, and a random subsample of the remaining classes of the same size and attempted to distinguish between the two using age as the only classification variable. The results were the following:

KIRC: Accuracy = 0.5278
OV: Accuracy = 0.5934
BRCA: Accuracy = 0.5972
LUAD: Accuracy = 0.5930
LUSC: Accuracy = 0.6154
PRAD: Accuracy = 0.5764

These data suggest that age is not highly predictive of any specific cancer type in our cohort. Looking at the individual distributions we can see that the only apparent distinction is between lung cancers and the rest, as seen on the reviewer figure, which we reproduced below:

[Reviewer Figure 1]

We therefore do not believe age to be a direct confounder in our case.

We also investigated the relationship between age and copy number alterations in more detail, using the number of breakpoints in a sample as a proxy for the number of structural variants. If there was a strong effect of age on the copy number landscape, we should see a correlation between the number of breakpoints and age, however we did not find a strong correlation in the 12009 samples tested. We have added the result of this analysis as a new Supp. Figure 7, reproduced here:

[Supp. Figure 7]

To reflect these thoughts we have inserted the following paragraph in the Results section:

"We only considered segments on autosomes as the sex of the patient acts as a confounder, in particular for BRCA, OV, and PRAD. To evaluate any possible confounding effect of age we compared the number of breakpoints to the age of the patients across the cohort (n=12009), and found it to only have a minor effect (r=0.11, Supp. Fig. 7). Similarly, age alone was a poor predictor of cancer type, achieving a mean test accuracy on a class-balanced one-versus-all linear classifier of 59.03% and

Powered by Editorial Manager® and ProduXion Manager® from Aries Systems Corporation

a validation accuracy for the multi-class linear classifier of 31.78%."

The above is of course an analysis of a linear relationship between the age and the alterations. Non-linear relationships are likely to exist, although it is not clear to us to what extent, however we felt that a deeper analysis of the topic goes beyond the scope of the article.

Reviewer #2: The authors have done a great job of addressing reviewer comments, adding further analysis and making the manuscript clearer.

(Very) Minor comments:

"To conduct such analyses however SCNA profiles have to be integrated between samples, patients, and cohorts, an often non-trivial task, for which dedicated toolkits are lacking." - This abstract sentence is clunky/poorly punctuated.

We have changed the word order to the following: However, to conduct such analyses SCNA profiles have to be integrated between samples, patients, and cohorts—often a non-trivial task, for which dedicated toolkits are lacking.

"The following was not declared in the manuscript and therefore has been set to default PyTorch values" - maybe change this to directly reference the manuscript in question (i.e Attique et al?)"

As suggested, we have changed the sentence to: The following was not declared in Attique et al.18 and therefore has been set to default PyTorch values.

"We have introduced CNSistent, a new Python-based library for processing and exploratory data analysis of SCNA profiles. Using the PCAWG, TCGA, and TRACERx datasets." Punctuation

We thank the reviewer for spotting the issue. We have corrected the text to the following: We have introduced CNSistent, a new Python-based library for processing and exploratory data analysis of SCNA profiles and applied it to the PCAWG, TCGA, and TRACERx datasets.

Discussion, suggest for:
Unfortunately, the comparison of our models to the one from Attique et al18 was partially limited by the fact that the authors did neither provide all of the model parameters, nor the model source code, and that the source dataset was no longer available at the time of writing this article. Validation on a hold-out set or cross-validation has not been conducted by the authors, we therefore only performed our comparisons on the test accuracies. All models and the input dataset used in this study are available online (see Data and code availability section).

Something like:
"We adapted the CNN originally introduced in Attique et al, and showed superior performance, with the caveat that the original model parameters, source code and source dataset were not available at the time of writing this article. All models and the input dataset used in this study are available online (see Data and code availability section)." And change the location of "Validation on a hold-out set or cross-validation has not been conducted by the authors, we therefore only performed our comparisons on the test accuracies." to be in the results section, where you compare to their results directly.

We agree with the reviewer and we have updated the suggested sentence with minor modifications. The updated Discussion now contains: "We adapted the CNN and DNN3 models originally introduced in Attique et al18 and showed superior performance. The comparison is however limited, since the original model parameters, source code and source dataset were not available at the time of writing this article. All models and the input dataset used in this study are available online (see Data and code availability section)."

The second part we placed in the Results section as follows: "Validation on a hold-out

	set or cross-validation has not been conducted by the authors, we therefore only performed our comparisons on the test accuracies. The best test accuracy (maximum of 5 folds) was 92.42% on 1 Mb segments with the CNN+ mode, slightly above the best test accuracy of Attique et al. (92%).”<br><br>Typo/grammar:<br>"which likewise all additionally we used a normalized Manhattan Distances score to detect outlier samples"<br><br>We thank the reviewer for noticing the error, we corrected the text to the following: …which are likewise all located on chromosome 3. Additionally, we used the normalized Manhattan Distances score to detect outlier samples…<br><br><br>Reviewer #3: The revised is improved and much more readable. No further comments.
**Additional Information:**	
**Question**	**Response**
Are you submitting this manuscript to a special series or article collection?	No
**Experimental design and statistics**<br><br><br>Full details of the experimental design and statistical methods used should be given in the Methods section, as detailed in our Minimum Standards Reporting Checklist. Information essential to interpreting the data presented should be made available in the figure legends.<br><br><br>Have you included all the information requested in your manuscript?	Yes
**Resources**<br><br><br>A description of all resources used, including antibodies, cell lines, animals and software tools, with enough information to allow them to be uniquely identified, should be included in the Methods section. Authors are strongly encouraged to cite Research Resource Identifiers (RRIDs) for antibodies, model organisms and tools, where possible.<br><br><br>Have you included the information requested as detailed in our Minimum Standards Reporting Checklist?	Yes

**Availability of data and materials**<br><br>All datasets and code on which the conclusions of the paper rely must be either included in your submission or deposited in publicly available repositories (where available and ethically appropriate), referencing such data using a unique identifier in the references and in the "Availability of Data and Materials" section of your manuscript.<br><br>Have you have met the above requirement as detailed in our Minimum Standards Reporting Checklist?	Yes
GigaScience has policies and guidelines in place for the use of generative AI-writing tools such as ChatGPT. If you have used such writing tools to assist with writing the manuscript this must be declared and cited in the text. Authors should not list AI-writing tools and other AI-assisted technologies as an author or co-author and should acknowledge that they are fully responsible for text generated or refined by AI-writing tools.<p><br><br>A summary of use (particularly in the introduction or among methods) needs to be included at the end of the paper, and the outputs should also be included as a supplementary file hosted in GigaDB or other open repositories. Please read our guidelines for more information. <p><br><br>By submitting to GigaScience, you are aware of the journal's AI-writing tools policy, and if you have declared use of such tools below, you have acknowledged this where appropriate in your manuscript and have made a summary of use and outputs available. <p><br><b>AI-assisted writing tools have been used in the preparation of this manuscript?	No

Manuscript
Click here to access/download;Manuscript;Manuscript CNSistent.pdf

CNSistent integration and feature extraction from somatic copy number profiles

Adam Streck[1,3], Roland F. Schwarz[1,2,3]

1) Institute for Computational Cancer Biology (ICCB), Center for Integrated Oncology (CIO), Cancer Research Center Cologne Essen (CCCE), Faculty of Medicine and University Hospital Cologne, University of Cologne, Germany

2) Berlin Institute for the Foundations of Learning and Data, Berlin, Germany

3) Berlin Institute for Medical Systems Biology, Max Delbrück Center for Molecular Medicine in the Helmholtz Association, Berlin, Germany

Contact: roland.schwarz@iccb-cologne.org

Abstract

The vast majority of cancers exhibit Somatic Copy Number Alterations (SCNAs)—gains and losses of variable regions of DNA. SCNAs play a key role in cancer adaptation through modulation of gene expression, deletion of tumour suppressor genes or amplification of oncogenes. Systematic analysis of SCNAs is now a routine task in both the clinic and research, and can help identify novel cancer genes, improve our understanding of cancer gene regulation and enable us to accurately reconstruct cancer phylogenies. However, to conduct such analyses SCNA profiles have to be integrated between samples, patients, and cohorts—often a non-trivial task, for which dedicated toolkits are lacking.

To fill this gap, we developed CNSistent, a Python package for imputation, filtering, consistent segmentation, feature extraction, and visualization of cancer copy number profiles from heterogeneous datasets. We demonstrate the utility of CNSistent by applying it to the publicly available TCGA, PCAWG, and TRACERx cohorts. We compare the effect of sample preprocessing and of different segmentation and aggregation strategies on cancer type and subtype classification tasks using various classification models. We also evaluate how well a classifier trained on one cohort generalizes to another. Lastly, we introduce two segment-based peak and outlier scores to investigate relationships between segments, between samples, and between cancer types. Using these scores, we investigate non-small cell lung cancer samples, highlighting that SOX2 amplification is the dominant copy number alteration in lung squamous cell carcinoma and the main distinction to lung adenocarcinoma.

Keywords

cancer, data processing, SCNA, deep learning, cancer classification

Introduction

Somatic copy number alterations (SCNAs)—gains and losses of long regions of DNA—are found across almost all cancer types and are one of the key defining features separating cancer cells from normal cells[1]. It has been demonstrated that quantifying SCNAs has predictive value in the clinic for both progression free and overall survival [2,3] and that they

can serve as sensitive biomarkers for cancer classification and subtyping[4]. We and others have shown that many cancers demonstrate ongoing chromosomal instability (CIN) and continuously accumulate SCNAs throughout their evolution[5], and that SCNAs are excellent markers for inferring cancer evolution[6,7]. Recently, copy number signatures have linked SCNAs to their underlying molecular mechanisms, further strengthening their prognostic value[8,9].

SCNA profiles are commonly derived from a variety of experimental techniques, including SNP arrays, whole-exome and whole-genome sequencing[10], and recently also increasingly from single-cell sequencing[11]. One major advantage of SCNAs over other genomic data types including somatic single nucleotide variants (SNVs) is ease of handling. Due to their aggregate nature, SCNA profiles of individual patients can be published without concern for privacy and the resulting access restrictions, leading to a growing set of publicly available and easily accessible samples from large cohorts such as TCGA, ICGC[12], and the TRACERx[13] lung and renal cancer cohorts.

Unfortunately, copy number profiles, typically defined as lists of segments with given start and end positions and copy number states, are not directly comparable across samples, patients or cohorts. For example, for phylogenetic reconstructions within a patient, profiles have to undergo minimum consistent segmentation where breakpoints are shared between samples to enable evolutionary comparisons [6,7]. For machine learning classifiers, profiles are often aggregated in fixed-width bins or on the gene level. Additionally, different experimental techniques and different copy number calling algorithms can lead to specific biases, missing data, and varying resolutions, further complicating the matter.

To foster reproducible research and avoid reimplementation of common tasks, a tool that enables integration and joint segmentation and thereby caters to the specific demands of copy number profiles would be desirable. To our knowledge, the only available tool that does not require access to the raw sequencing data is the web-based application CNApp[14], which due to its web-based nature is not easily integratable into data science workflows and was not available at its hosted site at the time of this writing.

To fill this gap, we here present CNSistent, a Python package for preprocessing, consistent segmentation, integration, statistical analysis and visualization of SCNA profiles coming from heterogeneous data sources. We demonstrate the utility of CNSistent by integrating available copy number profiles from the TCGA, PCAWG and TRACERx cohorts. We evaluate various segmentation strategies, comparing the performance of deep learning-based multiclass cancer classification tasks and on the classification of non-small cell lung carcinomas (NSCLC) and demonstrate the use of CNSistent for enabling phylogenetic inference from copy number profiles using the minimum consistent segmentation algorithm.

Methods

CNSistent processes SCNA profiles using a multi-step approach. Input data takes the form of copy number segment tables with either allele-specific or total copy numbers (Fig. 1A). The processing is identical for both allele-specific and total copy numbers, however some of the statistics are limited in the case of copy numbers, as detailed below. Optionally,

exclusion regions can be provided to the pipeline to remove locations in the genome where we expect lower quality of information. CNSistent first calculates the proportions of missing genome, which we here refer to as *CN-coverage*, and then utilises imputation strategies to fill in missing data (Fig. 1B). CNSistent then calculates information about breakpoints in each sample. Using the imputed data here has the advantage that spurious breaks are not created between non-consecutive regions purely by missing data. Once the data are imputed we remove the exclusion regions and calculate statistics relating to aberrant copy number values. In the final step, CNSistent offers various strategies for creating a consistent segmentation across samples (Fig. 1D), which are subsequently aggregated to create a final set of complete SCNA profiles with shared segment boundaries for all samples. The pipeline is fully modular and the steps can be skipped or executed in different order. Note that we use the term *segmentation* to refer to a consistent segmentation between samples, i.e. a set of positions inside each chromosome that split the chromosome into segments.

For its calculations, CNSistent can work with any reference genome; hg19 and hg38 reference assemblies are provided as a default. If the sex of the donors is not provided, CNSistent will determine the sex for each sample based on the presence of the Y chromosome.

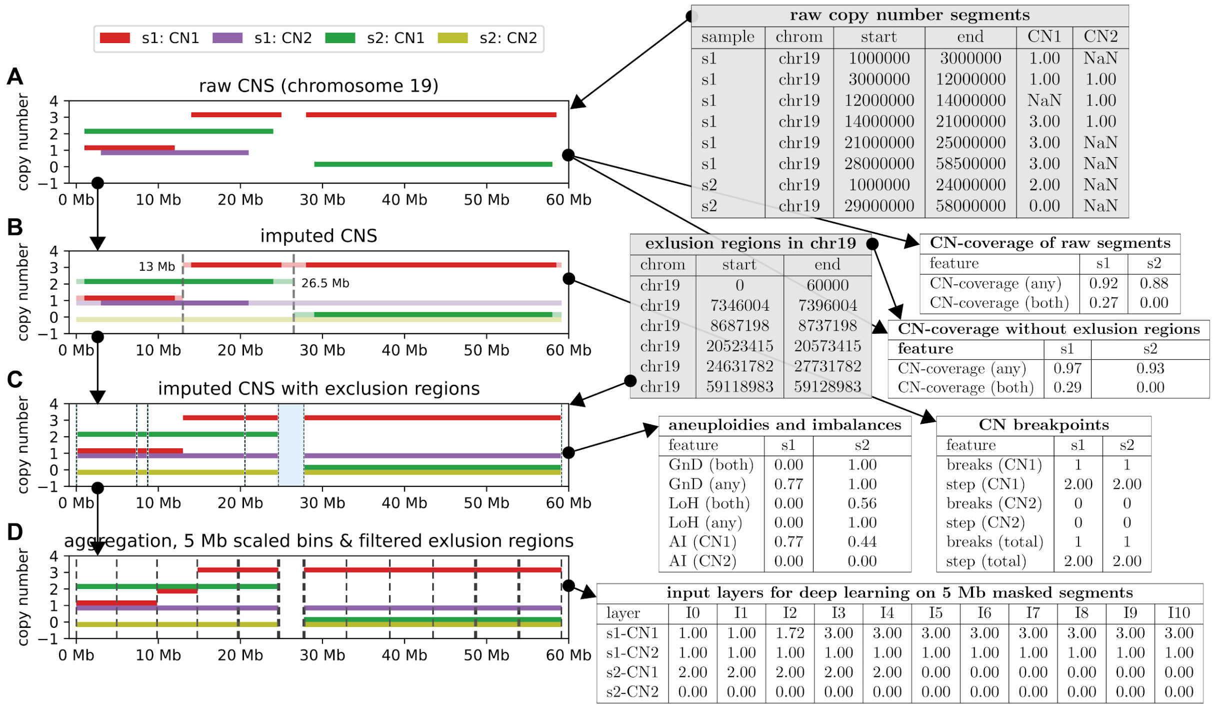

raw copy number segments					
sample	chrom	start	end	CN1	CN2
s1	chr19	1000000	3000000	1.00	NaN
s1	chr19	3000000	12000000	1.00	1.00
s1	chr19	12000000	14000000	NaN	1.00
s1	chr19	14000000	21000000	3.00	1.00
s1	chr19	21000000	25000000	3.00	NaN
s1	chr19	28000000	58500000	3.00	NaN
s2	chr19	1000000	24000000	2.00	NaN
s2	chr19	29000000	58000000	0.00	NaN

exlusion regions in chr19		
chrom	start	end
chr19	0	60000
chr19	7346004	7396004
chr19	8687198	8737198
chr19	20523415	20573415
chr19	24631782	27731782
chr19	59118983	59128983

CN-coverage of raw segments		
feature	s1	s2
CN-coverage (any)	0.92	0.88
CN-coverage (both)	0.27	0.00

CN-coverage without exlusion regions		
feature	s1	s2
CN-coverage (any)	0.97	0.93
CN-coverage (both)	0.29	0.00

aneuploidies and imbalances		
feature	s1	s2
GnD (both)	0.00	1.00
GnD (any)	0.77	1.00
LoH (both)	0.00	0.56
LoH (any)	0.00	1.00
AI (CN1)	0.77	0.44
AI (CN2)	0.00	0.00

CN breakpoints		
feature	s1	s2
breaks (CN1)	1	1
step (CN1)	2.00	2.00
breaks (CN2)	0	0
step (CN2)	0	0
breaks (total)	1	1
step (total)	2.00	2.00

input layers for deep learning on 5 Mb masked segments											
layer	I0	I1	I2	I3	I4	I5	I6	I7	I8	I9	I10
s1-CN1	1.00	1.00	1.72	3.00	3.00	3.00	3.00	3.00	3.00	3.00	3.00
s1-CN2	1.00	1.00	1.00	1.00	1.00	1.00	1.00	1.00	1.00	1.00	1.00
s2-CN1	2.00	2.00	2.00	2.00	2.00	0.00	0.00	0.00	0.00	0.00	0.00
s2-CN2	0.00	0.00	0.00	0.00	0.00	0.00	0.00	0.00	0.00	0.00	0.00

Figure 1: Illustrative example of processing of two SCNA profiles (s1, s2) for two alleles (CN1, CN2), on human chromosome 19. ***A)*** *The input data (gray tables) consist of non-contiguous major and minor copy number segments for each sample. From this the proportion of the genome that is missing is calculated for each sample. For comparison, the CN-coverage is calculated both with and without considering the gap regions. Note that as there are no minor CNs for s2, the homozygous CN-coverage is 0.* ***B)*** *During imputation two new breakpoints are introduced at 13 Mb and 26.5 Mb, while the breakpoints on the boundaries of missing segments are no longer present. From the imputed data CNSistent calculates the CN breakpoint-related statistical features.* ***C)*** *Ploidy and allelic imbalance-related statistical features that are derived from the imputed data and removal of the gap regions.* ***D)*** *Small regions are not used in region exclusion, retaining only the gap between 20 and 30 Mb, which splits the chromosome into two arms, which are then further split into ~5 Mb bins. The same-size strategy is used, meaning that the bins in the left segment are slightly smaller (4.9 Mb), while the ones on the right are slightly bigger (5.27 Mb). Each profile is then converted into a vector of CN values for downstream analysis. Note that as there was a breakpoint at 13 Mb, the resulting value is a weighted mean of the previous values, i.e. 1.72.*

Imputation of missing values

SCNA profiles from different cohorts often vary in the extent to which they span the genome. This can be due to a variety of reasons including different underlying technologies (WES vs WGS sequencing), different segmentation strategies, or different exclusion of regions surrounding the centromeres and telomeres. To retain as much information as possible, CNSistent offers an imputation step capable of filling the gaps in SCNA profiles using an *extension* method (Fig. 1C).

The *extension* imputation method executes the following five steps: (i) Segments are pruned such that they are fully contained within the coordinates and named chromosomes of the reference genome. (ii) CNSistent extends the first and last segment of each chromosome to the chromosome boundaries. (iii) Each gap between two segments is split into two halves (rounded down), and each half is then assigned the CN of its neighboring segment. (iv) If any chromosomes are fully missing from the sample, they are set to 0. (v) The neighboring segments that have the same CN are merged.

Alternatively, two additional imputation options are available: *diploid* and *null*. The diploid method changes the steps (ii-iv) in such a way that all newly created segments are set to diploid, e.g. if a sample is male and major/minor CN columns are used, CNSistent will create a segment on the whole chromosome Y with major and minor CN of 1 and 0 respectively. The null option will analogously fill all the newly created segments with 0.

Feature extraction

CNSistent can calculate a set of statistical features. As CNSistent is sex chromosome aware, the length of the linear genome depends on the sex of the sample. Each feature is therefore calculated three times: for autosomes, for sex chromosomes, and for the whole genome:

CN-coverage: Calculates the proportion of the whole genome where any CN value is assigned (as opposed to missing values). In case of allele-specific CNs, both mono-allelic CN-coverage (either allele has a CN value assigned) and bi-allelic CN-coverage (both alleles must have a CN value assigned) are calculated.

Genome not Diploid (GnD): Defines the proportion of the genome where an allele does not have the CN a diploid cell of the same sex would have. In case of having only total CN this is a lower bound approximation.

Loss of Heterozygosity (LoH): Calculates the proportion of segments with CN=0 on either allele (hemizygous) or on both alleles (nullizygous). The segment is only considered LoH if and only if its CN value is 0 and its normal value is not zero (e.g. chromosome Y for female samples).

Allelic imbalance (AI): The proportion where one allele has a strictly higher CN than the other.

Breakpoints: The number of breakpoints per chromosome for each allele. If two column format is used, a total number of breakpoints is also calculated to account for cases where

both alleles have a breakpoint in the same location (meaning that the total number of breakpoints is less than the sum of the alleles).

Breakpoint Step: The mean difference between the CNs of consecutive segments. Note that it is preferable to impute the segments first to avoid inducing spurious gaps.

Consistent segmentation

One major goal of CNSistent is to obtain segments that are consistent between sample sets and from which then features can be derived in a unified manner. This requires the same set of breakpoints to be present in every sample. Segmentation consists of the following 4 steps: (i) define regions of interest (e.g. whole chromosomes, coding genes, etc.), (ii) remove exclusion regions (e.g. telomeric or centromeric regions), (iii) share existing breakpoints between samples and merge them based on a distance threshold, and/or (iv) subdivide the segments into fixed-width bins. Each of the four steps is optional.

The segments for step (i) can be provided as a BED file, or one of five predefined options can be used: whole chromosomes (default option), chromosome arms, cytobands, COSMIC consensus cancer gene set[15], or the Ensembl coding genes set[16]. From these segments, exclusion regions can be optionally removed (Fig. 1D). As a default option, the regions of low mappability as defined by the UCSC[17] genome browser are provided. During the exclusion process, if the regions are small or close to each other, fragmentation can occur. This can be avoided by segment filtering—the user specifies a filter of size f, where any exclusion region smaller than f is removed, and likewise if after the exclusion regions are removed from segmentation, any newly created segments smaller than f are also removed.

The breakpoints are then merged using a greedy algorithm on a predefined region (usually a whole chromosome). Starting from the leftmost breakpoint, all breakpoints within the merge distance m are accumulated and a new breakpoint is created at their average. This is then repeated from the leftmost not yet merged breakpoint, until the end of the region is reached. A detailed example is shown in Supp. Fig. 1.

Lastly, the resulting segments can be subdivided into smaller bins based on user defined split size s (Fig. 1E). Three subdivision strategies are provided: (a) From the start of the segment, breakpoints are inserted every s bases. Here, the last bin is likely to be of a different size. If it is smaller than $s/2$, it is merged with the previous segment. (b) Is similar to (a) where instead of creating the padding only at the end, the padding is split in half and added to both ends. Likewise, if the first and last bins are smaller than $s/2$, they are merged with their neighboring segments. (c) The bins are scaled so that they are all the same length, slightly different from s. Consider a segment that has c bins, including the padding–If the padding is smaller or equal to $s/2$, split the segment into $c - 1$ equally sized bins, otherwise into c bins.

Aggregation of copy numbers

After joint segmentation, the copy numbers from the original segments are aggregated to create CNs for the new segments. First the old segments are split at the breakpoints given by the new segmentation. Second, the resulting refined segments are aggregated between the breakpoints given by the segmentation, using one of four possible aggregation

strategies: The *Min* and *Max* strategies will assign the minimum or maximum CN to the whole segment–the *Min* strategy is particularly relevant when considering genes, since incomplete segments are unlikely to yield functional gene copies. The *Mean* strategy will take a mean of CNs across bins weighted by their lengths, preserving the overall CN per sample. Lastly, merging can be skipped altogether, which can be used if we want to select only a subsection of each profile, e.g. only q-arms.

Sample filtering

The features obtained in the feature extraction step can be used to filter undesirable samples. For base quality metrics, like CN-coverage, a simple z-score outlier detection method is provided, meaning that for a feature f over a set of samples S, $z = \frac{f(S) - \mu(f(S))}{\sigma(f(S))}$ is calculated and samples greater than 3 standard deviations from the mean ($|z| \geq 3$) are removed. The value 3 is a typical threshold for the method, but it can be adjusted by the user.

In certain cases, a qualitative separation of data is preferable, e.g. to remove samples with negligible SCNA activity. CNSistent offers an automated solution for finding such thresholds using a knee-detection algorithm. A knee-point is a point of the plot where the maximum angle between the line to the first point and the last point of the plot. To find the knee-points for a feature f in a set of samples S, a tuple of monotonically increasing feature values $T = (min(f(S), ..., max(f(S)), \forall i \in 1, |T| - 1: t_i \leq t_{i+1}$ and a cumulative distribution of values smaller than each threshold $Y = (|f(S) \leq t|)_{\{t \in T\}}$ is created. Second, T is normalized such that $\forall t \in T$: $t' = \frac{t - t_1}{t_n - t_1}$, and analogously for Y'. The knee-point is then the $t_i, 1 \leq i \leq n$ with the maximum angle between the vector from origin to the normalized threshold, (t_i', y_i'), and the vector from the threshold to the endpoint, $(1 - t_i', 1 - y_i')$. If the angle is negative (clockwise rotation), we call it a *knee*, otherwise (counter-clockwise rotation), we call it an *elbow*. A visualization of the method is provided in Supp. Fig. 2.

Outlier detection

CNSistent can detect outlier samples based on the normalized Manhattan Distance (NMD) between pairs of samples. To calculate NMD we normalize each sample by dividing the value of each bin by its sum. This normalization allows us to ignore the effects of whole genome doubling, since the normalized values are the same before and after WGD. Formally, having two aggregated samples $S = (s_1, \cdots, s_n)$, $R = (r_1, \cdots, r_n)$, the $NMD(S, R) = \sum_{j=1}^{n} |\frac{s_i}{\Sigma(S)} - \frac{r_i}{\Sigma(R)}|$. To compare a sample S to a cluster of samples $C = (S_1, \cdots, S_m)$, we calculate the outlier score $OS(S, C) = \frac{\sum_{j \in 1}^{m} NMD(S_i, S_j)}{|C|}$. To compare between two cancer types $C1$, $C2$ and a sample $S \in C1$ we extend the outlier score as $OS(S, C1, C2) = OS(S, C2) - OS(S, C1)$.

Peak detection

To find regions of interest in the samples, CNSistent provides the peak score (PS), which shows how much each bin differs from its neighbours. Have an aggregated sample $S = (s_1, \cdots, s_n)$ we set the boundary values $s_0 = s_1$, $s_{n+1} = s_n$ and calculate $\forall i \in (1, \cdots, n)$: $PS(S, i) = (s_i - s_{i-1}) - (s_{i+1} - s_i)$. This score will be positive for segments higher than their neighbours, negative for those lower and close to zero for segments with monotonous behaviour. We therefore use the PS to detect the highest and lowest values, which show the locations of most abrupt change in CN accumulation. Note that for meaningful calculation this requires that the segments are connected to each other and about the same size.

Identifying discriminatory features

To identify features that most differ between groups of samples, we use the Mann-Whitney U-Test using the `mannwhitneyu` function in scipy v1.15.0. All p-values are corrected for multiple tests using the `multipletests` function with Benjamin-Hochberg correction in statsmodels v0.14.0.

Machine learning

To evaluate how different filtering and segmentation strategies affect the downstream analysis, we used two cancer type classification tasks: classifying between 6 types with the most samples, as introduced in *Attique et al.*[18] and NSCLC classification, as introduced in *Qiu et al.*[19]. In this task, each binned sample as illustrated in Fig. 1 represents one feature vector. The output probability that a sample belongs to each cancer class under consideration. We then compare 4 different classification methods: Random Forest (RF), Elastic Net (ENet), Deep Neural Network (DNN), and Convolutional Neural Network (CNN).

For each of these models we apply 5-fold cross validation, i.e. we split each dataset into 5 groups and always withhold one while training on the other 4. The validation accuracy for each model is then the mean of the test scores of the 5 different splits[20].

As the number of patients per cancer type varies, the classes are imbalanced. To avoid a possible bias due to an overrepresentation of one class, a stratified split is used, meaning that the ratio of the individual cancer classes is preserved across the 5 subsets. Additionally, some samples are obtained through multi-region sampling. While the samples from different regions show different profiles, there is a risk of being able to guess the class based on the similarity to the original profile. This is prevented by sample grouping where each group (in this case patient) can only be part of one subset. The splitting is done using the `StratifiedGroupKFold` object from scikit-learn v1.4.1, which was also used for ENet and RF classifiers.

For ENet we used the `SGDClassifier` with log loss and the `elasticnet` penalty. For RF we used `RandomForestClassifier` with default parameters. For deep learning we used CNN, and DNN3 neural network architectures as described in *Attique et al.*[18], as well as our own extended CNN, which we called CNN+. In summary, the CNN uses two convolutional layers (kernel=5) and RelU activation, followed by maxpool, batch

normalization and dropout after each, followed by a flattening and the output layer with softmax. The DNN3 uses 3 hidden layers with sizes 600, 300, and 150, with batch normalization, dropout and RelU, except for the output layer which uses softmax. The following was not declared in *Attique et al.*[18] and therefore has been set to default PyTorch values: maxpool kernel size of 2, dropout probability of 0.5. Our CNN+ model builds on the CNN, however an additional fully connected layer is added after the flattening layer, with size half in between the flattening and output layer. The CNN+ also uses two separate input channels, one for each allele. The full architecture of CNN+ is given in Supp. Fig. 3.

Optimization was done using the PyTorch library v2.2.1[21], accelerated using CUDA v12.1. Optimization was conducted using the Adam optimizer with a learning rate of 0.001, weight decay of 0.01 and batch size of 64. The error is evaluated using cross-entropy loss. The training was limited to 1000 epochs. The training process was accelerated by an early stopping strategy where the minimum loss is recorded and if past 10 epochs lead to a training loss higher than the existing global minimum, the training stops. To accommodate for the two alleles, we concatenated the major and the minor CNs into a single vector.

Data and code availability

CNSistent package, source data, and plotting notebooks can be downloaded at: https://bitbucket.org/schwarzlab/cnsistent and available on PyPI: https://pypi.org/project/CNSistent/. CNSistent is registered on bio.tools with ID: `cnsistent` and at SciCrunc with RRID: **`SCR_027025`**. The preprocessed input data are available at https://zenodo.org/records/14677713[22]. The data produced by CNSistent are available at https://zenodo.org/records/14547456[23]. The deep learning code and results are available at https://zenodo.org/records/16610677[24]. The deep learning model information is stored in the DOME registry: https://registry.dome-ml.org/review/59bcqzam2c [25].

The data have been obtained from the following sources, accessed in December 2023: PCAWG data obtained from: https://dcc.icgc.org/releases/PCAWG/consensus_cnv[12]; The results published here are in part based upon data generated by the TCGA Research Network: https://www.cancer.gov/tcga. TCGA data obtained from ASCATv3 at: https://github.com/VanLoo-lab/ascat[26]. TRACERx data obtained from: https://zenodo.org/records/7649257[13]. COSMIC cancer set obtained from: https://cancer.sanger.ac.uk/census[27]. Human genome gene set obtained using PyENSEMBL (2023)[16]. Cytoband and Gap data obtained from the UCSC Genome Browser: https://genome.ucsc.edu[28]. For TCGA the ASCAT team called the CNs by ASCATv3 from WGS, the TRACERx team used ASCATv2 from WES and PCAWG consortium published CNs obtained as a consensus of 5 different callers[29] from WGS.

Results

We illustrate the use of CNSistent on a cancer type classification task using 15,072 publicly available SCNA profiles from The Cancer Genome Atlas (TCGA[9], n=10674), the Pan-Cancer Analysis of Whole Genomes (PCAWG[12], n=2778) and the TRACERx cohort of non-small cell lung cancer[13] (n=1620).

Where the TCGA and PCAWG datasets overlap (829 samples), we gave preference to the PCAWG callset. The PCAWG dataset blacklists 195 low-quality samples, which were removed before further processing.The TRACERx dataset consists of two parts, primary tumor samples (n=1428) and primary with metastatic samples (n=694). We used the primary sample set in the 502 samples on which they overlap. This yielded a total set of 14174 SCNA profiles which were subjected to CNSistent for pre-processing and integration. A summary of sample counts is provided in Supp. Table 1.

CNSistent segmentation of 14174 copy number profiles

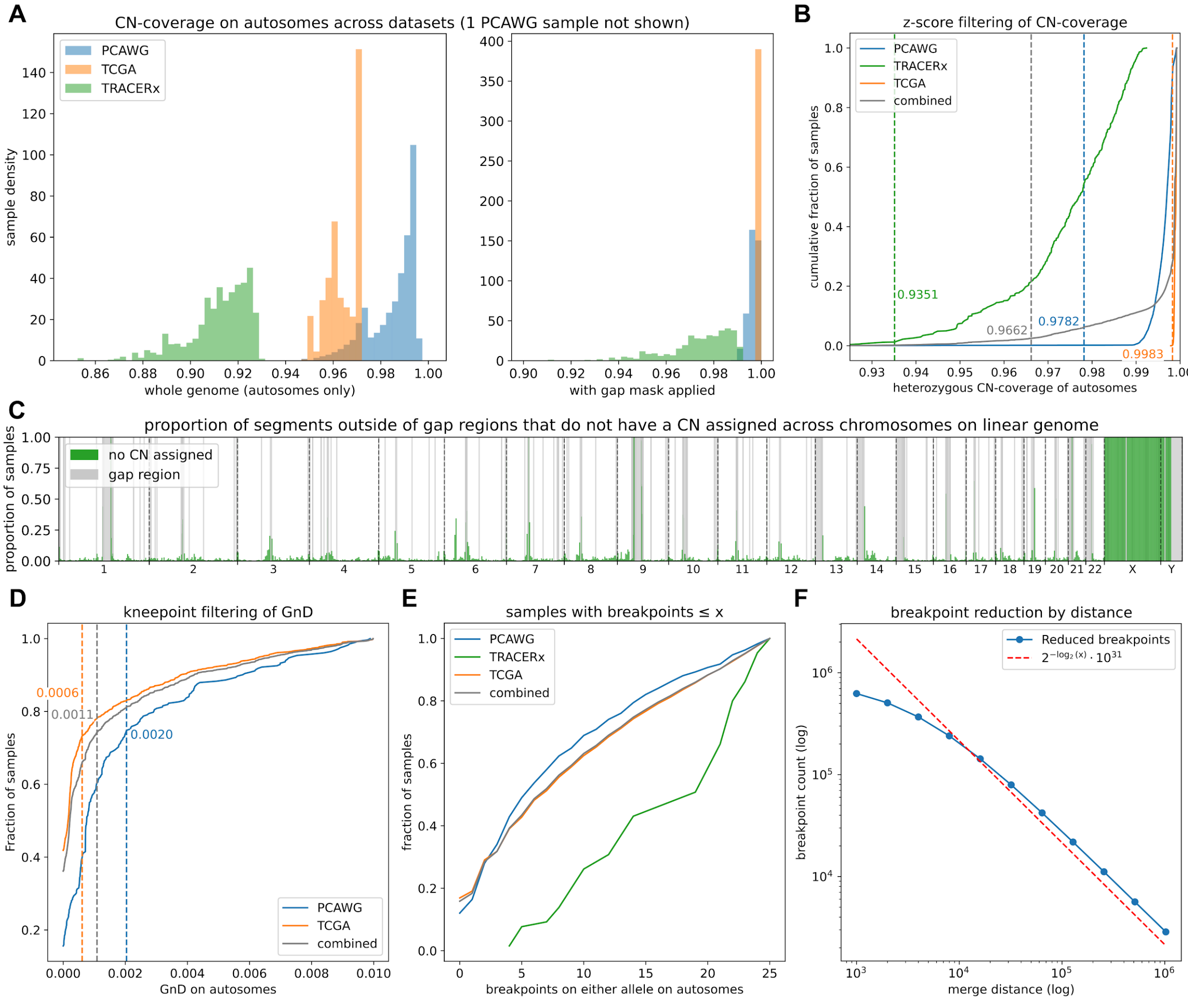

Figure 2: Processing of the PCAWG, TRACERx, and TCGA datasets. ***A)*** *Histograms of heterozygous CN-coverage before and after gap region filtering. Note that the PCAWG and TCGA datasets have almost full CN-coverage after filtering. In contrast, while TRACERx shows a major shift, there are still substantial portions missing.* ***B)*** *Cumulative distribution of samples by heterozygous CN-coverage, with the threshold for filtering given by the z-score. The position of the threshold is much higher for the combined dataset compared to the individual ones.* ***C)*** *Distribution of the missing values in the TRACERx dataset along the linear genome (X and Y*

are not present in the data). Data are mostly missing in regions close to the centromeres and telomeres, in particular for chromosomes 1 and 9. ***D)*** *Cumulative distribution of GnD for a subset of samples below 1%. TRACERx is not shown as none of the samples has hemizygosity below this value. Note the clear slope change around 0.1%, also detected by our kneepoint algorithm.* ***E)*** *Cumulative distribution of breakpoint counts for a subset of samples with less than or equal to 25 breakpoints. The curve is almost linear for all datasets, demonstrating that there's no clear cutoff value in this region.* ***F)*** *The result of breakpoint reduction using 11 log-distributed merge distances between 1 Kb and 1 Mb. Note that the relationship is proportional—doubling the distance leads to halving the number of resulting segments, as shown by the hyperbolic curve.*

We started by imputing any missing data and calculated the sample features (see Supp. Fig. 4 for complete results). Since SCNA profiles for sex chromosomes were not available in the TRACERx cohort, all sex chromosomes were removed from further analysis. Before region exclusion, the SCNA profiles covered on average 98.47%, 96.39%, and 91.18% for PCAWG, TCGA and TRACERx respectively (Fig. 2A). When using the UCSC gap regions for exclusion, the CN-coverage rose to 99.62%, 99.89%, and 97.38%. The gap regions of hg19 on autosomes sum to 19.65 Mb, which is 6.82% of the total genome. For TCGA and PCAWG virtually all the missing segments fell into these gap regions. In TRACERx, there are regions missing also outside these gap regions, however mostly on their boundaries (Fig. 2C). This was likely due to the sequencing method: PCAWG data has been sourced using WGS, whereas TCGA combines multiple data sources.

Next, samples with low CN-coverage were removed using the z-score based outlier detection (Methods). Thresholds were calculated for each of the datasets separately as well as using the combined dataset of all samples (Fig. 2B). For the individual samples there was only a small set of outliers: 3, 16, and 19 for the thresholds of 97.82% for PCAWG, 99.83% for TCGA, and 93.51% for TRACERx respectively. However, when the combined dataset was used, 352 samples were below the detected threshold of 96.62%, stemming from the fact that the CN-coverage distribution of TRACERx significantly differs from the other two. In this case, filtering each set separately leads to significantly lower removal rate. Additionally, one sample in the PCAWG dataset, SP107557, had CN-coverage of only 57.67% and presumably should have been blacklisted in the original dataset.

We also removed samples with few or no copy number alterations. Other authors have used the number of breakpoints[9] as evidence for SCNAs, however we did not observe a clear knee-point in the data (Fig. 2E) and any threshold would therefore be arbitrary. Instead, we used the knee point detection algorithm on the GnD statistic for samples below 1% GnD to determine the following cutoffs (Fig. 2D): 0.06% for TCGA (745 samples removed) and 0.2% for PCAWG (211 samples removed). For TRACERx all samples were retained. The filtering process then leads to the final filtered sample set of 12901 samples (see Supp. Fig. 5 for full sample distribution).

We next evaluated the effects of breakpoint merging. Without any merging, the whole filtered dataset has 826910 unique breakpoints, i.e. one breakpoint per 3.7 Kb on average. We explored different merge distances from 1 Kb to 1 Mb, leading to reductions between 24.39%-99.65% (Fig. 2F), and selected 1 Mb, 500 Kb, and 250 Kb distances, leading to 2797, 5569, and 10797 autosomal segments respectively. To compute all combinations of segmentation strategy and datasets efficiently, we made use of CNSistent's internal parallelization strategy. Runtime decreased in a near-linear fashion with the number of compute cores available (Supp. Fig. 6). All segmentation configurations are listed in Supp. Table 2.

Evaluating segmentation strategies on a cancer classification task

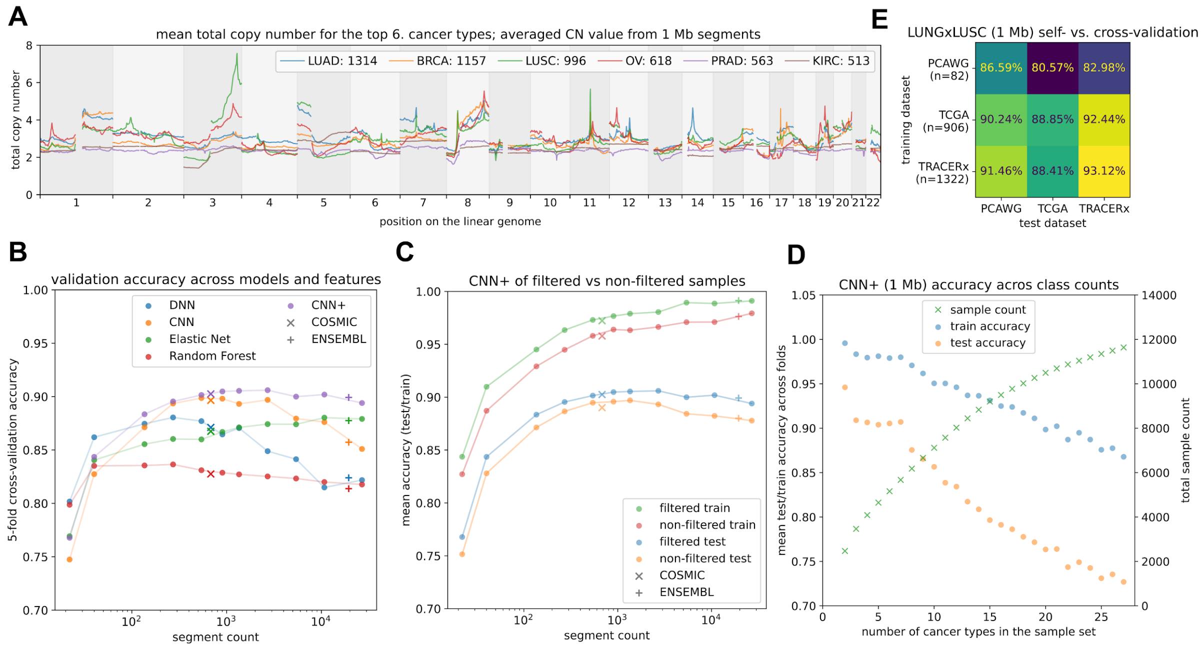

Figure 3: Evaluation of multi-class prediction task. ***A)*** *Averaged SCNA profiles of the 6 cancer classes considered for classification.* ***B)*** *Validation accuracy of the different models tested across increasing granularity of segmentation. DNN and RF quickly reach maximum accuracy and degrade with an increasing number of segments, while the CNN and CNN+ architectures increase until ~1000 segments and ENet even increases monotonously. Results of training on gene-based CNs for each model are displayed using crosses (the number of genes then gives the number of segments).* ***C)*** *Comparison of training on filtered and unfiltered data. We see that both the train and test accuracy improves after filtering. Additionally we see that the training accuracy increases almost monotonously, while the validation degrades after ~1000 segments, likely pointing to overfitting on smaller segments.* ***D)*** *Results of classification across 2-27 classes on 2 Mb segments. We see nearly linear degradation of both training and testing accuracy, however even for 27 classes the accuracy is still over 70%.* ***E)*** *The NSCLC classification task using 2 Mb segments. On the diagonal the models are scored using 5-fold cross-validation on each individual dataset. The remaining values show results of training on one full dataset (row) and validating on another full dataset (column). We can see that in particular training on bigger sets of TCGA and TRACERx yields better results on PCAWG than training on self.*

We next set out to explore the effects of different segmentation strategies on the cancer classification task (see Methods). We processed the data using the following segmentation strategies: (i) fixed-size segments of 20 Mb, 10Mb, 5 Mb, 3 Mb, 2 Mb, 1 Mb, 500 Kb, 250 Kb, 100 Kb size; (ii) Whole chromosomes, chromosome arms; (iii) Gene-level CN values based on the ENSEMBL and COSMIC gene sets; and (iv) breakpoint merging using distance thresholds of 1 Mb, 500 Kb, and 250 Kb. The segment sizes roughly cover the ranges used by other authors for feature discovery[9].

To our knowledge the best result to date on the cancer classification task has been reported on classification of top 6 cancer types in the dataset in Attique *et al.*[18], with up to 92% test accuracy on the best model. Using our combined dataset, the selection of top 6 classes resulted in a set of 5172 samples with the following class labels: lung adenocarcinoma (LUAD, n=1314), breast invasive carcinoma (BRCA, n=1157), lung squamous cell carcinoma (LUSC, n=996), ovarian cancer (OV, n=618), prostate adenocarcinoma (PRAD, n=563), and kidney renal cell carcinoma (KIRC, n=513), with their mean profiles displayed in Fig. 3A.

We only considered segments on autosomes as the sex of the patient acts as a confounder, in particular for BRCA, OV, and PRAD. To evaluate any possible confounding effect of age we compared the number of breakpoints to the age of the patients across the cohort (n=12009), and found it to only have a minor effect (r=0.11, Supp. Fig. 7). Similarly, age alone was a poor predictor of cancer type, achieving a mean test accuracy on a class-balanced one-versus-all linear classifier of 59.03% and a validation accuracy for the multi-class linear classifier of 31.78%.

We compared the DNN3 and CNN architectures of Attique *et al.*, Random Forest, Elastic Net, and our extension of the CNN architecture–CNN+ (Fig. 3B)–across decreasing segment sizes. On whole chromosomes the most performing models reached a validation accuracy of ~80% and considering the arms separately reached almost ~86% on the DNN architecture. Increasing the resolution improves accuracy of the two convolutional models, which peak in the region of around 1000 segments. The best validation (mean of 5 folds) accuracy of 90.60% was achieved with the CNN+ at 1 Mb segments, with full confusion matrix given in Supp. Fig. 8. We used the 1 Mb segments for the subsequent tasks, however sizes from 5 Mb to 250 Kb all had validation accuracy above 90%, and we would therefore consider all of them to be suitable for any further analyses.

The RF and DNN models however peak around 200 segments and increasing the resolution further decreases the validation performance, likely due to overfitting. The only architecture that improved monotonously was ENet, where the penalty regularization seemed to prevent overfitting, however from 20 Mb onwards it always underperforms compared to the CNN+. Comparing the full segmentation with the COSMIC and ENSEMBL gene sets we saw that taking only the CNs for genes performs equivalently to creating a segmentation with a similar number of features. Breakpoint merging performed comparably to bins of the same size, e.g. a 500 Kb merge window showed an accuracy of 90.09%, while 500 Kb segments showed an accuracy of 90.0%. Similarly, considering different aggregation strategies for COSMIC and ENSEMBL has not affected the results significantly: for COSMIC the results were Min: 90.94% Mean: 90.25%, Max: 90.05%. For ENSEMBL: Min: 87.44%, Mean: 89.92%, Max: 89.54%. Validation on a hold-out set or cross-validation has not been conducted by the authors, we therefore only performed our comparisons on the test accuracies. The best test accuracy (maximum of 5 folds) was 92.42% on 1 Mb segments with the CNN+ mode, slightly above the best test accuracy of Attique *et al.* (92%). All the deep learning models trained within 100 seconds on a desktop GPU. Full training times are shown in Supp. Fig. 9. Full training and test results are given in the Supp. Table 3.

To evaluate the results of filtering, we compared the results on filtered (5161 samples) and unfiltered (5257 samples) on the CNN+ model (Fig. 3C). We saw that both training and testing accuracy has been consistently better in the filtered dataset. The average test score improvement was 1.28%. Additionally we were interested in how the CNN+ performs for different numbers of classes. We limited ourselves to classes with at least 100 samples; this yielded 27 classes (Supp. Fig. 5). In Fig. 3D it can be seen that the accuracy is quite high for all the cases and decreases in almost a linear fashion. In the easiest binary classification task we saw 94.6% validation accuracy, while the 27-class task reached 72.69%.

To demonstrate the potential of integration using CNSistent across different datasets we used the NSCLC classification task, training the models on one dataset and validating on another (Fig. 3E). We see that the accuracies of models trained on a different dataset match

or sometimes even outperform models trained and validated on the same dataset, with up to 91.46% accuracy for the TRACERx model applied to PCAWG. We also see that compared to self-training, the models trained on bigger sets (TRACERx, TCGA) outperform self-training on the small PCAWG model. Likewise, training on TRACERx slightly outperforms self-training on TCGA. The 5-fold cross-validation accuracy on the combined dataset was 92.73%, considerably improving on the previous result of 84% in *Qui et al.*[19]. When training the models individually, we obtain only 91.21% mean validation accuracy, showing that combining the datasets leads to a (1.52%) improvement.

Identifying commonly altered regions and outliers

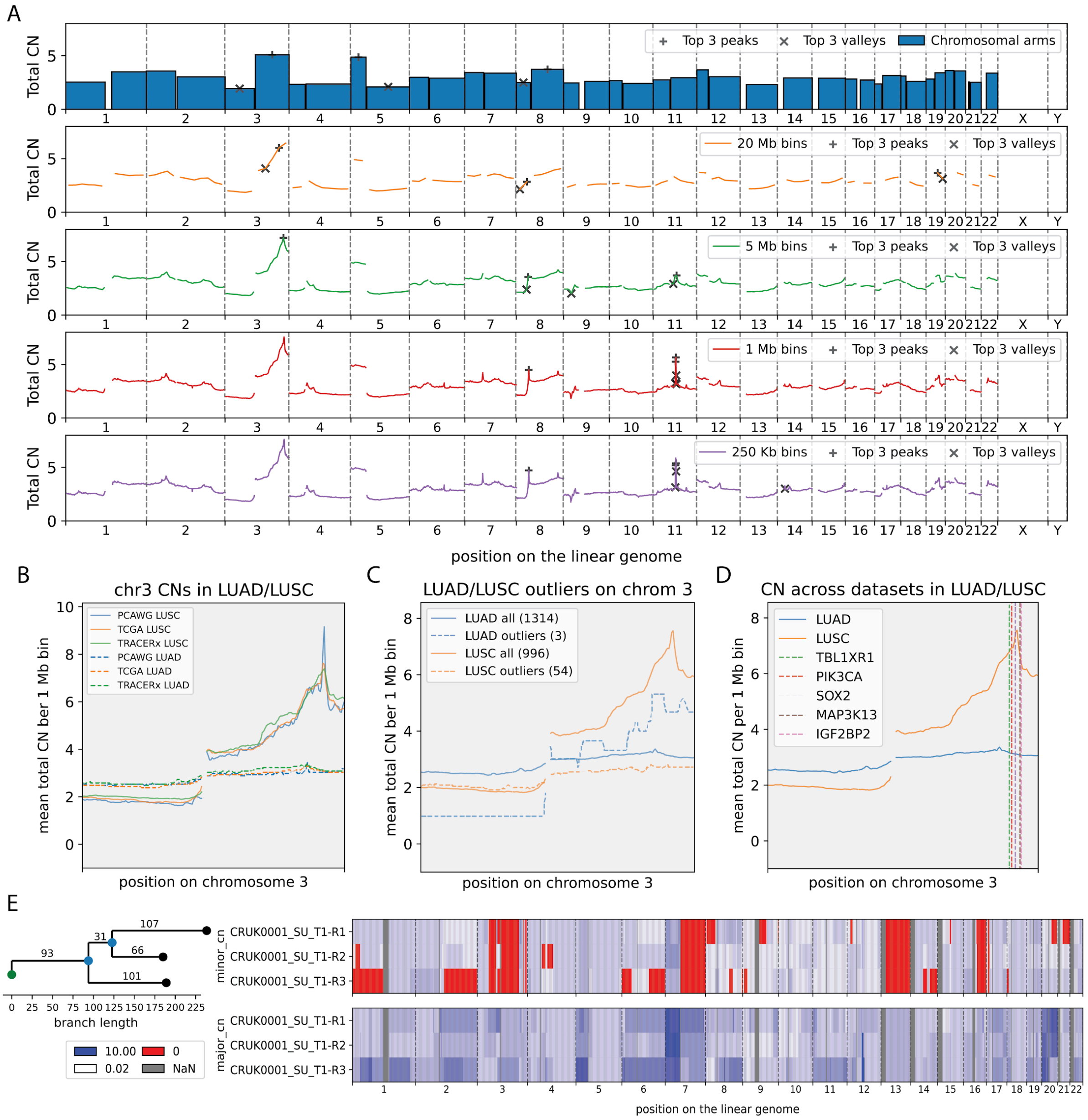

Figure 4: Analyses of LUAD/LUSC CN profiles. Except for the phylogenetic tree, all plots are produced using CNSistent segment plotting functions. ***A)*** *The mean CN profiles of the LUSC samples across different segmentations. For each segmentation, the segments with the 3 highest positive (peaks) and highest negative (valleys) peak scores are shown.* ***B)*** *The 2Mb CN profiles between different cohorts for both LUSC and LUAD, showing strongly correlated patterns of selection between the cohorts of the same cancer type.* ***C)*** *Mean CN profile for LUAD and LUSC with the positions of the top 5 most copy number changed genes.* ***D)*** *Mean CN profiles of both LUAD and LUSC compared to the outlier samples (samples with high NMD its own type and low*

to the other type). ***E)*** *Example of CN heatmap for major and minor CN values in three regions of a single tumor together with inferred phylogeny.*

Lastly we demonstrate our segment-derived metrics on the LUNG-LUSC sample set. First, we investigated how the identification of recurrently altered genomic regions is influenced by the segmentation strategies using our simple peak detection algorithm (Methods). We found that the regions with the highest and lowest peak scores differ between segmentation sizes (Fig. 4A), with chr3 being detected mostly in big segments due to a wide slope on the q-arm, while chr8 is being mostly detected in middle sizes due to focal amplification at the end of the p-arm, and on chr11 there is very narrow peak which becomes most prominent in smaller segmentations.

Next, we investigated all CN profiles for outliers (Methods). The highest NMD between LUAD and LUSC samples is on chromosome 3, where we can see that LUSC has a distinctive, wide peak on chr 3q (Fig. 4B), while LUAD is mostly neutral. This pattern is extremely well correlated across cohorts. We then calculated the outlier score between LUAD and LUSC and used the kneepoint detection to find an outlier threshold (Supp. Fig. 10), finding 3 LUAD samples with LUSC-like pattern (amplification of SOX2) and 54 LUSC samples with neutral LUAD-like pattern (Fig. 4C). We also observed that the majority of these samples (58.75%) came from the TCGA dataset, while the TRACERx dataset had the least outliers (15.79%).

To systematically determine which genes differ significantly in their CNs between LUAD and LUSC, we conducted a Mann-Whitney U-Test with Benjamini-Hochberg correction on the mean CNs of the COSMIC genes. Out of 722 genes, 599 had adjusted p-value below 0.05. The top 5 most copy number altered genes were all on the q-arm of chr3 (Fig. 4D). All these had the adjusted p-value below 10^{-169}, with SOX2[30] being the most significant at $p \approx 10^{-187}$. The SOX2 gene also has the highest mean CN of 7.56.

Lastly, to validate CNSistent segments outside of the tool, we used the 1 Mb segments for phylogeny reconstruction. For this we used MEDICC2[6] and applied it to the first patient in the TRACERx dataset with 3 regions. A CONSistent-produced bar plot of the major and minor CNs and a MEDICC phylogenetic tree are shown in Fig. 4E.

Discussion

We have introduced CNSistent, a new Python-based library for processing and exploratory data analysis of SCNA profiles and applied it to the PCAWG, TCGA, and TRACERx datasets.The main goal of CNSistent is to provide the user with tools for easy data processing so that SCNA profiles can be jointly used for downstream analysis. There are tools available that call CNs from various sequencing data and provide related visualizations in Python, e.g. CNVKit[31] or Segmentum[32], with many more outside Python, with comparison studies done e.g. by Masood et al.[33]. On the analysis side, there are many well known tools for detection of regions of interest, in particular GISTIC[34] and BISCUT[35], which take SCNA profiles and combine them, however, this is done internally by the tool and not accessible or controllable by the user. To the best of our knowledge the only tool for integrative analysis of SCNA profiles is the web-based CNApp[14], which shares some of the functionality with CNSistent, in particular re-segmentation and calculation of profile statistics. However, CNApp is designed for analysis within a web dashboard, while CNSistent serves as a tool for

the integration of data before application of downstream tools. We did not fully compare the tooling to CNApp as the hosting was not available at the time of writing.

Using the filtered and combined datasets, we compared several segmentation methods in providing features for a cancer type classification task. We observed that the relationship between segmentation size and model accuracy is highly model dependent: the Random Forest model quickly started to overfit, while the Elastic Net improved near-linearly with the number of segments. In our best performing model, segmentations within the region of 5 Mb to 500 Kb performed quite equivalently, and also matched the results obtained when classifying based on the hand-picked list of COSMIC[15] cancer genes. We adapted the CNN and DNN3 models originally introduced in *Attique et al*[18] and showed superior performance. The comparison is however limited, since the original model parameters, source code and source dataset were not available at the time of writing this article. All models and the input dataset used in this study are available online (see Data and code availability section). The main purpose of the classification task in this work was to evaluate different segmentation sizes. We therefore assume that better performing models could still be developed by fine-tuning for a particular segmentation or cancer type.

To investigate whether the results are consistent between cohorts, we compared classification between NSCLC cancers in all three datasets and saw that the per-sample accuracy improved by combining these three studies when compared to classification of each of them separately. We saw that models trained on one dataset can be successfully applied to classify another, sometimes even outperforming the source dataset, demonstrating that the classifier generalizes well. We also saw that our model trained on the joint dataset had a better accuracy than the average of the individual models trained on the individual dataset. On the joint dataset our model also considerably outperformed the previous result of *Qiu et al.*[19]. We therefore conclude that it is worthwhile to aim to integrate datasets from heterogeneous sources.

To show the utility of sample integration in analysis, we investigated the NSCLC samples using statistical methods. We identified chromosome 3 as the region of interest, in particular in the context of LUSC, with a wide peak in the location of the SOX2 gene, a well-known actor in LUSC[36]. Using gene-based segments we conducted a statistical test to find the most differently altered genes between LUSC and LUAD, which are likewise all located on chromosome 3. Additionally, we used the normalized Manhattan Distances score to detect outlier samples, with our detected outliers showing the selection pattern of the other cancer, possibly hinting at either mislabelling or co-occurrence of both cancers in the outlier samples[37]. Arguably, these results primarily demonstrate the applicability of our method and warrant further detailed investigations. Future work might also focus on developing methods for within-sample comparison of segments, as well as between-samples and between-types distance calculations.

Acknowledgements

The results published here are in part based upon data generated by the TCGA Research Network: https://www.cancer.gov/tcga.

The authors would like to thank Tom L. Kaufmann, Cody B. Strange, Philipp G. Keyl, and Tom B. K. Watkins, Thomas J. Y. Kono, Laura Godfrey, and Daniel Schütte for their feedback.

Literature

1. Beroukhim, R. *et al.* The landscape of somatic copy-number alteration across human cancers. *Nature* **463**, 899–905 (2010).
2. Jamal-Hanjani, M. *et al.* Tracking the Evolution of Non–Small-Cell Lung Cancer. *N. Engl. J. Med.* **376**, 2109–2121 (2017).
3. Turajlic, S. *et al.* Tracking Cancer Evolution Reveals Constrained Routes to Metastases: TRACERx Renal. *Cell* **173**, 581–594.e12 (2018).
4. Pan, X. *et al.* Identification of the copy number variant biomarkers for breast cancer subtypes. *Mol. Genet. Genomics* **294**, 95–110 (2019).
5. Watkins, T. B. K. *et al.* Pervasive chromosomal instability and karyotype order in tumour evolution. *Nature* **587**, 126–132 (2020).
6. Kaufmann, T. L. *et al.* MEDICC2: whole-genome doubling aware copy-number phylogenies for cancer evolution. *Genome Biol.* **23**, 241 (2022).
7. Watkins, T. B. K. *et al.* Refphase: Multi-sample phasing reveals haplotype-specific copy number heterogeneity. *PLoS Comput. Biol.* **19**, e1011379 (2023).
8. Steele, C. D. *et al.* Signatures of copy number alterations in human cancer. *Nature* **606**, 984–991 (2022).
9. Drews, R. M. *et al.* A pan-cancer compendium of chromosomal instability. *Nature* **606**, 976–983 (2022).
10. Gabrielaite, M. *et al.* A Comparison of Tools for Copy-Number Variation Detection in Germline Whole Exome and Whole Genome Sequencing Data. *Cancers* **13**, 6283 (2021).
11. Kuipers, J., Tuncel, M. A., Ferreira, P. F., Jahn, K. & Beerenwinkel, N. Single-cell copy number calling and event history reconstruction. *bioRxiv* (2024) doi:10.1101/2020.04.28.065755.
12. Zhang, J. *et al.* The International Cancer Genome Consortium Data Portal. *Nat. Biotechnol.* **37**, 367–369 (2019).
13. Al Bakir, M. *et al.* The evolution of non-small cell lung cancer metastases in TRACERx. *Nature* **616**, 534–542 (2023).

14. Franch-Expósito, S. *et al.* CNApp, a tool for the quantification of copy number alterations and integrative analysis revealing clinical implications. *Elife* **9**, e50267 (2020).
15. Tate, J. G. *et al.* COSMIC: the Catalogue Of Somatic Mutations In Cancer. *Nucleic Acids Res.* **47**, D941–D947 (2019).
16. Harrison, P. W. *et al.* Ensembl 2024. *Nucleic Acids Res.* **52**, D891–D899 (2024).
17. Perez, G. *et al.* The UCSC Genome Browser database: 2025 update. *Nucleic Acids Res.* (2024) doi:10.1093/nar/gkae974.
18. Attique, H. *et al.* Multiclass Cancer Prediction Based on Copy Number Variation Using Deep Learning. *Comput. Intell. Neurosci.* **2022**, 4742986 (2022).
19. Qiu, Z.-W., Bi, J.-H., Gazdar, A. F. & Song, K. Genome-wide copy number variation pattern analysis and a classification signature for non-small cell lung cancer. *Genes Chromosomes Cancer* **56**, 559–569 (2017).
20. Yadav, S. & Shukla, S. Analysis of k-fold cross-validation over hold-out validation on colossal datasets for quality classification. in *2016 IEEE 6th International Conference on Advanced Computing (IACC)* 78–83 (IEEE, 2016).
21. Paszke, A. *et al.* Automatic differentiation in PyTorch. *NIPS 2017 Workshop on Autodiff* (2017).
22. Streck, A. Input dataset for CNSistent integration and feature extraction from somatic copy number profiles. Zenodo: https://zenodo.org/records/14677713 (2024).
23. Streck, A. Processed data for ‘CNSistent integration and feature extraction from somatic copy number profiles’. Zenodo: https://doi.org/10.5281/zenodo.15620292 (2025).
24. Streck, A. Deep Learning code for ‘CNSistent integration and feature extraction from somatic copy number profiles’. Zenodo: https://doi.org/10.5281/zenodo.14546762 (2024).
25. DOME Registry. DOME-ML. https://registry.dome-ml.org/review/59bcqzam2c. (2024).
26. Van Loo, P. *et al.* Allele-specific copy number analysis of tumors. *Proceedings of the National Academy of Sciences* **107**, 16910–16915 (2010).
27. Sondka, Z. *et al.* The COSMIC Cancer Gene Census: describing genetic dysfunction across all human cancers. *Nat. Rev. Cancer* **18**, 696–705 (2018).
28. Lander, E. S. *et al.* Initial sequencing and analysis of the human genome. *Nature* **409**, 860–921 (2001).
29. ICGC/TCGA Pan-Cancer Analysis of Whole Genomes Consortium. Pan-cancer analysis of whole

genomes. *Nature* **578**, 82–93 (2020).

30. Boumahdi, S. *et al.* SOX2 controls tumour initiation and cancer stem-cell functions in squamous-cell carcinoma. *Nature* **511**, 246–250 (2014).
31. Talevich, E., Shain, A. H., Botton, T. & Bastian, B. C. CNVkit: Genome-Wide Copy Number Detection and Visualization from Targeted DNA Sequencing. *PLoS Comput. Biol.* **12**, e1004873 (2016).
32. Afyounian, E., Annala, M. & Nykter, M. Segmentum: a tool for copy number analysis of cancer genomes. *BMC Bioinformatics* **18**, 215 (2017).
33. Masood, D. *et al.* Evaluation of somatic copy number variation detection by NGS technologies and bioinformatics tools on a hyper-diploid cancer genome. *Genome Biol.* **25**, 163 (2024).
34. Mermel, C. H. *et al.* GISTIC2.0 facilitates sensitive and confident localization of the targets of focal somatic copy-number alteration in human cancers. *Genome Biol.* **12**, R41 (2011).
35. Shih, J. *et al.* Cancer aneuploidies are shaped primarily by effects on tumor fitness. *Nature* **619**, 793–800 (2023-7).
36. Wuebben, E. L. & Rizzino, A. The dark side of SOX2: cancer - a comprehensive overview. *Oncotarget* **8**, 44917–44943 (2017).
37. Zhang, T., He, R., Xiao, Y. & Geng, Q. Primary squamous cell carcinoma and adenocarcinoma simultaneously occurring in the same lung lobe: a case report and literature review. *Front. Oncol.* **14**, 1402297 (2024).

Supplementary Figures 1-10

Click here to access/download
Supplementary Material
Supplementary Figures CNSistent.pdf

Supplementary Table 1

Click here to access/download
Supplementary Material
supplementary_table_1.xlsx

Supplementary Table 2

Click here to access/download
Supplementary Material
supplementary_table_2.xlsx

Supplementary Table 3

Click here to access/download
Supplementary Material
supplementary_table_3.xlsx

 Click here to access/download;Personal Cover;CNSistent_Reponse_To_Reviews_2.pdf

Reviewer #1:

The only comment I'd make is with point 6. Metadata effects: Age influences the copy number alterations. The authors don't consider age or any other metadata and their implication in the classification task.

I agree with the authors that controlling for age is non-trivial. However, explicitly including it as a covariate could introduce collider bias (i.e if age is correlated with a specific cancer type it will be highly predictive even if it's effect on copy number profiles are trivial). The commentary around predictive accuracy is also not that useful as (for e.g) age might be highly predictive for one of the classes but not the others- accuracy here will not reflect this.

In some ways if prediction is the end goal it doesn't really matter if CNA profiles have age signatures that improve this prediction. Of course, if interpretation is the end goal, then understanding age specific factors is important. In this case, probably what would be required is some sort of model that predicts copy number at each genomic window- with a cancer type specific age covariate, a global age covariate and a cancer type covariate. Probably the age covariates would be shared across all genomic windows (or maybe could be chromosome specific but likely window specific would be too fine grained). This could be interesting future work to explore but is not necessary for this work.

We would like to thank Reviewer #2 for taking on the responsibility of also taking care of the responses to the comments from Reviewer #1. To observe if there is indeed not a trivial bias introduced through age as suggested by the reviewer, we have tested 1-vs-all binary linear classifiers on balanced datasets (also now included with the code) for the top 6 cancer types we use in classification, i.e. for each class we selected all the samples in the class, and a random subsample of the remaining classes of the same size and attempted to distinguish between the two using age as the only classification variable. The results were the following:

```
KIRC: Accuracy = 0.5278
OV:   Accuracy = 0.5934
BRCA: Accuracy = 0.5972
LUAD: Accuracy = 0.5930
LUSC: Accuracy = 0.6154
PRAD: Accuracy = 0.5764
```

These data suggest that age is not highly predictive of any specific cancer type in our cohort. Looking at the individual distributions we can see that the only apparent distinction is between lung cancers and the rest, as seen on the reviewer figure, which we reproduced below:

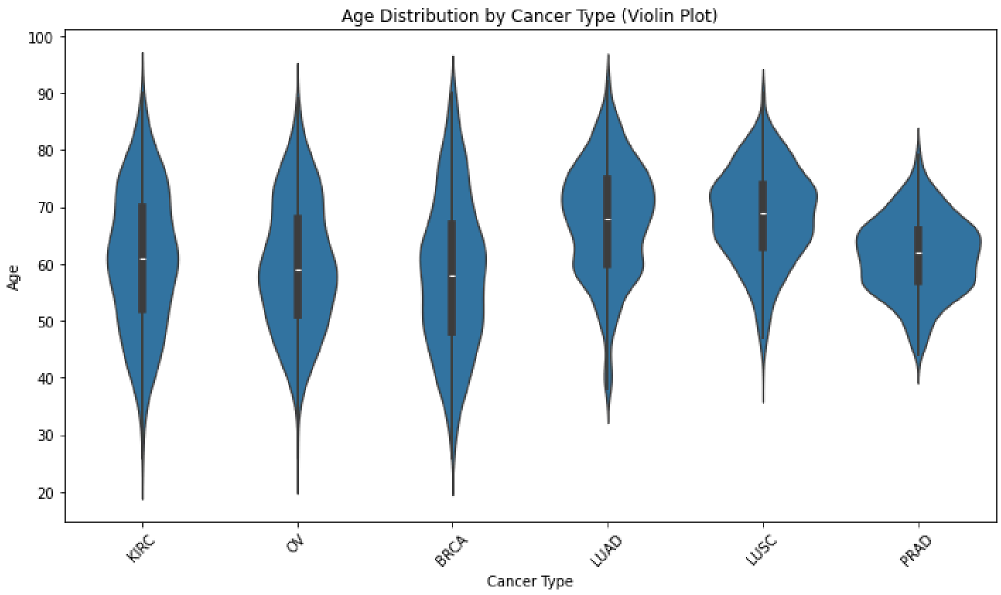

We therefore do not believe age to be a direct confounder in our case.

We also investigated the relationship between age and copy number alterations in more detail, using the number of breakpoints in a sample as a proxy for the number of structural variants. If there was a strong effect of age on the copy number landscape, we should see a correlation between the number of breakpoints and age, however we did not find a strong correlation in the 12009 samples tested. We have added the result of this analysis as a new Supp. Figure 7, reproduced here:

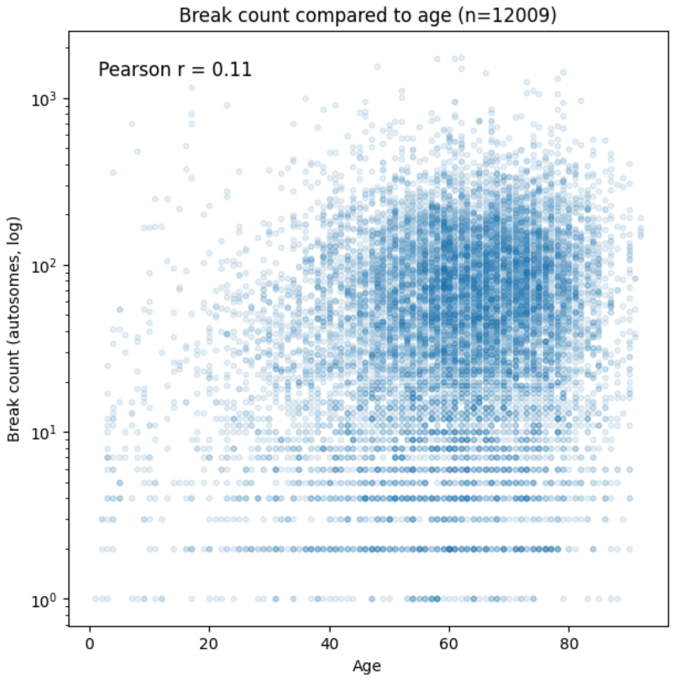

To reflect these thoughts we have inserted the following paragraph in the Results section:

"We only considered segments on autosomes as the sex of the patient acts as a confounder, in particular for BRCA, OV, and PRAD. To evaluate any possible confounding effect of age we compared the number of breakpoints to the age of the patients across the cohort (n=12009), and found it to only have a minor effect (r=0.11, Supp. Fig. 7). Similarly, age alone was a poor predictor of cancer type, achieving a mean test accuracy on a class-balanced one-versus-all linear classifier of 59.03% and a validation accuracy for the multi-class linear classifier of 31.78%."

The above is of course an analysis of a linear relationship between the age and the alterations. Non-linear relationships are likely to exist, although it is not clear to us to what extent, however we felt that a deeper analysis of the topic goes beyond the scope of the article.

Reviewer #2: The authors have done a great job of addressing reviewer comments, adding further analysis and making the manuscript clearer.

(Very) Minor comments:

"To conduct such analyses however SCNA profiles have to be integrated between samples, patients, and cohorts, an often non-trivial task, for which dedicated toolkits are lacking." - This abstract sentence is clunky/poorly punctuated.

We have changed the word order to the following: *However, to conduct such analyses SCNA profiles have to be integrated between samples, patients, and cohorts—often a non-trivial task, for which dedicated toolkits are lacking.*

"The following was not declared in the manuscript and therefore has been set to default PyTorch values" - maybe change this to directly reference the manuscript in question (i.e Attique et al?)"

As suggested, we have changed the sentence to: *The following was not declared in Attique et al.[18] and therefore has been set to default PyTorch values.*

"We have introduced CNSistent, a new Python-based library for processing and exploratory data analysis of SCNA profiles. Using the PCAWG, TCGA, and TRACERx datasets." Punctuation

We thank the reviewer for spotting the issue. We have corrected the text to the following: *We have introduced CNSistent, a new Python-based library for processing and exploratory data analysis of SCNA profiles and applied it to the PCAWG, TCGA, and TRACERx datasets.*

Discussion, suggest for:

Unfortunately, the comparison of our models to the one from Attique et al18 was partially limited by the fact that the authors did neither provide all of the model parameters, nor the model source code, and that the source dataset was no longer available at the time of writing this article. Validation on a hold-out set or cross-validation has not been conducted by the authors, we therefore only performed our comparisons on the test accuracies. All models and the input dataset used in this study are available online (see Data and code availability section).

Something like:
"We adapted the CNN originally introduced in Attique et al, and showed superior performance, with the caveat that the original model parameters, source code and source dataset were not available at the time of writing this article. All models and the input dataset used in this study are available online (see Data and code availability section)." And change the location of "Validation on a hold-out set or cross-validation has not been conducted by the authors, we therefore only performed our comparisons on the test accuracies." to be in the results section, where you compare to their results directly.

We agree with the reviewer and we have updated the suggested sentence with minor modifications. The updated Discussion now contains: "*We adapted the CNN and DNN3 models originally introduced in Attique et al*[18] *and showed superior performance. The comparison is however limited, since the original model parameters, source code and source dataset were not available at the time of writing this article. All models and the input dataset used in this study are available online (see Data and code availability section).*"

The second part we placed in the Results section as follows: *"Validation on a hold-out set or cross-validation has not been conducted by the authors, we therefore only performed our comparisons on the test accuracies. The best test accuracy (maximum of 5 folds) was 92.42% on 1 Mb segments with the CNN+ mode, slightly above the best test accuracy of Attique et al. (92%)."*

Typo/grammar:
"which likewise all additionally we used a normalized Manhattan Distances score to detect outlier samples"

We thank the reviewer for noticing the error, we corrected the text to the following: …*which are likewise all located on chromosome 3. Additionally, we used the normalized Manhattan Distances score to detect outlier samples…*

Reviewer #3: The revised is improved and much more readable. No further comments.

Cover Letter

Click here to access/download;Personal Cover;20250608_CNSistent_Revision_v2_CoverLetter_rfs.pdf

University of Cologne
Medical Faculty

ICCB • https://iccb-cologne.org • @rfschwarz

Institute for Computational Cancer Biology

Prof. Dr Roland Schwarz

Phone: +49 221 478 51465

roland.schwarz@iccb-cologne.org
https://iccb-cologne.org

GigaScience
Editorial Board
Qing Lan

Cologne, 30/07/2025

Dear Editorial Board Members, dear Mr. Lan,

Please find attached the revised version of our manuscript titled: **"CNSistent integration and feature extraction from somatic copy number profiles"**. We were very pleased to hear that you enjoyed reading our article and that you found it to be potentially acceptable for publication in GigaScience.

We have now addressed all the remaining reviewers' comments and you find the revised manuscript attached to this submission.

The **only remaining major comment** was about the **role of age as a potential confounder in predicting cancer type**. We now provide additional evidence that age does not to any major degree influence the cancer type classification task and provide additional statistical analyses and reviewer figures to underline that point.

As before, the revised manuscript version is accompanied by a point-by-point response in PDF format with additional Figures for the reviewers' convenience. Our responses and all updated text in the manuscript are marked in blue.

We hope that you and the reviewers are satisfied with the changes we made. We feel that the article has overall strongly benefited from the review process and we would like to thank the reviewers and you for your time and effort.

Yours sincerely,

R. Schwarz

Roland Schwarz

ICCB Institute for Computational Cancer Biology
info@iccb-cologne.org
www.iccb-cologne.org

Institute for Computational Cancer Biology
University of Cologne
Gebäude 66 (TRIO)
Robert-Koch-Straße 21
50931 Cologne
Germany

UNIKLINIK KÖLN
