# Supplementary material for: CNSistent integration and feature extraction from somatic copy number profiles
Source: Gigascience. 2025 Sep 13;14:giaf104. doi: 10.1093/gigascience/giaf104 (PMC12448185; doi:10.1093/gigascience/giaf104)
Supplement: giaf104_Authors_Response_To_Reviewer_Comments_Original_Submission [file giaf104_authors_response_to_reviewer_comments_original_submission.pdf]

# Author's Response To Reviewer Comments

Below are our itemized responses to reviewers in plain text. For a formatted text with included reference graphics, please refer to the document: "Revision - Responses to Reviewers"

Reviewer #1:
This is a well-written paper that aims to develop a tool that can integrate SCNA from large datasets possibly generated using different platforms to identify alteration patterns that are often undetected in smaller data subsets. Authors have used CNN-based method for integrating the data, extracting features and predicting cancer types from SCNA profiles. The tool has the potential to significantly simplify the integration and analysis of large scale SCNA studies. However, some (hopefully addressable) weaknesses are noted:

1. The choice of a classification task as the (only) way to evaluate the proposed method is questioned. I would argue that the most important use of SCNA detection is in support of mechanistic investigations, by identifying novel candidate loci likely to harbor tumor suppressors (copy losses) and oncogenes (copy gains). This type of analysis is hardly mentioned in the manuscript, and it is not clear how well the proposed tool would support it. I surmise it can, but the authors should discuss (and present results about) it.

Response: We agree with the reviewer that a classification task is not the only and arguably not the ideal way of demonstrating the power of CNSistent. CNSistent will prove useful every time CN profiles have to be integrated between samples from the same patient, between patients or between cohorts. Peak detection certainly is one such example. Since GISTIC uses its own internal integration method for CN profiles, it is not ideally combined with CNsistent.

To follow the reviewer's suggestion, we thus implemented our own simple peak detection algorithm, described in the Methods section Peak Detection: "To find regions of interest in the samples, CNSistent provides the peak score (PS), which shows how much each bin differs from its neighbours. Have an aggregated sample S = (s1,,sn) we set the boundary values s0 = s1, sn+1 = sn and calculate i (1,, n): PS(S, i) =(si - si-1) -(si+1-si). This score will be positive for segments higher than their neighbours, negative for those lower and close to zero for segments with monotonous behaviour. We therefore use the PS to detect the highest and lowest values, which show the locations of most abrupt change in CN accumulation. Note that for meaningful calculation this requires that the segments are connected to each other and about the same size."

To illustrate the flexibility of CNSistent we applied this simple algorithm to the LUSC samples with varying segmentation sizes from 500 KB to 20 MB. We observe that the top 3 peaks vary between segment sizes, with chr3 being detected mostly in big segments due to a wide slope on the q-arm, while chr8 is being mostly detected in middle sizes due to amplification at the end of the p-arm (new Fig. 4A).

Based on the reviewer comments, we have also removed the Integrated Gradients method in favour of a statistical test in the Results, which reads: "To systematically determine which genes differ significantly in their CNs between LUAD and LUSC, we conducted a Mann-Whitney U-Test with Benjamini-Hochberg correction on the mean CNs of the COSMIC genes. Out of 722 genes, 599 had adjusted p-value below 0.05. The top 5 most copy number altered genes were all on the q-arm of chr3 (Fig. 4D). All these had the adjusted p-value below 10-169, with SOX230 being the most significant at p10-187. The SOX2 gene also has the highest mean CN of 7.56."

To further illustrate the broad applicability of CNSistent we now provide phylogenetic inference from somatic copy number profiles as an additional example. To this end we leveraged samples from the TRACERx cohort as described together with our phylogenetic inference tool MEDICC2 (cite Kaufmann et al. 2022). Before any phylogenetic analysis can be carried out CN profiles from the same patient have to undergo joint segmentation to make them comparable by an evolutionary model. Figure 4E now shows the CN profiles from TRACERx case CRUK0001 aligned with CNSistent as well as the phylogenetic tree inferred

by MEDICC2.

2. If we were to focus on the task of recurrent SCNA detection, then meta-analysis approaches (where separate analyses are performed on each of the datasets, and only the results are integrated) would need to be considered as an alternative to the approach here proposed (e.g., application of GISTIC to each of PCAWG, TCGA, TRACERx separately, followed by meta-analysis integration of the results). I am not saying meta-analysis would be superior, but the authors should discuss it, and possibly evaluate it.

Response: We agree with the reviewer that meta-analysis techniques can be an alternative to CNSistent in some situations. For example, simple gene-level aggregations of CN states might also be possible with ad-hoc implementations. However, for larger segment sizes or as soon as whole-genome CN profiles are considered, technical differences between samples, patients or cohorts, for example with respect to missing data or blacklisted regions will require algorithmic design decisions that are not trivial. CNSistent provides such tools to enable reproducible processing of large cohorts in a unified manner.

Unfortunately, a direct comparison with GISTIC is not possible, since GISTIC uses its own internal integration strategy. However, as described in response to this reviewer's comment #1, we have implemented our own simple peak detection algorithm to illustrate the use of CNSistent for this purpose and would refer the reviewer to the above comment for details. We have also added a paragraph in the Discussion to clarify this point. It now reads:

"On the analysis side, there are many well known tools for detection of regions of interest, in particular GISTIC34 and BISCUT35, which take SCNA profiles and combine them, however, this is done internally by the tool and not accessible or controllable by the user."

To demonstrate the increase in power in combining many patients or cohorts we show on the task of NSCLC classification that training on TRACERx and applying the results to PCAWG provides better results than training on PCAWG itself. To make this clearer in text, we have changed the Methods to the following:

"To demonstrate the potential of integration using CNSistent across different datasets. For the binary lung cancer classification task we have trained the models on one dataset and tested on another (Fig. 3E). We see that the results transfer well, with up to 93.90% accuracy for the TRACERx model applied to PCAWG. We also see that compared to self-training, the models trained on bigger sets (TRACERx, TCGA) outperform self-training on the small PCAWG model. Likewise, training on TRACERx slightly outperforms self-training on TCGA. The 5-fold cross-validation accuracy on the combined dataset was 92.12%, considerably improving on the previous result of 84% in Qui et al.(Qiu et al. 2017)."

3. The reported metrics to quantify the quality of the integration are insufficient to assess the results. There is some lack of clarity about the classification accuracy results reported, since it is not clear whether all the components of the model building were adequately brought into the cross-validation (or train/test) loop. More specifically, when reporting the accuracy of the cancer type classification, it is reported that 1 megabase segmentation yields the best results. It is not clear if this size selection was performed within the train set only (and/or within the CV loop) or across the entire dataset. If the latter, this may significantly affect the accuracy results, which could not be deemed (unbiased) "test set" results. This should be clarified, and if the segment size selection was indeed performed outside the train/test split, accuracy measures should be computed again by performing the segment size selection properly (which of course it would mean a potentially different size would be selected for each of the folds).

Response: We thank the reviewer for raising this point, which we hopefully can clarify. We here aimed to compare the different segmentation strategies, rather than to select the best model. The split into the 5 folds is the same for each segmentation strategy and the accuracy has been averaged across the 5 folds in order to best demonstrate how well a method would perform on such a segmentation. We are aware that using this method makes the nomenclature somewhat confusing, since the validation accuracy is the mean of the test accuracies. We have aimed to address this by including a new paragraph in the Machine learning subsection:

"To obtain scores of individual models on the individual datasets, we use 5-fold cross validation, i.e. we

split each dataset into 5 groups and always withheld one while training on the other 4. The validation accuracy for each model/size combination is then the mean of the 5 different splits (Yadav and Shukla 2016)."

We use this validation technique rather than train-test-validation split because i) the dataset is quite small, ii) we mostly aim to compare between different options and averaging training results prevents possibility of favorable selection of validation set for a particular method.

The segmentation strategies are pre-selected and tested independently, i.e. there's no segment size selection during the train/test process, we just report the results of all the options. We tried to make the segment size selection process clearer with the following paragraph in Results:

"We next set out to explore the effects of different segmentation strategies on the cancer classification task (see Methods). We processed the data using the following segmentation strategies: (i) fixed-size segments of 20 Mb, 10Mb, 5 Mb, 3 Mb, 2 Mb, 1 Mb, 500 Kb, 250 Kb, 100 Kb size; (ii) Whole chromosomes, chromosome arms; (iii) Gene-level CN values based on the ENSEMBL and COSMIC gene sets; and (iv) breakpoint merging using distance thresholds of 1 Mb, 500 Kb, and 250 Kb. The segment sizes roughly cover the ranges used by other authors for feature discovery(Drews et al. 2022)"

4. Comparisons with other methods: The authors only compare their method to random forest (RF). Related to the previous point: I presume the RF model used the segment size that was optimized for the CNN model (i.e., 1Mb). If this is the case, it would be an unfair comparison, since RF might favor a different size. Also, additional classifiers should be evaluated (e.g., Elastic Net, SVM, etc.).

Response: We have now calculated the performance of the RF classifier across all segment sizes and added an additional linear classifier with Elastic Net regularization for comparison. We decided against inclusion of a SVM as the performance of this kernel-based classifier would strongly depend on the kernel and hyperparameters used.

While our original neural network was very close to the architecture used by Attique et al., it included an additional linear layer. We therefore now reconstructed the DNN3 and CNN of Attique et al. to the best of our ability based on their manuscript and included our model as an additional one, called CNN+.

The corresponding text in the results section reads:

"We compared the DNN3 and CNN architectures of Attique et al., Random Forest, Elastic Net, and our extension of the CNN architecture–CNN+ (Fig. 3B)–across decreasing segment sizes. On whole chromosomes the most performing models reached a validation accuracy of ~80% and considering the arms separately reached almost ~86% on the DNN architecture. Increasing the resolution improves accuracy of the two convolutional models, which peak in the region of around 1000 segments. The best validation (mean of 5 folds) accuracy of 90.60% was achieved with the CNN+ at 1 Mb segments, which we used for the subsequent tasks, however sizes from 5 Mb to 250 Kb all had validation accuracy above 90%, and we would therefore consider all of them to be suitable for any further analyses.

The RF and DNN models however peak around 200 segments and increasing the resolution further decreases the validation performance, likely due to overfitting. The only architecture that improved monotonously was ENet, where the penalty regularization seemed to prevent overfitting, however from 20 Mb onwards it always underperforms compared to the CNN+. Comparing the full segmentation with the COSMIC and ENSEMBL gene sets we saw that taking only the CNs for genes performs equivalently to creating a segmentation with a similar number of features."

5. There is no sufficient discussion of existing tools/methods. This should be corrected (see also my comment about meta-analysis approaches).

Response: To better situate CNSistent in the field and to provide a better overview of existing alternative tools, we have added the following paragraph to the Discussion:

There are tools available that call CNs from various sequencing data and provide related visualizations in Python, e.g. CNVKit31 or Segmentum32, with many more outside Python, with comparison studies done e.g. by Masood et al.33. On the analysis side, there are many well known tools for detection of regions of interest, in particular GISTIC34 and BISCUT35, which take SCNA profiles and combine them, however, this is done internally by the tool and not accessible or controllable by the user. To the best of our knowledge the only tool for integrative analysis of SCNA profiles is the web-based CNApp14, which shares some of the functionality with CNSistent, in particular re-segmentation and calculation of profile statistics. However, CNApp is designed for analysis within a web dashboard, while CNSistent serves as a tool for the integration of data before application of downstream tools. We did not fully compare the tooling to CNApp as the hosting was not available at the time of writing.

6. Metadata effects: Age influences the copy number alterations. The authors don't consider age or any other metadata and their implication in the classification task.

Response: We thank the reviewer for pointing this out. To evaluate if age might act as a confounding in our cancer type classification task we investigated the predictive power of age on the cancer types considered. A simple Linear classifier on the top 6 types showed a test accuracy of 0.322, indicating that age alone is not a strong predictor of cancer type and thus is unlikely to confound our classification results.

Unfortunately, we were not able to obtain age values for ~10% of the samples. In the interest of manuscript length, we have decided not to include this analysis in the text but naturally would be happy to do so if the reviewer feels strongly about this.

7. Run time statistics and user requirement: While the authors report runtime curves per command (S Fig 6), it is difficult to translate this to total runtime. It would be useful if runtime for the entire training of a model were reported. Additionally, if available, comparison of run time stats with the established model that they cite would be useful.

Response: We have reproduced two of the existing architectures and compared the times to our model, Random Forest and Elastic Net.

8. IG-based explanation. I found this section sort of perfunctory, not sufficiently justified, and adding little to the manuscript. IG is computationally expensive, and it does not provide any way to statistically quantify the found associations. Simpler methods, such as testing for association between SCNA occurrence and cancer type should be evaluated and compared to.

Response: We originally used IGs as a preliminary exploration of points of interests, but we agree with the sentiment concerning the computational costs. We have now completely removed the IG section and instead replaced it with a simpler section focused on a statistical test for association between cancer type and the prevalence of copy number changes.

Briefly, we applied our Peak Score to 1 Mb segments. This detected the peak in the vicinity of SOX2 on chromosome 3 and the focal amplification at the p-arm of chromosome 11 near the centromere, matching the results of the application of IGs to Segments.

We then conducted Mann-Whitney U-Test on the COSMIC gene sets comparing the LUAD-LUSC sample sets. The set of the 5 most statistically significant differences between the genes and the set of 5 genes with the highest IG score have both on 3 of the genes: SOX2, PIK3CA, and TBL1XR1.

We therefore conclude that these much simpler and more explainable methods suit as a good replacement of our previous IG-based analysis.

For details, please see the updated section: Identifying commonly altered regions and outliers .

9. Model selection: No adequate justification of why they picked CNN for this task when the referenced paper itself claims the DNN architecture performs better. Not sure but is this because of the varying segment size? Again, this is not clearly stated. https://pmc.ncbi.nlm.nih.gov/articles/PMC9203194/#tab1

Response: We have attempted to reconstruct the models of Attique et al. as faithfully as we could based on the manuscript, however there were limitations, which we summarized at the end of the Discussion:

“Unfortunately, the comparison of our models to the one from Attique et al18 was partially limited by the fact that the authors did neither provide all of the model parameters, nor the model source code, and that the source dataset was no longer available at the time of writing this article.”

In the methods we specify that the Dropout probability and MaxPool kernel size are not declared. Additionally, it was not clear what the layer sizes for DNN5 were. As DNN3 has already been overfitting in our implementation, we have not included the DNN5. Also the declared test accuracies of DNN3 and DNN5 were 91% and 92% respectively, therefore we have not felt that there was a meaningful difference.
Reviewer #2:
The paper introduces a python package for imputation, filtering, segmentation, feature extraction and visualisation of CNA profiles. It explains some of the elements of the package, and then demonstrates how data from multiple cohorts can be processed and combined using the package preprocessing pipeline. The authors then use processed data from 3 different cohorts to perform cancer type prediction using a CNN. From this, they get an interesting result to find a biomarker that differentiates two different lung cancers. Throughout, they show visualisations using their package. The package itself seems well documented and designed to be used. There is some clarification required in the methods section specifically around the CNN training and the models therein. There is also one major question of whether all the preprocessing steps are actually required for the downstream CNN analysis. Overall, however, this is a well written manuscript, providing a useful
software tool for further analysis of CNA data.

Major comments:
- CNN section- how are the segments decided- is it based on all the training data, or just data in a batch?

The segmentation strategies are pre-selected and tested independently, i.e. there’s no segment size selection during the train/test process, we just report the results of all the options. We tried to make the segment size selection process clearer with the following paragraph in Results:

“We next set out to explore the effects of different segmentation strategies on the cancer classification task (see Methods). We processed the data using the following segmentation strategies: (i) fixed-size segments of 20 Mb, 10Mb, 5 Mb, 3 Mb, 2 Mb, 1 Mb, 500 Kb, 250 Kb, 100 Kb size; (ii) Whole chromosomes, chromosome arms; (iii) Gene-level CN values based on the ENSEMBL and COSMIC gene sets; and (iv) breakpoint merging using distance thresholds of 1 Mb, 500 Kb, and 250 Kb. The segment sizes roughly cover the ranges used by other authors for feature discovery(Drews et al. 2022)”

- Throughout the results pertaining to figure 3A-C, you call it test accuracy- to be clear is this is based on your CV hold outs? This should be reworded everywhere to reflect this. As cross validation indicates, this is not a test set and is a validation set- which is also the way you use it.

We thank the reviewer for raising this point, which was also raised by Reviewer 1. In essence, we aimed to compare the different segmentation strategies, rather than to select the best model. The split into the 5 folds is the same for each segmentation strategy and the accuracy has been averaged across the 5 folds in order to best demonstrate how well a method would perform on such a segmentation. We are aware that using this method makes the nomenclature somewhat confusing, since the validation accuracy is the mean of the test accuracies. We have aimed to address this by including a new Methods paragraph in the text as shown below:

“To obtain scores of individual models on the individual datasets, we use 5-fold cross validation, i.e. we split each dataset into 5 groups and always withheld one while training on the other 4. The validation accuracy for each model/size combination is then the mean of the 5 different splits (Yadav and Shukla 2016).”

- Regarding the above, you have a comment saying: "the best test accuracy without cross-validation was

92.34%". Could you please clarify what you mean by this. Only in the CNN section do you describe your training approach, which does not mention a test or separate validation set.

Response: We hope the validation approach has been made clearer by the above paragraph. The test accuracy of 92.34% comes from the fact that Attique et al. did not conduct validation, therefore we can only compare to their test scores. However, we believe the validation score to be a more informative metric of classification performance and therefore chose to work with that number instead. We have updated the Results to reflect this as follows:

“The best test accuracy (maximum of 5 folds) was 92.42% on 1 Mb segments with the CNN+ model. This is slightly above the best test accuracy of Attique et al. (92%).‟

This is further discussed in the Discussion:

“Validation on a hold-out set or cross-validation has not been conducted by the authors, we therefore only performed our comparisons on the test accuracies.‟

- It reads slightly unclearly- you have a section called "model transfer", but are you training 3 different models- one per dataset? You only have one figure for training results which suggests one dataset, but then you have this section called model transfer?

Response: We agree with the reviewer that our terminology might have been confusing and have removed the term “transfer” altogether. We have indeed trained 3 different models, each on one dataset and applied it independently to the remaining two, creating 6 combinations, where one dataset is the training set and the other is the validation set. We have then compared these to the results of the 5-fold cross validation on each individual dataset. We have generally scaled this section back in line with the decrease of focus on deep learning and tried to express the process as clearly as possible in the Results:

“To demonstrate the potential of integration using CNSistent across different datasets we used the NSCLC classification task, training the models on one dataset and validating on another (Fig. 3E). We see that the accuracies of models trained on a different dataset match or sometimes even outperform models trained and validated on the same dataset, with up to 91.46% accuracy for the TRACERx model applied to PCAWG. We also see that compared to self-training, the models trained on bigger sets (TRACERx, TCGA) outperform self-training on the small PCAWG model. Likewise, training on TRACERx slightly outperforms self-training on TCGA. The 5-fold cross-validation accuracy on the combined dataset was 92.73%, considerably improving on the previous result of 84% in Qui et al. When training the models individually, we obtain only 91.21% mean validation accuracy, showing that combining the datasets leads to a (1.52%) improvement.”

- Re all the above, please dedicate a small subsection in methods making this clearer. Are there dedicated test sets? If your main results are for aggregated data, then what are you testing on to ensure generalisability? What is the point of training the 3 different models on 3 different datasets? Perhaps it would make more sense to hold one dataset out as your test set. In some ways, that is what the model transfer is showing, but it would be less confusing to clarify that aim instead of suddenly introducing 3 models.

We have now updated the Methods section to accommodate the comments by the Reviewer as indicated in detail in the answers above, to which we would kindly refer you here.

Regarding the question about the three models, we use this to test whether classifiers can be transferred from one cohort to another, similar to a train/validation split, i.e. we hold out the remaining two cohorts and train only on one cohort with the remaining two used to obtain two validation scores. We hope that this is also clearer from the text quoted above.

- If the CNN architecture is essentially the same as in Attique et. al., the performance is basically the same and they use only CNs a gene locations- how does this demonstrate that the preprocessing from CNSistent is necessary or advantageous for this task? Maybe having a result which combines CN calls naively over

gene locations and comparing to this across the aggregate datasets would be a good way of comparing? I.e showing that preproccessing does offer an advantage when combining different datasets together? Also because this is what you argue in your abstract. For this analysis you would have to make sure you also compare across the same samples to differentiate between filtering/other preprocessing steps.

Response: We agree with the reviewer that aggregation or meta-analysis techniques can be an alternative to CNSistent in some situations. For example, simple gene-level aggregations of CN states might also be possible with ad-hoc implementations. However, for larger segment sizes or as soon as whole-genome CN profiles are considered, technical differences between samples, patients or cohorts, for example with respect to missing data or blacklisted regions will require algorithmic design decisions that are not trivial. CNSistent provides such tools to enable reproducible processing of large cohorts in a unified manner.

To demonstrate the increase in power in combining many patients or cohorts we show on the task of NSCLC classification that training on TRACERx and applying the results to PCAWG provides better results than training on PCAWG itself, as discussed in the paragraph above.

Furthermore, we have added a comparison of filtered (pre-processed) and non-filtered (all available) sample sets in Figure 3C, which shows clear and consistent improvement after the filtering process:

- In Figure 3I, you say "notice the similarity of chromosome 3 pattern for the correctly classified LUSC samples (red) and the misclassified ones (orange)". This is confusing because the orange and red are not similar. In fact for this whole section, it seems that figure 3I does not align with what you are saying?

Response: We apologise for this mistake on our part. There was indeed an error in Figure 3. However, in Response to Reviewer 1 comment #8, we have now removed the IG analysis from the paper and replaced it with a statistical association test. This also removes Figure 3l. We reproduce the new Results paragraph and corresponding Figure 4C here for your convenience:

“We then calculated the outlier score between LUAD and LUSC and used the kneepoint detection to find an outlier threshold (Supp. Fig. 8), finding 3 LUAD samples with LUSC-like pattern (amplification of SOX2) and 54 LUSC samples with neutral LUAD-like pattern (Fig. 4C). We also observed that the majority of these samples (58.75%) came from the TCGA dataset, while the TRACERx dataset had the least outliers (15.79%).”

Minor comments/errors:
- Clarification on why CNSistent needs a reference genome if it's dealing with segments? How is this information used- is it just for the known gaps?

Response: The reference genome is needed to know the expected chromosome lengths for the imputation as described in the Segment Imputation subsection “(ii) CNSistent extends the first and last segment of each chromosome to the chromosome boundaries”. CNSistent also provides built-in segmentation based on cytobands (listed as option in Consistent Segmentation subsection), whose locations are likewise taken from the reference.

- Your caption of Supplementary Figure 1 has a typo about a breakpoint at 16 instead of 14.

Response: Thank you for noticing, we have fixed the typo.

- You do not explain how you use the knee pt to filter (i.e is it samples above/below the knee pt.)

Response: “We used individual thresholds to filter the samples” changed to “We used individual thresholds to remove samples whose values were strictly smaller than the threshold”
- Your CNN graphic is difficult to interpret and non-standard.

Response: We have updated the figure to more closely match the standard visual scheme for CNNs and updated the figure caption:

Supplementary Figure 3: The CNN+ model of auto-scaling 1D convolutional neural network. The input layer I has size |I| and the output layer O has the size |O|, corresponding to the number of classes. The example is visualized for the case of 6-type classification (|O|=6) on filtered chromosome arms (|I|=40).

- CNN section should clarify at the beginning what the input is and what the output is (i.e a prediction that a sample belongs to a particular cancer type) before explaining the architectural details.

Response: We have added the following to the Machine learning subsection to clarify: “In this task, each binned sample as illustrated in Fig. 1 represents one feature vector. The output probability that a sample belongs to each cancer class under consideration.”

- Even though you control for class imbalance, some cancer types are so poorly represented it is unlikely a CNN could learn that, you do kind of mention this in the discussion, but maybe some sort of minimum threshold for inclusion would make sense.

Response: Thank you for the suggestion, we agree with the point and we have hence limited the classification to only classes with at least 100 samples. The supplementary figure has been updated to reflect this:

- For Fig2D you refer to it as GND, but the axes/title says hemizygosity-are these things equivalent? E.g could have 3-3, low hemizygosity but not diploid? Or if it's aggregated across the whole genome its assumed equivalent?

Response: Thank you for spotting the discrepancy. The x-axis label and title were supposed to read GnD, which we have now corrected.

- There is a grammatical error "Runtimes decreased in a near-linearly with the number of compute cores"

Response: Thank you for spotting the mistake, we have corrected it to: “Runtime decreased in a near-linear fashion with the number of compute cores available.”

- You make a comment that "We therefore suspect some TCGA lung cancers might be cases of co-occurring adeno and squamous carcinomas." This is a possibility but given pleiotropy of many phenotypes- it may also be that the biomarker is not always unique to squamous carcinomas.

Response: We have updated our analysis using the outlier detection method where the SOX2 amplification shows a considerably clearer pattern (see below), however it is still a hypothesis and we cannot confirm or reject it from copy numbers alone.

Suggestions/Nice to haves:
- Maybe make it clearer inside the paper what visualisations come with CNSistent. Looking at the software documentation, there's obviously a lot of useful visualisations that come with that- and some of them you have used in Figure 3 for e.g.

Response: All of the plots in Figure 4 are now produced by CNSistent. The label also states that explicitly: “Except for the phylogenetic tree, all plots are produced using CNSistent plotting functions.”

- Given there are more total CN callers, maybe good to mention somewhere how CNSistent would work for total CNs only.

Response: We have added the following sentence to the methods: “The processing is the same for both allele-specific and total copy numbers, however some of the statistics are limited in the case of copy numbers, as detailed below.”

- You remove profiles that you say are uninformative, could you not include this and then just show how accuracy correlates with no. of break-pts (for e.g). In some ways one might think that there could be useful information in few alteration profiles- because those alterations might be more upstream/causal.

Response: We hope that the Figure 3C demonstrating the difference between filtered and unfiltered samples demonstrates the benefit of filtering:

We preferred to display this on our filtering results rather than on the breakpoint count as we don't see breakpoints as we tried to argue for the GnD as an evidence of SCNAs rather than a breakpoint count, as we argued in Fig. 2E and the related text:

"Other authors have used the number of breakpoints9 as evidence for SCNAs, however we have not observed a clear knee-point in the data (Fig. 2E) and any threshold would therefore be arbitrary."

- The aggregation step could maybe affect downstream analysis. I.e taking the average could introduce CNs that were never called. Even using min/max- this implies a constant copy number in that region, which may lose information- e.g if it is a functional region having two diff CNs across gene might imply non-functionality. Did you explore the effect of aggregation step? Perhaps taking a small enough resolution of segment types would account for this anyway.

Response: We have added a 100 Kb resolution, which is still about 30-times smaller than what a full minimum consistent segmentation would be, however even there we see a trend of flattening training accuracy and decreasing test accuracy, therefore we presume smaller segmentations would not help.

Additionally we compared the Min/Mean/Max strategies for COSMIC and ENSEMBL. The following paragraph has been added to the results:

"Similarly, considering different aggregation strategies for COSMIC and ENSEMBL has not affected the results significantly: for COSMIC the results were Min: 90.94% Mean: 90.25%, Max: 90.05%. For ENSEMBL: Min: 87.44%, Mean: 89.92%, Max: 89.54%."
Reviewer #3:
Streck and Schwarz present a method, CNSintent, for consistent segmentation of copy-number data. The utility of the tool is demonstrated using three large cancer cohorts and a neural network classifier built upon the consistently segmented data. CNSintent can facilitate solving an important biomedical problem: the advanced analysis of copy-number data. The authors are lauded for their excellent Python code and thorough documentation. While the contribution is timely and likely important, there are several areas for improvement.

The manuscript's readability could be better. There are typos, textual errors, and inconsistencies in figure captions, such as incorrect figure references or mismatched values between the text and figures. The "Consistent Segmentation" section is difficult to follow. It is unclear whether this step involves merging pre-existing breakpoints in the data to produce new, longer segments or if larger segments, such as whole chromosomes, are split into smaller, constant-sized segments. The writing suggests that segments are first merged and then split; however, later in the manuscript, they appear to be used separately. In our testing, combining these approaches did not yield meaningful results. Since consistent segmentation is the method's most critical step, we strongly suggest clarifying this section.

Response: We apologise if the workflow was not fully clear. Both merging and splitting are optional. While technically doing both consecutively is possible, i.e. it is possible to first merge the breakpoints and then subdivide newly created regions, this is not very common in practice and we did not use both steps at the same time. We have updated the first paragraph as follows:

"Segmentation consists of the following 4 steps: (i) define regions of interest (e.g. whole chromosomes, coding genes, etc.), (ii) remove exclusion regions (e.g. telomeric or centromeric regions), (iii) share existing breakpoints between samples and merge them based on a distance threshold, and/or (iv) subdivide the segments into fixed-width bins. Each of the four steps is optional."

The Results section now describes the segmentations as follows:

"We next set out to explore the effects of different segmentation strategies on the cancer classification task

(see Methods). We processed the data using the following segmentation strategies: (i) fixed-size segments of 20 Mb, 10Mb, 5 Mb, 3 Mb, 2 Mb, 1 Mb, 500 Kb, 250 Kb, 100 Kb size; (ii) Whole chromosomes, chromosome arms; (iii) Gene-level CN values based on the ENSEMBL and COSMIC gene sets; and (iv) breakpoint merging using distance thresholds of 1 Mb, 500 Kb, and 250 Kb. The segment sizes roughly cover the ranges used by other authors for feature discovery (Drews et al. 2022)."

We hope that improves the clarity of the process.

The manuscript is unbalanced in its content, with excessive focus on the tool's application and the discoveries derived from it, rather than on the tool itself. This reduces the clarity of the key message. We recommend compressing the application section (deep learning in cancer classification) while expanding the tool description with additional explanations.

Response: We thank the Reviewer for this comment and while we would have loved to reduce the application part of the manuscript in favor of a more detailed explanation of the tool itself, both Reviewer 1 and Reviewer 2 asked for additional data analyses, so we had to find a compromise. In line with your request and also Reviewer 1, we have removed the Integrated Gradients Method altogether and replaced it with a more compact subsection of statistical analysis (section title: Identifying commonly altered regions and outliers) instead. We have also removed not strictly necessary parts including the UMAP analysis, and added more general examples of the application of CNSistent instead. We hope that this improves upon the clarity of the main message of the paper. We have also overall streamlined the application section and edited it for brevity, to further improve on this point.

It is also unclear what type of data the authors are using in the cancer classification section. To improve clarity, this information should be explicitly included in the methods section, detailing the sequencing strategy and copy-number tools used for each cohort.

Response: We have added the following to the data availability:

"For TCGA the ASCAT team called the CNs by ASCATv3 from WGS, the TRACERx team used ASCATv2 from WES and PCAWG consortium published CNs obtained as a consensus of 5 different callers (PCAWG Consortium 2020) from WGS."

The methods section would benefit from a more detailed explanation of the CNSintent steps. Both Figure 1 and the text leave some parts unclear, particularly in the "Consistent Segmentation" section. Additionally, methods such as random forest and UMAP are only briefly mentioned in a supplementary figure rather than being described in the methods section. Moving these descriptions to the methods section would improve clarity.

Response: We have now clarified the Methods section and updated Figure 1 as described in the first comment. We have removed the UMAP analysis as we felt it does not provide enough interesting additional information and it is not part of the CNSistent package, in line with your comment #1. Since the Random Forest method is widely used in the field and not specific to the task we consider here, we decided in the interest of space to not provide a technical description of the method. We detail information about which version of the corresponding Python libraries was used in which part of the analysis in the Methods section:

"The splitting is done using the StratifiedGroupKFold object from scikit-learn v1.4.1, which was also used for ENet and RF classifiers.
For ENet we used the SGDClassifier with log loss and the elasticnet penalty. For RF we used RandomForestClassifier with default parameters."

Figures are generally clear, but improving color differentiation would be beneficial. For example, in Figure 1, the dark red and dark orange shades are too similar, making them difficult to distinguish. A more optimized color scheme with slightly lighter tones (i.e., increased luminance) would enhance readability.

Response: We agree with the point and we have changed the colors accordingly, as shown below. The dark red and dark orange are now "tab:red" and "tab:purple" respectively. We have also switched all plots to a

standard unified palette with more discernible colors. We hope that this improves upon the readability and accessibility of the Figures.

The introduction promotes copy-number signatures; however, these signatures rely on segment lengths and unique breakpoints, which vary between samples. Since this method enforces consistent segmentation and breakpoints across all samples, its applicability to copy-number signatures is unclear. This should be discussed in the Discussion section or removed from the introduction.

We agree with the reviewer that our original intention to promote the use of CN signatures has not been reflected in further sections. CNSistent can generally produce features used for detection of signatures, e.g. breakpoint counts or step sizes, however we have in the end did not develop this to a full extent. Consequently, we have removed mentions of CN signatures from the manuscript altogether.

Out of curiosity: Is it possible to prioritize one type of segmentation over another? For instance, if both WGS and WES data are available, can CNSintent be configured to prioritize WGS calls? Similarly, some tools provide highly precise breakpoint calls that are valuable for detecting fusion genes or rearrangements. In such cases, it would be useful to prioritize these calls and harmonize results from other tools accordingly.

We thank the Reviewer for this suggestion, which is a really interesting one. Unfortunately, currently CNSistent does not support such a weighting of breakpoints. When discussing a potential implementation we realised that there are quite some technical details to be decided upon that would depend from use case to use case and that this is actually a somewhat larger feature that we would like to postpone for the next version of the package.

Terminology Clarifications:

Blacklist, blacklisted regions, gap regions, mask: These terms should be used consistently, particularly since blacklists can be applied at different processing stages. Notably, PCAWG blacklists samples, not regions.

Response: Thank you for the suggestions. To make the text consistent we have used these three exclusive terms:
Gaps refers to the gap regions table as defined by UCSC genome browser. We retained this term without changes.
Region exclusion refers to the process of removing specific regions from downstream analysis. Now defined in methods “Optionally, exclusion regions can be provided to the pipeline to remove locations in the genome where we expect lower quality of information.” All occurrences of the term blacklisting in this context have been removed.
Blacklisting refers to removal for samples from the PCAWG dataset which were not marked as “whitelisted”. We retained this term without changes.

Segmentation: The term is commonly used in CNV analysis to refer to inferring continuous genomic segments from raw read counts or probe intensities. Here, it has a slightly different meaning—computing consistent breakpoints across all samples—so a more explicit definition would be helpful.

Response: Thank you for the comment, we decided to declare the meaning explicitly in the Methods section to avoid any confusion:

“Note that we use the term segmentation to refer to a consistent segmentation between samples, i.e. a set of positions inside each chromosome that split the chromosome into segments.”

Breakpoint merging/clustering: If these terms are synonymous, choosing one would improve readability.

Response: Thank you for pointing the issue out. To avoid any further confusion, we have completely removed the term clustering, and now use “merging” throughout the manuscript..

Coverage: Since "coverage" often refers to sequencing depth, a critical quality metric in DNA sequencing, it might be clearer to use "copy-number coverage" or a similar term. For example, the sentence "Next, samples with low coverage were removed using the…" could be ambiguous if read without context.

Response: We agree with the possibility of confusion. To prevent it, we have everywhere replaced the term “coverage” with a form of the suggested: “CN-coverage”.

At the end of the subsection "Explainability and the Effect of SOX2 Gene," the phrase "which exhibits significant local amplification in LUSC" should be revised to "which exhibits significant focal amplification in LUSC." The correct terminology is "focal" rather than "local," as established in Beroukhim et al. (2010).

Response: Thank you for the correction. Due to the changes to this section, the original sentence has been removed altogether.

Editorial Manager®

## Cookie Preference Center

We use cookies which are necessary to make our site work. We may also use additional cookies to analyze, improve and personalize our content and your digital experience. For more information, see our Cookie Policy.

You may choose not to allow some types of cookies. However, blocking some types may impact your experience of our site and the services we are able to offer. See the different category headings below to find out more or change your settings.

Allow all

## Manage Consent Preferences

### Strictly Necessary Cookies

**Always active**

These cookies are necessary for the website to function and cannot be switched off in our systems. They are usually only set in response to actions made by you which amount to a request for services, such as setting your privacy preferences, logging in or filling in forms. You can set your browser to block or alert you about these cookies, but some parts of the site will not then work.

### Functional Cookies

These cookies enable the website to provide enhanced functionality and personalisation. They may be set by us or by third party providers whose services we have added to our pages. If you do not allow these cookies then some or all of these services may not function properly.

### Performance Cookies

These cookies allow us to count visits and traffic sources so we can measure and improve the performance of our site. They help us to know which pages are the most and least popular and see how visitors move around the site.

### Targeting Cookies

These cookies may be set through our site by our advertising partners. They may be used by those companies to build a profile of your interests and show you relevant adverts on other sites. If you do not allow these cookies, you will experience less targeted advertising.

## Cookie List

Clear

Apply Cancel

Consent Leg.Interest

Confirm my choices

Powered by onetrust
