# Supplementary material for: CNSistent integration and feature extraction from somatic copy number profiles
Source: Gigascience. 2025 Sep 13;14:giaf104. doi: 10.1093/gigascience/giaf104 (PMC12448185; doi:10.1093/gigascience/giaf104)
Supplement: giaf104_Authors_Response_To_Reviewer_Comments_Revision_1 [file giaf104_authors_response_to_reviewer_comments_revision_1.pdf]

Author's Response To Reviewer Comments

Below please find the text-only response to reviews. For the version with included images and text formatting, please see the document "CNSistent_Reponse_To_Reviews_2.pdf".

Reviewer #1:

The only comment I'd make is with point 6. Metadata effects: Age influences the copy number alterations. The authors don't consider age or any other metadata and their implication in the classification task.

I agree with the authors that controlling for age is non-trivial. However, explicitly including it as a covariate could introduce collider bias (i.e if age is correlated with a specific cancer type it will be highly predictive even if it's effect on copy number profiles are trivial). The commentary around predictive accuracy is also not that useful as (for e.g) age might be highly predictive for one of the classes but not the others- accuracy here will not reflect this.

In some ways if prediction is the end goal it doesn't really matter if CNA profiles have age signatures that improve this prediction. Of course, if interpretation is the end goal, then understanding age specific factors is important. In this case, probably what would be required is some sort of model that predicts copy number at each genomic window- with a cancer type specific age covariate, a global age covariate and a cancer type covariate. Probably the age covariates would be shared across all genomic windows (or maybe could be chromosome specific but likely window specific would be too fine grained). This could be interesting future work to explore but is not necessary for this work.

We would like to thank Reviewer #2 for taking on the responsibility of also taking care of the responses to the comments from Reviewer #1. To observe if there is indeed not a trivial bias introduced through age as suggested by the reviewer, we have tested 1-vs-all binary linear classifiers on balanced datasets (also now included with the code) for the top 6 cancer types we use in classification, i.e. for each class we selected all the samples in the class, and a random subsample of the remaining classes of the same size and attempted to distinguish between the two using age as the only classification variable. The results were the following:

KIRC: Accuracy = 0.5278
OV: Accuracy = 0.5934
BRCA: Accuracy = 0.5972
LUAD: Accuracy = 0.5930
LUSC: Accuracy = 0.6154
PRAD: Accuracy = 0.5764

These data suggest that age is not highly predictive of any specific cancer type in our cohort. Looking at the individual distributions we can see that the only apparent distinction is between lung cancers and the rest, as seen on the reviewer figure, which we reproduced below:

[Reviewer Figure 1]

We therefore do not believe age to be a direct confounder in our case.

We also investigated the relationship between age and copy number alterations in more detail, using the number of breakpoints in a sample as a proxy for the number of structural variants. If there was a strong effect of age on the copy number landscape, we should see a correlation between the number of breakpoints and age, however we did not find a strong correlation in the 12009 samples tested. We have added the result of this analysis as a new Supp. Figure 7, reproduced here:

[Supp. Figure 7]

To reflect these thoughts we have inserted the following paragraph in the Results section:

“We only considered segments on autosomes as the sex of the patient acts as a confounder, in particular for BRCA, OV, and PRAD. To evaluate any possible confounding effect of age we compared the number of breakpoints to the age of the patients across the cohort (n=12009), and found it to only have a minor effect (r=0.11, Supp. Fig. 7). Similarly, age alone was a poor predictor of cancer type, achieving a mean test accuracy on a class-balanced one-versus-all linear classifier of 59.03% and a validation accuracy for the multi-class linear classifier of 31.78%.”

The above is of course an analysis of a linear relationship between the age and the alterations. Non-linear relationships are likely to exist, although it is not clear to us to what extent, however we felt that a deeper analysis of the topic goes beyond the scope of the article.

Reviewer #2: The authors have done a great job of addressing reviewer comments, adding further analysis and making the manuscript clearer.

(Very) Minor comments:

"To conduct such analyses however SCNA profiles have to be integrated between samples, patients, and cohorts, an often non-trivial task, for which dedicated toolkits are lacking." - This abstract sentence is clunky/poorly punctuated.

We have changed the word order to the following: However, to conduct such analyses SCNA profiles have to be integrated between samples, patients, and cohorts—often a non-trivial task, for which dedicated toolkits are lacking.

"The following was not declared in the manuscript and therefore has been set to default PyTorch values" - maybe change this to directly reference the manuscript in question (i.e Attique et al?)"

As suggested, we have changed the sentence to: The following was not declared in Attique et al.18 and therefore has been set to default PyTorch values.

"We have introduced CNSistent, a new Python-based library for processing and exploratory data analysis of SCNA profiles. Using the PCAWG, TCGA, and TRACERx datasets." Punctuation

We thank the reviewer for spotting the issue. We have corrected the text to the following: We have introduced CNSistent, a new Python-based library for processing and exploratory data analysis of SCNA profiles and applied it to the PCAWG, TCGA, and TRACERx datasets.

Discussion, suggest for:
Unfortunately, the comparison of our models to the one from Attique et al18 was partially limited by the fact that the authors did neither provide all of the model parameters, nor the model source code, and that the source dataset was no longer available at the time of writing this article. Validation on a hold-out set or cross-validation has not been conducted by the authors, we therefore only performed our comparisons on the test accuracies. All models and the input dataset used in this study are available online (see Data and code availability section).

Something like:
"We adapted the CNN originally introduced in Attique et al, and showed superior performance, with the caveat that the original model parameters, source code and source dataset were not available at the time of writing this article. All models and the input dataset used in this study are available online (see Data and code availability section)." And change the location of "Validation on a hold-out set or cross-validation has not been conducted by the authors, we therefore only performed our comparisons on the test accuracies." to be in the results section, where you compare to their results directly.

We agree with the reviewer and we have updated the suggested sentence with minor modifications. The updated Discussion now contains: “We adapted the CNN and DNN3 models originally introduced in Attique

et al18 and showed superior performance. The comparison is however limited, since the original model parameters, source code and source dataset were not available at the time of writing this article. All models and the input dataset used in this study are available online (see Data and code availability section).”

The second part we placed in the Results section as follows: “Validation on a hold-out set or cross-validation has not been conducted by the authors, we therefore only performed our comparisons on the test accuracies. The best test accuracy (maximum of 5 folds) was 92.42% on 1 Mb segments with the CNN+ mode, slightly above the best test accuracy of Attique et al. (92%).”

Typo/grammar:
"which likewise all additionally we used a normalized Manhattan Distances score to detect outlier samples"

We thank the reviewer for noticing the error, we corrected the text to the following: …which are likewise all located on chromosome 3. Additionally, we used the normalized Manhattan Distances score to detect outlier samples…

Reviewer #3: The revised is improved and much more readable. No further comments.

Editorial Manager®

Cookie Preference Center

We use cookies which are necessary to make our site work. We may also use additional cookies to analyze, improve and personalize our content and your digital experience. For more information, see our Cookie Policy.

You may choose not to allow some types of cookies. However, blocking some types may impact your experience of our site and the services we are able to offer. See the different category headings below to find out more or change your settings.

Allow all

Manage Consent Preferences

Strictly Necessary Cookies

Always active

These cookies are necessary for the website to function and cannot be switched off in our systems. They are usually only set in response to actions made by you which

amount to a request for services, such as setting your privacy preferences, logging in or filling in forms. You can set your browser to block or alert you about these cookies, but some parts of the site will not then work.

Functional Cookies

These cookies enable the website to provide enhanced functionality and personalisation. They may be set by us or by third party providers whose services we have added to our pages. If you do not allow these cookies then some or all of these services may not function properly.

Performance Cookies

These cookies allow us to count visits and traffic sources so we can measure and improve the performance of our site. They help us to know which pages are the most and least popular and see how visitors move around the site.

Targeting Cookies

These cookies may be set through our site by our advertising partners. They may be used by those companies to build a profile of your interests and show you relevant adverts on other sites. If you do not allow these cookies, you will experience less targeted advertising.

Cookie List

Clear

Apply Cancel

Consent Leg.Interest

Confirm my choices

Powered by onetrus
