# Supplementary material for: CNSistent integration and feature extraction from somatic copy number profiles
Source: Gigascience. 2025 Sep 13;14:giaf104. doi: 10.1093/gigascience/giaf104 (PMC12448185; doi:10.1093/gigascience/giaf104)
Supplement: giaf104_Reviewer_1_Report_Original_Submission — Stefano Monti -- 2/9/2025 [file giaf104_reviewer_1_report_original_submission.pdf]

Reviewer Report

Title: CNSistent integration and feature extraction from somatic copy number profiles

Version: Original Submission **Date:** 2/9/2025

Reviewer name: Stefano Monti

Reviewer Comments to Author:

This is a well-written paper that aims to develop a tool that can integrate SCNA from large datasets possibly generated using different platforms to identify alteration patterns that are often undetected in smaller data subsets. Authors have used CNN-based method for integrating the data, extracting features and predicting cancer types from SCNA profiles. The tool has the potential to significantly simplify the integration and analysis of large scale SCNA studies. However, some (hopefully addressable) weaknesses are noted:

1. The choice of a classification task as the (only) way to evaluate the proposed method is questioned. I would argue that the most important use of SCNA detection is in support of mechanistic investigations, by identifying novel candidate loci likely to harbor tumor suppressors (copy losses) and oncogenes (copy gains). This type of analysis is hardly mentioned in the manuscript, and it is not clear how well the proposed tool would support it. I surmise it can, but the authors should discuss (and present results about) it.
2. If we were to focus on the task of recurrent SCNA detection, then meta-analysis approaches (where separate analyses are performed on each of the datasets, and only the results are integrated) would need to be considered as an alternative to the approach here proposed (e.g., application of GISTIC to each of PCAWG, TCGA, TRACERx separately, followed by meta-analysis integration of the results). I am not saying meta-analysis would be superior, but the authors should discuss it, and possibly evaluate it.
3. The reported metrics to quantify the quality of the integration are insufficient to assess the results. There is some lack of clarity about the classification accuracy results reported, since it is not clear whether all the components of the model building were adequately brought into the cross-validation (or train/test) loop. More specifically, when reporting the accuracy of the cancer type classification, it is reported that 1 megabase segmentation yields the best results. It is not clear if this size selection was performed within the train set only (and/or within the CV loop) or across the entire dataset. If the latter, this may significantly affect the accuracy results, which could not be deemed (unbiased) "test set" results. This should be clarified, and if the segment size selection was indeed performed outside the train/test split, accuracy measures should be computed again by performing the segment size selection properly (which of course it would mean a potentially different size would be selected for each of the folds).
4. Comparisons with other methods: The authors only compare their method to random forest (RF). Related to the previous point: I presume the RF model used the segment size that was optimized for the CNN model (i.e., 1Mb). If this is the case, it would be an unfair comparison, since RF might favor a different size. Also, additional classifiers should be evaluated (e.g., Elastic Net, SVM, etc.).
5. There is no sufficient discussion of existing tools/methods. This should be corrected (see also my

comment about meta-analysis approaches).
6. Metadata effects: Age influences the copy number alterations. The authors don't consider age or any other metadata and their implication in the classification task.
7. Run time statistics and user requirement: While the authors report runtime curves per command (S Fig 6), it is difficult to translate this to total runtime. It would be useful if runtime for the entire training of a model were reported. Additionally, if available, comparison of run time stats with the established model that they cite would be useful.
8. IG-based explanation. I found this section sort of perfunctory, not sufficiently justified, and adding little to the manuscript. IG is computationally expensive, and it does not provide any way to statistically quantify the found associations. Simpler methods, such as testing for association between SCNA occurrence and cancer type should be evaluated and compared to.
9. Model selection: No adequate justification of why they picked CNN for this task when the referenced paper itself claims the DNN architecture performs better. Not sure but is this because of the varying segment size? Again, this is not clearly stated.
https://pmc.ncbi.nlm.nih.gov/articles/PMC9203194/#tab1

Methods

Are the methods appropriate to the aims of the study, are they well described, and are necessary controls included? Choose an item.

Conclusions

Are the conclusions adequately supported by the data shown? Choose an item.

Reporting Standards

Does the manuscript adhere to the journal's guidelines on minimum standards of reporting? Choose an item.

Choose an item.

Statistics

Are you able to assess all statistics in the manuscript, including the appropriateness of statistical tests used? Choose an item.

Quality of Written English

Please indicate the quality of language in the manuscript: Choose an item.

Declaration of Competing Interests

Please complete a declaration of competing interests, considering the following questions:

- Have you in the past five years received reimbursements, fees, funding, or salary from an organisation that may in any way gain or lose financially from the publication of this manuscript, either now or in the future?
- Do you hold any stocks or shares in an organisation that may in any way gain or lose financially from the publication of this manuscript, either now or in the future?
- Do you hold or are you currently applying for any patents relating to the content of the manuscript?
- Have you received reimbursements, fees, funding, or salary from an organization that holds or has applied for patents relating to the content of the manuscript?
- Do you have any other financial competing interests?
- Do you have any non-financial competing interests in relation to this paper?

If you can answer no to all of the above, write 'I declare that I have no competing interests' below. If your reply is yes to any, please give details below.

I declare that I have no competing interests

I agree to the open peer review policy of the journal. I understand that my name will be included on my report to the authors and, if the manuscript is accepted for publication, my named report including any attachments I upload will be posted on the website along with the authors' responses. I agree for my report to be made available under an Open Access Creative Commons CC-BY license (http://creativecommons.org/licenses/by/4.0/). I understand that any comments which I do not wish to be included in my named report can be included as confidential comments to the editors, which will not be published.

Choose an item.

To further support our reviewers, we have joined with Publons, where you can gain additional credit to further highlight your hard work (see: https://publons.com/journal/530/gigascience). On publication of this paper, your review will be automatically added to Publons, you can then choose whether or not to claim your Publons credit. I understand this statement.

Yes Choose an item.
