# Supplementary material for: CNSistent integration and feature extraction from somatic copy number profiles
Source: Gigascience. 2025 Sep 13;14:giaf104. doi: 10.1093/gigascience/giaf104 (PMC12448185; doi:10.1093/gigascience/giaf104)
Supplement: giaf104_Reviewer_2_Report_Original_Submission — Ellen Visscher -- 2/20/2025 [file giaf104_reviewer_2_report_original_submission.pdf]

Reviewer Report

Title: CNSistent integration and feature extraction from somatic copy number profiles

Version: Original Submission **Date:** 2/20/2025

Reviewer name: Ellen Visscher

Reviewer Comments to Author:

The paper introduces a python package for imputation, filtering, segmentation, feature extraction and visualisation of CNA profiles. It explains some of the elements of the package, and then demonstrates how data from multiple cohorts can be processed and combined using the package preprocessing pipeline. The authors then use processed data from 3 different cohorts to perform cancer type prediction using a CNN. From this, they get an interesting result to find a biomarker that differentiates two different lung cancers. Throughout, they show visualisations using their package. The package itself seems well documented and designed to be used. There is some clarification required in the methods section specifically around the CNN training and the models therein. There is also one major question of whether all the preprocessing steps are actually required for the downstream CNN analysis. Overall, however, this is a well written manuscript, providing a useful software tool for further analysis of CNA data.

Major comments:

- CNN section- how are the segments decided- is it based on all the training data, or just data in a batch?
- Throughout the results pertaining to figure 3A-C, you call it test accuracy- to be clear is this is based on your CV hold outs? This should be reworded everywhere to reflect this. As cross validation indicates, this is not a test set and is a validation set- which is also the way you use it.
- Regarding the above, you have a comment saying: "the best test accuracy without cross-validation was 92.34%". Could you please clarify what you mean by this. Only in the CNN section do you describe your training approach, which does not mention a test or separate validation set.
- It reads slightly unclearly- you have a section called "model transfer", but are you training 3 different models- one per dataset? You only have one figure for training results which suggests one dataset, but then you have this section called model transfer?
- Re all the above, please dedicate a small subsection in methods making this clearer. Are there dedicated test sets? If your main results are for aggregated data, then what are you testing on to ensure generalisability? What is the point of training the 3 different models on 3 different datasets? Perhaps it would make more sense to hold one dataset out as your test set. In some ways, that is what the model transfer is showing, but it would be less confusing to clarify that aim instead of suddenly introducing 3 models.
- If the CNN architecture is essentially the same as in Attique et. al., the performance is basically the same and they use only CNs a gene locations- how does this demonstrate that the preprocessing from CNSistent is necessary or advantageous for this task? Maybe having a result which combines CN calls naively over gene locations and comparing to this across the aggregate datasets would be a good way of comparing? I.e showing that preproccessing does offer an advantage when combining different datasets

together? Also because this is what you argue in your abstract. For this analysis you would have to make sure you also compare across the same samples to differentiate between filtering/other preprocessing steps.
- In Figure 3I, you say "notice the similarity of chromosome 3 pattern for the correctly classified LUSC samples (red) and the misclassified ones (orange)". This is confusing because the orange and red are not similar. In fact for this whole section, it seems that figure 3I does not align with what you are saying?
Minor comments/errors:
- Clarification on why CNSistent needs a reference genome if it's dealing with segments? How is this information used- is it just for the known gaps?
- Your caption of Supplementary Figure 1 has a typo about a breakpoint at 16 instead of 14.
- You do not explain how you use the knee pt to filter (i.e is it samples above/below the knee pt.)
- Your CNN graphic is difficult to interpret and non-standard.
- CNN section should clarify at the beginning what the input is and what the output is (i.e a prediction that a sample belongs to a particular cancer type) before explaining the architectural details.
- Even though you control for class imbalance, some cancer types are so poorly represented it is unlikely a CNN could learn that, you do kind of mention this in the discussion, but maybe some sort of minimum threshold for inclusion would make sense.
- For Fig2D you refer to it as GND, but the axes/title says hemizygosity-are these things equivalent? E.g could have 3-3, low hemizygosity but not diploid? Or if it's aggregated across the whole genome its assumed equivalent?
- There is a grammatical error "Runtimes decreased in a near-linearly with the number of compute cores"
- You make a comment that "We therefore suspect some TCGA lung cancers might be cases of co-occurring adeno and squamous carcinomas." This is a possibility but given pleiotropy of many phenotypes- it may also be that the biomarker is not always unique to squamous carcinomas.
Suggestions/Nice to haves:
- Maybe make it clearer inside the paper what visualisations come with CNSistent. Looking at the software documentation, there's obviously a lot of useful visualisations that come with that- and some of them you have used in Figure 3 for e.g.
- Given there are more total CN callers, maybe good to mention somewhere how CNSistent would work for total CNs only.
- You remove profiles that you say are uninformative, could you not include this and then just show how accuracy correlates with no. of break-pts (for e.g). In some ways one might think that there could be useful information in few alteration profiles- because those alterations might be more upstream/causal.
- The aggregation step could maybe affect downstream analysis. I.e taking the average could introduce CNs that were never called. Even using min/max- this implies a constant copy number in that region, which may lose information- e.g if it is a functional region having two diff CNs across gene might imply non-functionality. Did you explore the effect of aggregation step? Perhaps taking a small enough resolution of segment types would account for this anyway.

Methods

Are the methods appropriate to the aims of the study, are they well described, and are necessary controls included? Choose an item.

Conclusions

Are the conclusions adequately supported by the data shown? Choose an item.

Reporting Standards

Does the manuscript adhere to the journal's guidelines on minimum standards of reporting? Choose an item.

Choose an item.

Statistics

Are you able to assess all statistics in the manuscript, including the appropriateness of statistical tests used? Choose an item.

Quality of Written English

Please indicate the quality of language in the manuscript: Choose an item.

Declaration of Competing Interests

Please complete a declaration of competing interests, considering the following questions:

- Have you in the past five years received reimbursements, fees, funding, or salary from an organisation that may in any way gain or lose financially from the publication of this manuscript, either now or in the future?
- Do you hold any stocks or shares in an organisation that may in any way gain or lose financially from the publication of this manuscript, either now or in the future?
- Do you hold or are you currently applying for any patents relating to the content of the manuscript?
- Have you received reimbursements, fees, funding, or salary from an organization that holds or has applied for patents relating to the content of the manuscript?
- Do you have any other financial competing interests?
- Do you have any non-financial competing interests in relation to this paper?

If you can answer no to all of the above, write 'I declare that I have no competing interests' below. If your reply is yes to any, please give details below.

I declare that I have no competing interests

I agree to the open peer review policy of the journal. I understand that my name will be included on my report to the authors and, if the manuscript is accepted for publication, my named report including any attachments I upload will be posted on the website along with the authors' responses. I agree for my report to be made available under an Open Access Creative Commons CC-BY license (http://creativecommons.org/licenses/by/4.0/). I understand that any comments which I do not wish to be included in my named report can be included as confidential comments to the editors, which will not be published.

Choose an item.

To further support our reviewers, we have joined with Publons, where you can gain additional credit to further highlight your hard work (see: https://publons.com/journal/530/gigascience). On publication of this paper, your review will be automatically added to Publons, you can then choose whether or not to claim your Publons credit. I understand this statement.

Yes Choose an item.
