# Supplementary material for: CNSistent integration and feature extraction from somatic copy number profiles
Source: Gigascience. 2025 Sep 13;14:giaf104. doi: 10.1093/gigascience/giaf104 (PMC12448185; doi:10.1093/gigascience/giaf104)
Supplement: giaf104_Reviewer_2_Report_Revision_1 — Ellen Visscher -- 6/11/2025 [file giaf104_reviewer_2_report_revision_1.pdf]

Reviewer Report

Title: CNSistent integration and feature extraction from somatic copy number profiles

Version: Revision 1 **Date:** 6/11/2025

Reviewer name: Ellen Visscher

Reviewer Comments to Author:

The authors have done a great job of addressing reviewer comments, adding further analysis and making the manuscript clearer.
(Very) Minor comments:
"To conduct such analyses however SCNA profiles have to be integrated between samples, patients, and cohorts, an often non-trivial task, for which dedicated toolkits are lacking." - This abstract sentence is clunky/poorly punctuated.
"The following was not declared in the manuscript and therefore has been set to default PyTorch values" - maybe change this to directly reference the manuscript in question (i.e Attique et al?)"
"We have introduced CNSistent, a new Python-based library for processing and exploratory data analysis of SCNA profiles. Using the PCAWG, TCGA, and TRACERx datasets." Punctuation
Discussion, suggest for:
Unfortunately, the comparison of our models to the one from Attique et al18 was partially limited by the fact that the authors did neither provide all of the model parameters, nor the model source code, and that the source dataset was no longer available at the time of writing this article. Validation on a hold-out set or cross-validation has not been conducted by the authors, we therefore only performed our comparisons on the test accuracies. All models and the input dataset used in this study are available online (see Data and code availability section).
Something like:
"We adapted the CNN originally introduced in Attique et al, and showed superior performance, with the caveat that the original model parameters, source code and source dataset were not available at the time of writing this article. All models and the input dataset used in this study are available online (see Data and code availability section)." And change the location of "Validation on a hold-out set or cross-validation has not been conducted by the authors, we therefore only performed our comparisons on the test accuracies." to be in the results section, where you compare to their results directly.
Typo/grammar:
"which likewise all additionally we used a normalized Manhattan Distances score to detect outlier samples"

Methods

Are the methods appropriate to the aims of the study, are they well described, and are necessary controls included? Choose an item.

Conclusions

Are the conclusions adequately supported by the data shown? Choose an item.

Reporting Standards

Does the manuscript adhere to the journal's guidelines on minimum standards of reporting? Choose an item.

Choose an item.

Statistics

Are you able to assess all statistics in the manuscript, including the appropriateness of statistical tests used? Choose an item.

Quality of Written English

Please indicate the quality of language in the manuscript: Choose an item.

Declaration of Competing Interests

Please complete a declaration of competing interests, considering the following questions:

- Have you in the past five years received reimbursements, fees, funding, or salary from an organisation that may in any way gain or lose financially from the publication of this manuscript, either now or in the future?
- Do you hold any stocks or shares in an organisation that may in any way gain or lose financially from the publication of this manuscript, either now or in the future?
- Do you hold or are you currently applying for any patents relating to the content of the manuscript?
- Have you received reimbursements, fees, funding, or salary from an organization that holds or has applied for patents relating to the content of the manuscript?
- Do you have any other financial competing interests?
- Do you have any non-financial competing interests in relation to this paper?

If you can answer no to all of the above, write 'I declare that I have no competing interests' below. If your reply is yes to any, please give details below.

I declare that I have no competing interests

I agree to the open peer review policy of the journal. I understand that my name will be included on my report to the authors and, if the manuscript is accepted for publication, my named report including any attachments I upload will be posted on the website along with the authors' responses. I agree for my

report to be made available under an Open Access Creative Commons CC-BY license (http://creativecommons.org/licenses/by/4.0/). I understand that any comments which I do not wish to be included in my named report can be included as confidential comments to the editors, which will not be published.

Choose an item.

To further support our reviewers, we have joined with Publons, where you can gain additional credit to further highlight your hard work (see: https://publons.com/journal/530/gigascience). On publication of this paper, your review will be automatically added to Publons, you can then choose whether or not to claim your Publons credit. I understand this statement.

Yes Choose an item.
