# Supplementary material for: CNSistent integration and feature extraction from somatic copy number profiles
Source: Gigascience. 2025 Sep 13;14:giaf104. doi: 10.1093/gigascience/giaf104 (PMC12448185; doi:10.1093/gigascience/giaf104)
Supplement: giaf104_Reviewer_3_Report_Original_Submission — Sampsa Hautaniemi -- 3/11/2025 [file giaf104_reviewer_3_report_original_submission.pdf]

Reviewer Report

Title: CNSistent integration and feature extraction from somatic copy number profiles

Version: Original Submission **Date:** 3/11/2025

Reviewer name: Sampsa Hautaniemi

Reviewer Comments to Author:

Streck and Schwarz present a method, CNSintent, for consistent segmentation of copy-number data. The utility of the tool is demonstrated using three large cancer cohorts and a neural network classifier built upon the consistently segmented data. CNSintent can facilitate solving an important biomedical problem: the advanced analysis of copy-number data. The authors are lauded for their excellent Python code and thorough documentation. While the contribution is timely and likely important, there are several areas for improvement.

The manuscript's readability could be better. There are typos, textual errors, and inconsistencies in figure captions, such as incorrect figure references or mismatched values between the text and figures. The "Consistent Segmentation" section is difficult to follow. It is unclear whether this step involves merging pre-existing breakpoints in the data to produce new, longer segments or if larger segments, such as whole chromosomes, are split into smaller, constant-sized segments. The writing suggests that segments are first merged and then split; however, later in the manuscript, they appear to be used separately. In our testing, combining these approaches did not yield meaningful results. Since consistent segmentation is the method's most critical step, we strongly suggest clarifying this section.

The manuscript is unbalanced in its content, with excessive focus on the tool's application and the discoveries derived from it, rather than on the tool itself. This reduces the clarity of the key message. We recommend compressing the application section (deep learning in cancer classification) while expanding the tool description with additional explanations.

It is also unclear what type of data the authors are using in the cancer classification section. To improve clarity, this information should be explicitly included in the methods section, detailing the sequencing strategy and copy-number tools used for each cohort.

The methods section would benefit from a more detailed explanation of the CNSintent steps. Both Figure 1 and the text leave some parts unclear, particularly in the "Consistent Segmentation" section. Additionally, methods such as random forest and UMAP are only briefly mentioned in a supplementary figure rather than being described in the methods section. Moving these descriptions to the methods section would improve clarity.

Figures are generally clear, but improving color differentiation would be beneficial. For example, in Figure 1, the dark red and dark orange shades are too similar, making them difficult to distinguish. A more optimized color scheme with slightly lighter tones (i.e., increased luminance) would enhance readability.

The introduction promotes copy-number signatures; however, these signatures rely on segment lengths and unique breakpoints, which vary between samples. Since this method enforces consistent segmentation and breakpoints across all samples, its applicability to copy-number signatures is unclear.

This should be discussed in the Discussion section or removed from the introduction.
Out of curiosity: Is it possible to prioritize one type of segmentation over another? For instance, if both WGS and WES data are available, can CNSintent be configured to prioritize WGS calls? Similarly, some tools provide highly precise breakpoint calls that are valuable for detecting fusion genes or rearrangements. In such cases, it would be useful to prioritize these calls and harmonize results from other tools accordingly.
Terminology Clarifications:
Blacklist, blacklisted regions, gap regions, mask: These terms should be used consistently, particularly since blacklists can be applied at different processing stages. Notably, PCAWG blacklists samples, not regions.
Segmentation: The term is commonly used in CNV analysis to refer to inferring continuous genomic segments from raw read counts or probe intensities. Here, it has a slightly different meaningâ€”computing consistent breakpoints across all samplesâ€”so a more explicit definition would be helpful.
Breakpoint merging/clustering: If these terms are synonymous, choosing one would improve readability.
Coverage: Since "coverage" often refers to sequencing depth, a critical quality metric in DNA sequencing, it might be clearer to use "copy-number coverage" or a similar term. For example, the sentence "Next, samples with low coverage were removed using the…" could be ambiguous if read without context.
At the end of the subsection "Explainability and the Effect of SOX2 Gene," the phrase "which exhibits significant local amplification in LUSC" should be revised to "which exhibits significant focal amplification in LUSC." The correct terminology is "focal" rather than "local," as established in Beroukhim et al. (2010).

Methods

Are the methods appropriate to the aims of the study, are they well described, and are necessary controls included? Choose an item.

Conclusions

Are the conclusions adequately supported by the data shown? Choose an item.

Reporting Standards

Does the manuscript adhere to the journal's guidelines on minimum standards of reporting? Choose an item.

Choose an item.

Statistics

Are you able to assess all statistics in the manuscript, including the appropriateness of statistical tests used? Choose an item.

Quality of Written English

Please indicate the quality of language in the manuscript: Choose an item.

Declaration of Competing Interests

Please complete a declaration of competing interests, considering the following questions:

- Have you in the past five years received reimbursements, fees, funding, or salary from an organisation that may in any way gain or lose financially from the publication of this manuscript, either now or in the future?
- Do you hold any stocks or shares in an organisation that may in any way gain or lose financially from the publication of this manuscript, either now or in the future?
- Do you hold or are you currently applying for any patents relating to the content of the manuscript?
- Have you received reimbursements, fees, funding, or salary from an organization that holds or has applied for patents relating to the content of the manuscript?
- Do you have any other financial competing interests?
- Do you have any non-financial competing interests in relation to this paper?

If you can answer no to all of the above, write 'I declare that I have no competing interests' below. If your reply is yes to any, please give details below.

I declare that I have no competing interests.

I agree to the open peer review policy of the journal. I understand that my name will be included on my report to the authors and, if the manuscript is accepted for publication, my named report including any attachments I upload will be posted on the website along with the authors' responses. I agree for my report to be made available under an Open Access Creative Commons CC-BY license (http://creativecommons.org/licenses/by/4.0/). I understand that any comments which I do not wish to be included in my named report can be included as confidential comments to the editors, which will not be published.

Choose an item.

To further support our reviewers, we have joined with Publons, where you can gain additional credit to further highlight your hard work (see: https://publons.com/journal/530/gigascience). On publication of this paper, your review will be automatically added to Publons, you can then choose whether or not to claim your Publons credit. I understand this statement.

Yes Choose an item.
